# Chitosan Induces Plant Hormones and Defenses in Tomato Root Exudates

**DOI:** 10.3389/fpls.2020.572087

**Published:** 2020-11-04

**Authors:** Marta Suarez-Fernandez, Frutos Carlos Marhuenda-Egea, Federico Lopez-Moya, Marino B. Arnao, Francisca Cabrera-Escribano, Maria Jose Nueda, Benet Gunsé, Luis Vicente Lopez-Llorca

**Affiliations:** ^1^Laboratory of Plant Pathology, Multidisciplinary Institute for Environmental Studies Ramon Margalef, University of Alicante, Alicante, Spain; ^2^Department of Marine Sciences and Applied Biology, Laboratory of Plant Pathology, University of Alicante, Alicante, Spain; ^3^Department of Agrochemistry and Biochemistry, Multidisciplinary Institute for Environmental Studies Ramon Margalef, University of Alicante, Alicante, Spain; ^4^Department of Plant Biology (Plant Physiology), University of Murcia, Murcia, Spain; ^5^Department of Organic Chemistry, Chemistry Faculty, University of Seville, Seville, Spain; ^6^Department of Mathematics, University of Alicante, Alicante, Spain; ^7^Plant Physiology Laboratory, Faculty of Biosciences, Universidad Autonoma de Barcelona, Bellaterra, Spain

**Keywords:** chitosan, root exudates, membrane potential, lipid signaling, plant defenses, plant hormones, soil-borne pathogens

## Abstract

In this work, we use electrophysiological and metabolomic tools to determine the role of chitosan as plant defense elicitor in soil for preventing or manage root pests and diseases sustainably. Root exudates include a wide variety of molecules that plants and root microbiota use to communicate in the rhizosphere. Tomato plants were treated with chitosan. Root exudates from tomato plants were analyzed at 3, 10, 20, and 30 days after planting (dap). We found, using high performance liquid chromatography (HPLC) and excitation emission matrix (EEM) fluorescence, that chitosan induces plant hormones, lipid signaling and defense compounds in tomato root exudates, including phenolics. High doses of chitosan induce membrane depolarization and affect membrane integrity. ^1^H-NMR showed the dynamic of exudation, detecting the largest number of signals in 20 dap root exudates. Root exudates from plants irrigated with chitosan inhibit ca. twofold growth kinetics of the tomato root parasitic fungus *Fusarium oxysporum* f. sp. radicis-lycopersici. and reduced ca. 1.5-fold egg hatching of the root-knot nematode *Meloidogyne javanica*.

## Introduction

Chitosan is a linear polymer of β-(1-4)-linked *N*-acetyl-2-amino-2-deoxy-D-glucose (acetylated) and 2-amino-2-deoxy-D-glucose (deacetylated) (Kaur and Dhillon, 2014). It is generally obtained by partial deacetylation of chitin (Kumar, 2000), which is the second most abundant polysaccharide in nature after cellulose (Elieh-Ali-Komi and Hamblin, 2016). Chitin is a major component of the cuticle of insects, exoskeleton of crustaceans and fungal cell walls. Chitosan has been described as elicitor of plant defenses (Yin et al., 2016) and hormones of food security crops such as tomato (Iriti and Faoro, 2008; El-Tantawy, 2009). Chitosan also propitiates accumulation of auxin [mainly indoleacetic acid (IAA)] in the apex of plant roots (Lopez-Moya et al., 2017). Since most studies have been carried out in the phylloplane, we devised experiments to test the effect of chitosan on tomato rhizodeposition in both hydroponic and solid plant substrate systems.

Electrophysiology can monitor the response of plant roots to stress (Rodrigo-Moreno et al., 2013). Membrane potential reflects the action of all pumps in the cell membrane to maintain ion gradients (Alberts et al., 2002). Root cell membranes detect changes in their environment and respond starting metabolic cascade reactions (Fürstenberg-Hägg et al., 2013; Matzke and Matzke, 2013). Those reactions could lead to new compounds of agronomic and ecological interest.

Rhizodeposition is the process of releasing organic compounds from roots to the external medium. Plants exude a wide variety of low molecular weight organic compounds (e.g., amino acids and small peptides, organic acids, hormones, sugars, phenolics, and other secondary metabolites) (Flores et al., 1996). Resolving this complex mixture requires the use of diverse metabolomics technologies (Escudero et al., 2014; van Dam and Bouwmeester, 2016). Root exudates are paramount in plant–microbe interactions in the rhizosphere, including beneficial and pathogenic microbes and play a key role in signaling (Hirsch et al., 2003; Bais et al., 2006; Walker et al., 2009; Zhang et al., 2019; Valette et al., 2020). Metabolomics can help us to understand the chemical interactions between organisms in the rhizosphere, as well as the importance to uncover toxic compounds (Baldrian, 2019). Metabolomics allows detection of phenolic compounds by fluorescence (Hupp et al., 2019). Phenolics have diverse functions in plants, but one of the most important is their role in plant defense and signaling (Mandal et al., 2010). Hormones are also signaling molecules that can also be found using metabolomics (Street and Schenk, 1981; Li et al., 2009; Yang et al., 2015). Auxin (e.g., IAA), salicylic acid (SA), jasmonic acid (JA), and abscisic acid (ABA) are produced in response to physiologic or metabolic changes (Asami and Nakagawa, 2018). Phytomelatonin is considered a master regulator in plant stress conditions (Arnao and Hernández-Ruiz, 2019) and also involved in the regulation of several plant hormones (Arnao and Hernández-Ruiz, 2018). Phytomelatonin promotes growth and root appearance (Hernández-Ruiz et al., 2005; Arnao and Hernández-Ruiz, 2007).

Fungal (*Fusarium oxysporum* f. sp. radicis-lycopersici) and nematode (*Meloidogyne javanica*) root pathogens are used in this work. These pathogens threaten food security worldwide (FAO, 2017). Root-knot nematodes cause 25–100% tomato yield losses (Seid et al., 2015) and *Fusarium* wilt causes up to 80% of crop losses (Singh et al., 2017).

The aim of this work is to determine the effect of chitosan on plant rhizodeposition. Root electrophysiology allows us to monitor the effect of chitosan on membrane functionality. In addition, metabolomic techniques are used to determine how chitosan modulates the composition of root exudates. Finally, we test these exudates against root pathogens which threaten food security worldwide. This will allow us to validate the role of chitosan as a plant defense inducer in soil for preventing or managing root pests and diseases sustainably.

## Materials and Methods

### Chitosan, Plants, Fungi, and Nematodes

Chitosan with 70 kDa molecular weight and 80.5% deacetylation degree was used in all experiments. Chitosan was obtained from Marine BioProducts GmbH (Bremerhaven, Germany) and made as in Palma-Guerrero et al. (2007). Tomato plants (*Solanum lycopersicum* cv. Marglobe) were used in all experiments. *Pochonia chlamydosporia*, isolate number 123 (ATCC no. MYA-4875; CECT no. 20929; PRJNA68669, NCBI txid1052797) was isolated from *Heterodera avenae* eggs in south west Spain (Olivares and López-Llorca, 2002). *Fusarium oxysporum* f. sp. radicis-lycopersici (Strain 4287, CBS 123668, FGSC 9935, NRRL 34396) was obtained from CBS-KNAW culture collection. *Meloidogyne javanica* was obtained from a field population and maintained in susceptible tomato plants. Nematode egg masses were dissected from root-knot nematode infested roots and stored at 4°C. Egg masses were hand-picked and surface-sterilized as in McClure et al. (1973) with modifications.

### Root Electrophysiology Experiments

Tomato seeds were surface sterilized using 1% sodium hypochlorite and washed three times 1 min each with sterile distilled water (SDW). Tomato seeds were then placed on germination medium (GM, Glucose 10 g L^–1^, Yeast Extract 0.1 g L^–1^, Bactopeptone 0.1 g L^–1^, Technical Agar 12 g L^–1^). GM plates with tomato seeds were placed at 4°C for 2 days for stratification and incubated at 24°C, 65% relative humidity (RH), in the dark for 5 days and in a photoperiod (16:8) for further 5 days. Plantlets were then placed individually in a holder chamber filled with Gamborgs B5 1:10 (Sigma; Gamborg et al., 1968). Glass microcapillaries filled with 0.5M KCl (tip diameter < 1 μm) were inserted into root cortical cells until a stable basal potential was obtained (Gunsé et al., 2016). Roots were exposed to increasing concentrations of chitosan (0.1, 1, and 2 mg mL^–1^ in Gamborgs B5 1:10 medium) and membrane potentials were recorded. No chitosan was used for control treatments. Between each change of chitosan concentration, root medium was replaced for Gamborgs B5 1:10 to check the physiological status of the cell. At least three biological replicates and four technical replicates were performed per treatment.

### Root Vital Staining

Tomato plantlets were placed in Gamborgs B5 1:10 liquid medium 1 day for acclimatization. Chitosan was then added at 0.1, 1, and 2 mg mL^–1^. Plants exposed only to Gamborgs B5 1:10 were used as controls. Plantlets with treatments were incubated for 24 h. Roots were then stained with fluorescein diacetate (FDA), 5 mg mL^–1^ in acetone diluted 1:250 in Dulbecco’s phosphate-buffered saline (DPBS, Thermo Fisher) and propidium iodide (PI, 20 mg mL^–1^ in DPBS; Jones and Senft, 1985). Roots were visualized using a Nikon Optiphot microscope using 10X objective with an attached Nikon DS–5 M camera system using epifluorescence (Ex: 450–490 nm, Em: 520 nm). Non-damaged cells show green FDA fluorescence and nuclei of damaged cells show red PI fluorescence.

### Plant Growth Conditions

Two experiments were carried out: *in vitro* and in cups. This allowed us to progressively approach to a realistic system.

Tomato plantlets (Experiment 1) were placed in 200 mL expanded polystyrene sterile cups each containing 100 cm^3^ of sterilized sand. They were then incubated in a culture chamber (SANYO MLR-351H) at 65% RH, 24°C with a 16:8 h (light:dark) photoperiod. Plants were irrigated for 20 days with Gamborgs B5 basal mixture 1:10 keeping moisture to field capacity. Plants were then removed, washed and introduced individually in Magenta Boxes (575 mL, Sigma). Each Box was filled with 50 mL of Gamborgs B5 1:10 (control) or Gamborgs B5 1:10 amended with chitosan (0.1, 1, 2 mg mL^–1^). Nine biological replicates were made per treatment. Plants were incubated for 3 days as described above. Exudates collected in Experiment 1 are root exudates accumulated over 3 days. This experiment was performed two times independently.

In Experiment 2, tomato plantlets were grown as for Experiment 1. Plants in cups were then irrigated with 1:10 Gamborgs B5 basal mixture on its own or amended with 0.1 mg mL^–1^ chitosan. Plants were incubated as before. Ten biological replicates per treatment were made. Root Exudates were sampled 10, 20 and 30 days after planting (dap) for further analyses. Exudates collected in Experiment 2 are active root exudates (24 h collection, see section “Collection of Root Exudates”). This experiment was performed two times independently.

### Collection of Root Exudates

In Experiment 1, root exudates accumulated for 3 days in Magenta Boxes were collected and filtered through Miracloth (Calbiochem). Pools were made with the exudates of three plants each, so we worked with three biological replicates per treatment. This was performed twice. Root exudate pools were filtered by 0.22 μm (Q-MAX) and frozen at −20°C for further use.

In Experiment 2, whole plants were removed, and their root systems washed in SDW. *De novo* root exudates from these plants were collected by placing individual whole plants in sterile plastic containers with 20 mL SDW per gram of root. Plants were incubated in the dark at 24°C, 65% RH for 24 h. Plants were then removed, and root exudates from the 10 biological replicates were collected by 0.22 μm (Q-MAX) filtration and stored frozen at −20°C until used. This was performed twice.

### Emission Excitation Matrix (EEM) Fluorescence Analysis

Two mL of each root exudate (Experiments 1 and 2) were collected and excitation emission matrix (EEM) Fluorescence spectra were obtained with a spectrofluorometer (Jasco FP-6500) equipped with a 150W Xenon lamp. Contour maps of EEM fluorescence spectra were obtained from water extracts of whole root exudates (10 samples) or pools. The emission (Em) wavelength range was fixed from 220 to 460 nm in 5 nm steps, whereas the excitation (Ex) wavelength was fixed from 220 to 350 nm in 2 nm steps. The slit width was 5 nm and the root exudates were placed in a 1 cm path length fused quartz cell (Hellma). The UV-visible spectra of samples were acquired (SHIMADZU UV-160 spectrophotometer, 200–800 nm, 1 cm quartz cuvette). Absorbance was always lower than 0.1 (OD_units_) at 254 nm in order to reduce the absorbance of the solution to eliminate potential inner filter effects (Mobed et al., 1996). EEM fluorescence spectra of root exudates were analyzed using parallel factor analysis (PARAFAC) as in Ohno and Bro (2006). PARAFAC model Components were calculated for each treatment and time. These analyses have been performed twice with 9 or 10 biological replicates each (Experiment 1 or Experiment 2 respectively).

### Plant Hormone Analysis

Root exudate pools (Experiment 1) were lyophilized to analyze indoleacetic acid (IAA), abscisic acid (ABA), salicylic acid (SA) and jasmonic acid (JA) by ultra performance liquid chromatography-mass spectrometry (UPLC-MS). Material was extracted with 80% Methanol-1% Acetic acid. Deuterium-labeled hormones (purchased from Prof. L. Mander-Canberra, OlChemim Ltd. -Olomouc): [^2^H_5_] IAA, [^2^H_4_] SA, and [^2^H_6_] ABA were added as internal standards. For quantification of JA, dhJA was used instead. For collecting the fractions containing SA, ABA, and JA; extracts were passed consecutively through HLB (reverse phase), MCX (cationic exchange) and WAX (ionic exchange) columns (Oasis 30 mg, Waters), as described in Seo et al. (2011). The final residue was dissolved in 5% Acetonitrile – 1% Acetic acid and separated by reverse phase UPLC chromatography (2.6 μm Accucore RP-MS column, 100 mm length × 2.1 mm i.d.; Thermo Fisher Scientific) with a 5 to 50% acetonitrile gradient. Hormones were analyzed by electrospray ionization and targeted-SIM using a Q-Exactive spectrometer (Orbitrap detector, Thermo Fisher Scientific). Concentrations of hormones in extracts were determined using embedded calibration curves and the Xcalibur 4.1 SP1 build 48 and TraceFinder programs. We thank Dr. Esther Carrera for hormone quantification carried out at the Plant Hormone Quantification Service, Valencia, Spain^1^.

### Phytomelatonin Detection

Phytomelatonin was also tested in both exudates and tissues. Ten-day old seedlings were grown with chitosan (0.1, 1, 2 mg mL^–1^) in Gamborgs B5 1:10 for 3 days. Plants exposed to Gamborgs B5 1:10 only were used as control. For each treatment, 0.2–0.3 g of roots were dried onto sterile paper and placed in a 4 mL polypropylene tube with 3 mL ethyl acetate. Three biological replicates were made. Samples were shaken at 120 rpm and 4°C in the dark overnight. Roots were removed and solvent evaporated under vacuum. The dry residue was resuspended in 1 mL acetonitrile and 0.22 μm filtered. Phytomelatonin from root tissues was quantified by HPLC with fluorescence detection with Ex/Em wavelength pair of 280/348 nm, as in Hernández-Ruiz and Arnao (2008). For root exudates, three biological replicates of 1 mL of Experiment 1 pools were used. Phytomelatonin was extracted and analyzed as described above.

### Nuclear Magnetic Resonance (^1^H NMR)

Root exudates from Experiment 2 were pooled (5 replicates/pool), lyophilized and resuspended in 1 mL of D_2_O (deuterated water). Two pools were obtained from each treatment and time. Six hundred microliters of filtered pools were placed in a 5 mm NMR tube with 0.75% 3-(trimethylsilyl)propionic-2,2,3,3-d_4_ acid sodium salt (TSP) and 0.002 g sodium azide.

^1^H NMR experiments were performed on a Bruker AVIII 700 MHz (CITIUS, University of Sevilla, Spain). The number of scans was 256 and the experiments were carried out at 298 K. ^1^H chemical shifts were internally referenced to the TSP at δ 0.00. ^1^H NMR spectra were aligned using TopSpin^TM^ (Bruker).

Sensitivity of NMR is different in each region. For region I (organic-acid and amino-acid region), the threshold was set in 0.1. For II (sugars/polyalcohols region) and III (phenolics/aromatic compounds region) peaks showed less intensity, consequently the threshold was set in 0.01 and 0.001 respectively.

### High Performance Liquid Chromatography Electrospray Ionization Tandem Mass Spectrometry (HPLC-ESI-MS)

HPLC-ESI-MS analyses were performed with a High-Performance Liquid Chromatography system with an Agilent 1100 Series model coupled to a UV-visible variable wavelength detector and a mass spectrometer with ion trap analyser Series LC/MSD Trap SL (Agilent, Santa Clara, CA, United States). The mass spectrometer was operated in the positive and negative ESI modes, and the ion spray voltage was set at 4 kV. Mass range was set from 50 to 350 atomic mass units. Nitrogen was used as carrier gas (70 psi), and the ion transfer capillary heated to 350°C. Injections were carried out using an HTC Pal autosampler (CTC Analytics, Zwingen, Switzerland) equipped with a 20 μL sample loop.

Pools of 20 dap tomato root exudates (Experiment 2) were infused into the flow of the HPLC system (10 μL) through a T connection under the following conditions: flow rate, 1 mL min^–1^. Ultrapure water with 0.1% Formic Acid was used as Solvent A, whereas MeOH with 0.1% Formic Acid was used as Solvent B. From 0 to 15 min Solvent B was kept at 10% and afterward a gradient to 90% was established during 20 min and decreased again to 10% until minute 25. The LC separations were carried out with a Poroshell 120 EC-C18 column (4.6 mm × 100 mm, 2.7 μm -Agilent Technologies-). Runs were performed at 25°C. Raw data were transformed as explained in Marhuenda-Egea et al. (2013).

Partial least squares regression discriminant analyses (PLSLDA) show that *m/z* intensities depend on treatment. Loading values lower than 0 indicate that *m/z* intensity is higher in chitosan-treated plant root exudates. A threshold was set at ± 0.2 to identify *m/z* with significant variations.

Significant m/z values after PLSLDA analyses were selected for further analysis by HPLC-ESI-MS/MS using a LC-MSD-Trap-SL (Agilent). The temperature was 350°C and the pressure 70.00 psi with a flow of 12 L min^–1^. Selected masses are shown in the dataset (Supplementary Material).

After obtaining data on the fragmentation of the ions of interest, using MS\MS, we sought to identify the molecules that generated the fractionation patterns. We used two different strategies to identify the molecules. In the first strategy, we searched the databases by means of the mass of the ions, using the LC–MS search tool, using the positive (adduct type M+H) or negative (adduct type M-H) mode, with a tolerance of 0.1 Da. Once the molecules with masses similar to that searched were obtained, we checked the spectra, both real and predicted, collected in the databases that were most suitable for the MS\MS spectra we had obtained. The second strategy was based on searching different fragments generated by an ion in MS\MS fractionation using the LC–MS\MS search tool and comparing the spectra, in the same way as it was done previously. The signals from NMR and HPLC-MS were assigned, putatively, with different databases, such as Human Metabolome Database^2^, MassBank^3^ and Biological Magnetic Resonance Data Bank^4^.

### Evaluation of Tomato Root Exudates on Fungi and Nematode Eggs

Bioassays were performed in 96-well plates (Thermo Scientific) using 20 dap root exudate pools obtained in Experiment 2. These exudates were used because higher doses of chitosan would not allow the correct development of the plant and could not be used in a realistic experiment. Three technical replicates of each exudate were made. For each treatment, there were two biological replicates (pools of exudates containing 10 biological replicates initially). For fungal experiments, 200 μL of exudate (pool of 5 plant root exudates each) and conidia were added per well to reach a concentration of 10^6^ conidia⋅mL^–1^. For both, FORL and Pc, OD_490_ was calculated after 4 and 8-days respectively using a microplate reader (Tecan SPECTRAfluor). Results were handled with XFluor Software^TM^. For time series with FORL and Pc, the relation between two variables is not linear. In both cases, Smoother Model Lowess (“Locally weighted regression”) was applied (Cleveland, 1979).

To evaluate the effects of root exudates on root-knot nematode eggs, experiments were performed with 200 μl of exudate or water (control) containing 100 *M. javanica* eggs each. Hatching percentage was scored using an inverted microscope after 72 h incubation at 30°C. All experiments were performed twice with similar results.

### Statistical Analyses

Excitation emission matrix Fluorescence data was analyzed by PARAFAC, as described above, and the contribution of the Components 1, 2, and 3 analyzed by ANOVA tests. The level of significance in all cases was 95%. All statistical analyses were performed using GraphPad Prism version 7.00 (GraphPad Software, La Jolla, CA, United States^5^).

HPLC-ESI-MS data were processed using a Partial Least Square (PLS) regression model (Verboven and Hubert, 2005; Marhuenda-Egea et al., 2013). Classical PCA were also performed to display and group data (Verboven and Hubert, 2005). This data analysis was carried out using the LIBRA toolbox^6^.

## Results

### Chitosan Depolarizes Plasma Membrane and Reduces Tomato Root Cell Viability

**FIGURE 1:**
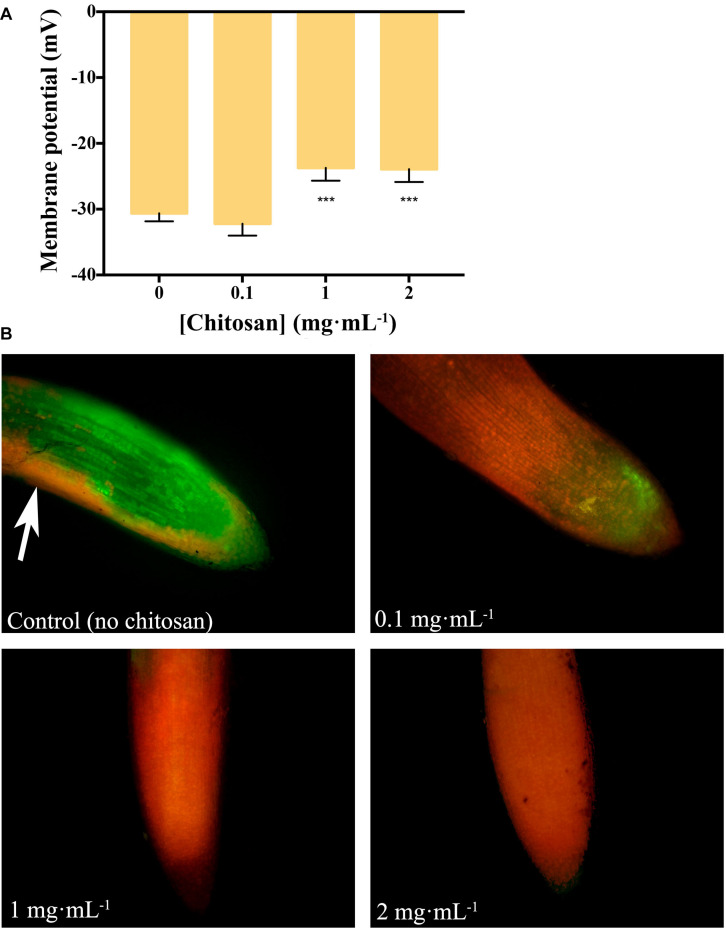
Chitosan depolarizes plasma membrane and damages tomato root cells. **(A)** Variation in membrane potential of root cells with chitosan. High doses (1 and 2 mg mL^– 1^) of chitosan significantly reduce membrane potential. **(B)** High doses of chitosan damage root cells after 24 h. Red staining labels damaged cells while green staining labels living ones. Three biological replicates were made for each treatment. Multifactorial ANOVA was used to compare treatments [*p*-values 0.05 (*), 0.01 (**), 0.001 (***), and 0.0001 (****)].

Chitosan (1 and 2 mg mL^–1^) depolarizes (*p* < 0.001) plasma membrane of tomato root cells (Figure 1A). This is reflected in loss of cell viability (PI red staining, Figure 1B). Roots incubated with 2 mg mL^–1^ chitosan show a curved morphology and dark precipitates. Conversely, a low dose of chitosan (0.1 mg mL^–1^) does not alter plasma membrane potential. However, these roots show both viable (FDA green staining) and non-viable cells, indicating some chitosan damage. Untreated tomato roots show mostly viable cells, stained with FDA. The slight red staining is due to natural senescence of root epidermic cells (Figure 1B, arrow).

### Chitosan Induces Hormones and Phenolic Compounds in Root Exudates

**FIGURE 2:**
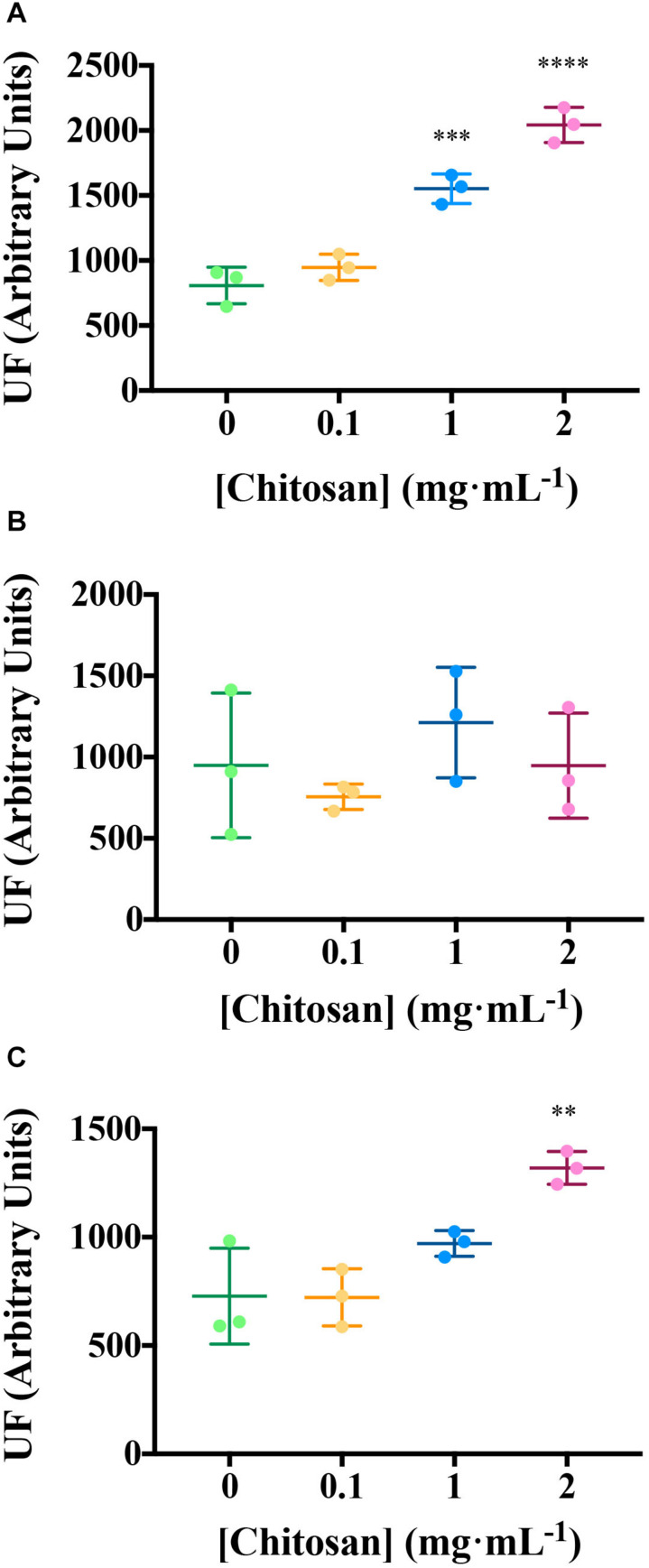
Chitosan increases EEM fluorescence of tomato root exudates. **(A)** Component 1 (salicylic acid); **(B)** Component 2 (phenolics and salicylic acid derivatives); **(C)** Component 3 (aromatic aa and peptides). For Ex/Em coordinates of Components see Table 1. UF, fluorescence units; EEM, emission excitation matrix. Mean and Standard Deviation of three biological replicates (three pools containing root exudates from three individuals each) is represented. Multifactorial ANOVA was used to compare treatments [*p*-values 0.05 (*), 0.01 (**), 0.001 (***), and 0.0001 (****)].

Excitation emission matrix Fluorescence analysis of root exudates resulted in three components (Figure 2 and Supplementary Table 1). Components 1 and 3 are the most induced (*p* < 0.01) by chitosan. Component 1 includes a putative fluorophore with Ex/Em wavelength pair of 315/430 nm, which could correspond to SA (Street and Schenk, 1981). Component 3 includes Ex/Em wavelength pairs of 245/384 and 265/384 nm, which may correspond to aromatic amino acids and peptides (Yang et al., 2015; Table 1).

**TABLE 1:** Excitation and emission pairs of coordinates for each PARAFAC model per time.

**Time**	**Excitation (nm)**	**Emission (nm)**	**Compound**
3 dap	C1: 240/315 C2: 230/280 C3: 245/265/280	C1: 430 C2: 336 C3: 384	C1: SA (Street and Schenk, 1981) C2: Phenolics (Mostofa et al., 2013; Parri et al., 2020) C3: Aromatic amino acids and peptides (Yang et al., 2015)
10 dap	C1: 240/310 C2: 285 C3: 230/275	C1: 436 C2: 364 C3: 312/358	C1: SA (Street and Schenk, 1981) C2: IAA (Li et al., 2009) C3: Aromatic amino acids and peptides (Yang et al., 2015), Phenolics (Mostofa et al., 2013; Parri et al., 2020)
20 dap	C1: 240/310 C2: 285 C3: 230/270	C1: 438 C2: 366 C3: 308	C1: SA (Street and Schenk, 1981) C2: IAA (Li et al., 2009) C3: Phenolics (Mostofa et al., 2013; Parri et al., 2020)
30 dap	C1: 235/310 C2: 230/280	C1: 440 C2: 332	C1: SA (Street and Schenk, 1981) C2: Aromatic amino acids and peptides (Yang et al., 2015), Phenolics (Mostofa et al., 2013; Parri et al., 2020)

*SA, salicylic acid; IAA, indole acetic acid; dap, days after planting; C1, component 1; C2, component 2; C3, component 3.*

**FIGURE 3:**
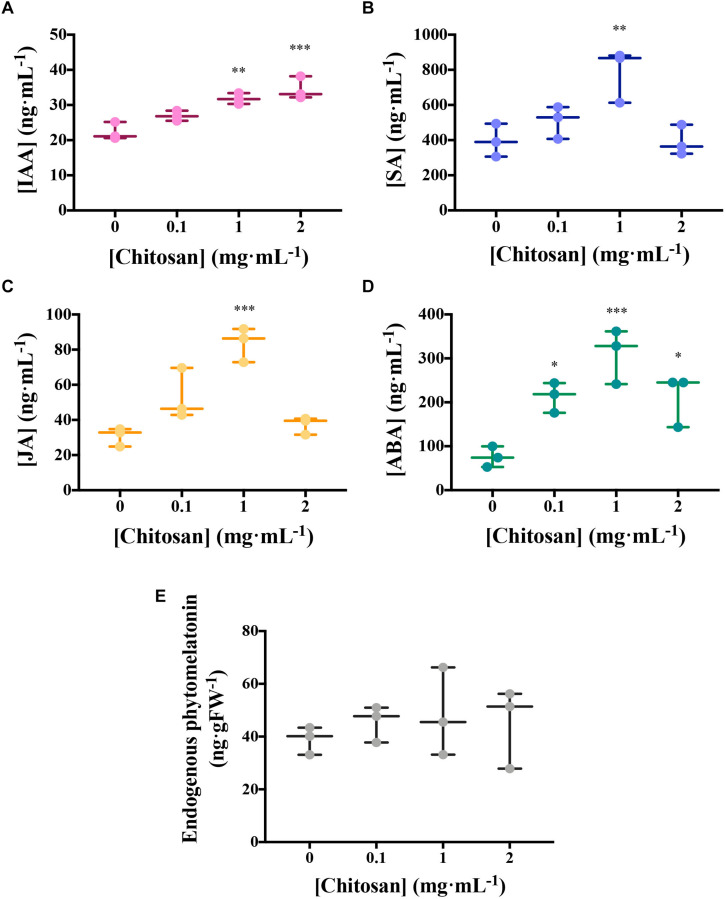
Chitosan induces plant hormones. **(A–D)** Figures correspond to hormone quantitation of accumulated tomato root exudates in three biological replicates over 3 days. **(A)** Indole acetic acid; **(B)** salicylic acid; **(C)** jasmonic acid; **(D)** abscisic acid; **(E)** Phytomelatonin quantitation in tomato roots. There is a maximum level of hormones present in exudates from plants treated with 1 mg mL^−1^ chitosan. Multifactorial ANOVA was used to compare treatments [*p*-values 0.05 (*), 0.01 (**), 0.001 (***), and 0.0001 (****)].

Chitosan induces other hormones in tomato root exudates (HPLC–MS; Figure 3). High doses of chitosan (1 and 2 mg mL^–1^) induce (*p* < 0.01) IAA accumulation in root exudates (Figure 3A). Plant defense hormones (SA, JA, and ABA) are also significantly induced (*p* < 0.05) by chitosan (1 mg mL^–1^) in tomato root exudates (Figures 3B–D). This effect is lost at 2 mg mL^–1^ chitosan. This could be the result of a root systemic damage caused by large chitosan concentrations. In view of the effect of chitosan on plant hormone homeostasis, endogenous phytomelatonin in roots was evaluated. Chitosan (1 mg mL^–1^) causes a slight rise on phytomelatonin content in tomato root cells (Figure 3E). Phytomelatonin levels detected in tomato root exudates are close to nil (Supplementary Figure 1).

### Chitosan Induces *de novo* Exudation of SA and Phenolics in Roots

**FIGURE 4:**
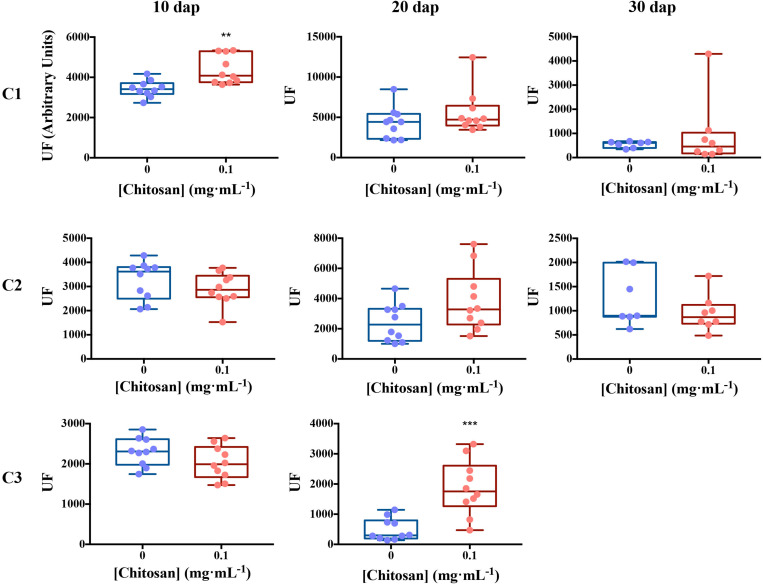
Excitation emission matrix fluorescence dynamics of tomato root exudates. For Ex/Em coordinates of Components see Table 1. Ten biological replicates per treatment were used. EEM, emission excitation matrix; dap, days after planting; C1, component 1; C2, component 2; C3, component 3; UF, fluorescence units. Mann–Whitney test was used for not normal distribution treatments. Welch’s parametric test was used for the remaining treatments [*p*-values 0.05 (*), 0.01 (**), 0.001 (***), and 0.0001 (****)].

In view of the results obtained with tomato roots elicited for 3 days with chitosan, we irrigated tomato plants with a low dose of chitosan (0.1 mgmL^–1^) during 10, 20, and 30 days and analyzed *de novo* exudation in roots. Ten days after planting (dap), chitosan increases *de novo* (*p* < 0.01) exudation of a fluorescence signature putatively belonging to SA (Figure 4 and Table 1). Twenty dap, chitosan significantly increases (*p* < 0.001) fluorescence intensity of Component 3, putatively assigned to phenolics (Mostofa et al., 2013; Parri et al., 2020) and SA derivatives (Street and Schenk, 1981). This tendency is also found for Components 1 (putatively aromatic amino acids and peptides) and 2 (putatively IAA; Li et al., 2009), although differences are not significant. In late root exudates (30 dap), fluorescence intensity decreases respect to early root exudates. This could be due to root aging and lignification. At this time, EEM Fluorescence spectrum is separated into 2 Components only (Supplementary Table 1 and Supplementary Figure 2) with no differences respect to controls.

### Metabolomic Diversity of Tomato Root Exudates Varies With Time

**FIGURE 5:**
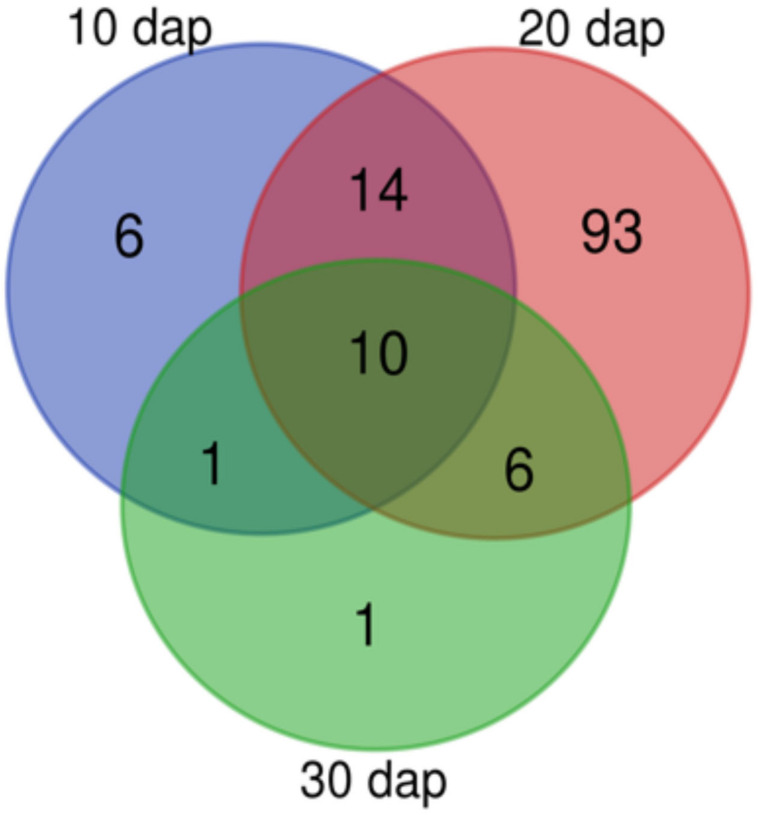
Tomato rhizodeposition varies with time. Venn-diagram of ^1^H Nuclear Magnetic Resonance analyses peaks detected in tomato root exudates at 10, 20, and 30 days after planting. In all times, plants were treated with 0.1 mg mL^– 1^ chitosan.

Pools were made to concentrate root exudates and avoid sample variability (Yuan et al., 2015; Yang et al., 2016). Root exudates from 20 dap tomato plants (chitosan treated and controls) displayed most NMR peaks (Figure 5). This may be because older plants (30 dap) are more lignified (Cervilla et al., 2009) and display less rhizodeposition. Manual curation of^ 1^H NMR profiles of root exudates from plants 10, 20, and 30 dap from chitosan treatments and controls show no qualitative differences and contain 31, 123, and 18, peaks respectively. Ninety-three peaks are specific to 20 dap root exudates. Nine 20 dap specific peaks were identified as (Table 2): leucine/isoleucine (13), acetate (53), raffinose (99, 103), glucose (100), uracil (105 and 120), cinnamic acid (109, 119, 121), fumaric acid (109), *p*-aminobenzoic acid (112 and 122) and trigonelline (125, 130, 131). Chitosan decreases (*p* < 0.05) acetate (peak 53) content in 20 dap root exudates. Six peaks, among them methanol (80) and formic acid (129), are found 20 and 30 dap. Lactate (peak 36) and malate (peaks 62 and 64) have been detected in all time points evaluated. All detected peaks are listed in Supplementary Table 2. A representative ^1^H NMR profile of tomato root exudates is shown in Supplementary Figure 3.

**TABLE 2:** Peak assignments for ^1^H NMR spectra of tomato root exudates.

						**20 dap [Chitosan] (mg⋅mL^–1^)**	
**Part of the spectra**	**dap**	**Peak number**	**Compound**	**Shift (ppm)**	**H mult.**	**0**	**0.1**
I	20	13	Leucine/isoleucine	0.94		0.25475	0.17575
I	10-20-30	36	Lactate	1.33	d	0.35685	0.18225
I	20	53	Acetate	1.92	s	4.7895	**3.4135**
I	10-20-30	62	Malate	2.3	t	0.6217	0.5769
I	10-20-30	64	Malate	2.41		1.725	1.26
II	20-30	80	Methanol	3.36	s	0.34845	0.2379
II	20	99	Raffinose	4.99	d	0.027535	0.03025
II	20	100	Glucose	5.237	d	0.02523	0.035445
II	20	103	Raffinose	5.42	d	0.02462	0.02018
II	20	105	Uracil	5.79	d	0.031275	0.02049
III	20	109	Cinnamic acid/fumaric acid	6.52/6.51	d/s	0.2005	0.15425
III	20	112	*p*-Aminobenzoic acid	6.82	d	0.0033845	0.0050605
III	20	119	Cinnamic acid	7.43	m	0.049535	0.04216
III	20	120	Uracil	7.52	d	0.00791	0.016235
III	20	121	Cinnamic acid	7.62	dt	0	0.002784
III	20	122	*p*-Aminobenzoic acid	7.73	d	0.0056825	0.00603
III	20	125	Trigonelline	8.07	m	0.006835	0.01239
III	20-30	129	Formic acid	8.44	m	0.35505	0.1718
III	20	130	Trigonelline	8.82	m	0.028845	0.022265
III	20	131	Trigonelline	9.13	s	0.03144	0.024555

*This table includes identified peaks only. Spectra parts I, II, and III are indicated in Supplementary Figure 3. Abbreviations: Dap, days after planting; H mult., H multiplicity; d, doublet; s, singlet; t, triplet; dt, double of triplets; m, multiplet. Treatment abbreviations: 0, Tomato Root Exudates Control; 0.1, Tomato Root Exudates from plants irrigated with 0.1 mg mL^–1^ chitosan. Numbers in treatments correspond to the mean of maximum intensity of the peaks (arbitrary units). Bold numbers indicate significant differences (ANOVA, *p* < 0.05).*

### Chitosan Induces Lipid Signaling and Defense Compounds in Tomato Root Exudates

**FIGURE 6:**
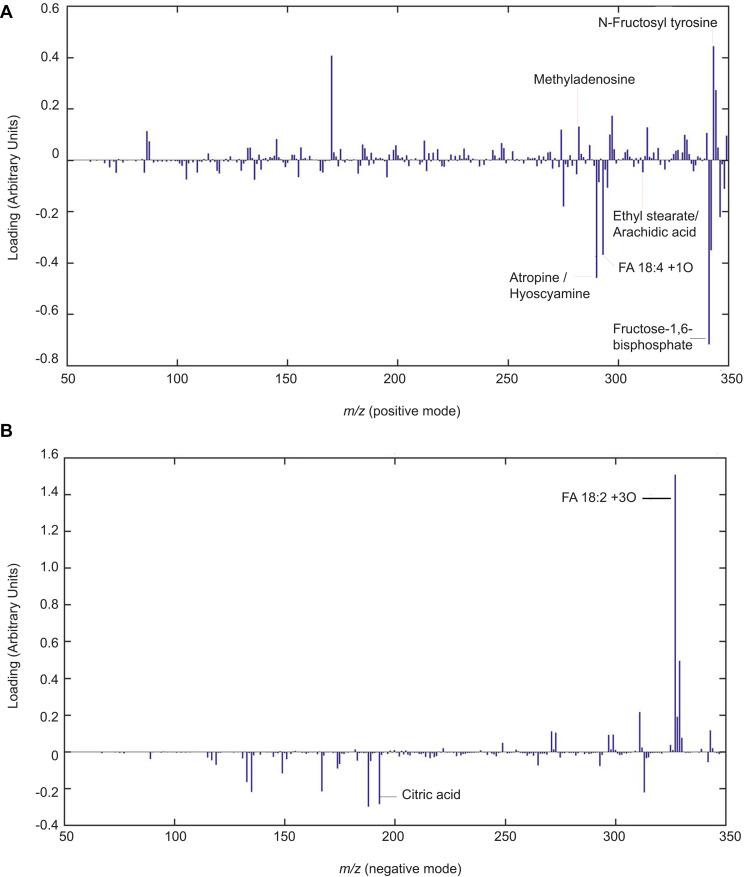
Chitosan induces lipid signaling and defense compounds in tomato root exudates. Partial least squares regression discriminant analysis (PLSLDA) of HPLC-ESI-MS analysis of pools of 20 days after planting tomato root exudates in modes positive **(A)** and negative **(B)**. Four biological replicates per treatment are represented. Blue bars indicate a particular mass that differs from the control treatment. The larger the bar size, the more noticeable the difference in intensity of the mass compared to the control.

Lipid signaling and defense compounds are putatively identified in 20 dap root exudates (Figure 6 and Supplementary Table 4, Supplementary Database HPLC-MS/MS). Putative oxidized fatty acid (FA18:4+1O, *m/z* 293.5^+^) and other fatty acids (Ethyl stearate/Arachidic acid, *m/z* 313.7^+^) are increased with chitosan. On the contrary, FA18:2+3O (*m/z* 327.3^–^) is reduced with chitosan (*m/z* 290.5^+^). Stress response metabolites such as -putatively- Atropine / Hyoscyamine (A/H, *m/z* 290.5^+^) and putative citric acid (CA, *m/z* 191.0^–^) are increased with chitosan. On the contrary, 3-*O*′-Methyladenosine (3OM, *m/z* 282.7^+^), is slightly reduced. *N*-Fructosyl tyrosine (*m/z* 344.3^+^) is slightly decreased with chitosan. Putative Fructose-1,6-bisphosphate (FB, *m/z* 327.7^–^), involved in the glycolysis pathway, is increased with chitosan.

### Root Exudates From Plants Treated With Chitosan Inhibit Soil-Borne Pathogens

**FIGURE 7:**
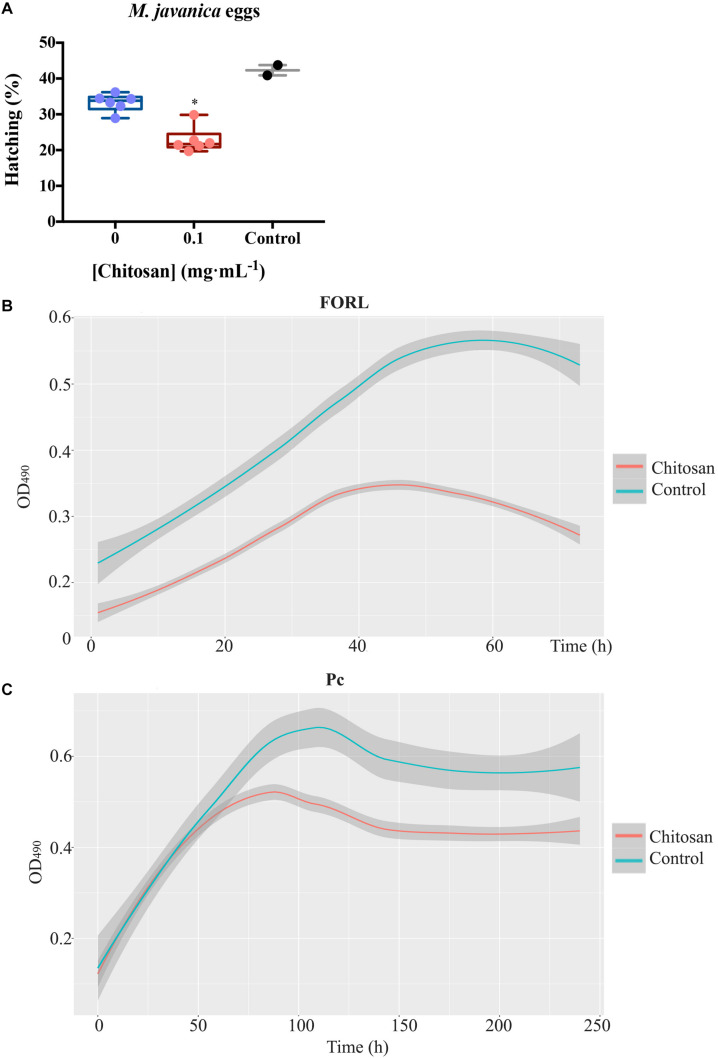
Root exudates from plants treated with chitosan inhibit soil-borne pathogens. **(A)** Effect on hatching of *Meloidogyne javanica* eggs in tomato root exudates. Egg hatching is reduced ca. 1.5-fold. Multifactorial analysis Kruskal–Wallis was performed to compare treatments [*p*-values 0.05 (*), 0.01 (**), 0.001 (***), and 0.0001 (****)] Control is egg hatching in water, 0 corresponds to untreated tomato root exudates. **(B)** Growth kinetics of FORL in root exudates, chitosan inhibits ca. twofold growth. C, Growth kinetics of *P. chlamydosporia* strain 123 in tomato root exudates. Smoother Models were adjusted with band 0.75 and degree 2. Shadow area stablishes the prediction confidence intervals at 95%.

Soil-borne pathogens (fungi and nematodes) are inhibited by root exudates from plants treated with chitosan. Root exudates from chitosan-treated plants cause ca. 1.5-fold reduction (*p* < 0.05) on hatching of the root-knot nematode *Meloydogine javanica* eggs after 72 h respect to tomato control root exudates (Figure 7A). These exudates also inhibit growth of *Fusarium oxysporum* f.sp. radicis-lycopersici (FORL) ca. twofold respect to controls (Figure 7B). The chitosan resistant fungus *Pochonia chlamydosporia* strain 123 (Pc) does not show significant differences in growth with both exudates over time (Figure 7C).

## Discussion

Chitosan has a well stablished role as a plant defense activator (El Hadrami et al., 2010). In this study we give evidences that chitosan induces plant defenses in tomato root exudates. Plasma membranes are likely to be a main target of this molecule (Palma-Guerrero et al., 2010; Meisrimler et al., 2011; Jaime et al., 2012). In this work, we show that increasing chitosan concentrations depolarize tomato root cell plasma membrane. He et al. (2009) proposed chitosan as a potential cell penetration enhancer. This fact has been demonstrated using vital staining in fungi (Palma-Guerrero et al., 2010) and, in this work, in root apices. These effects of chitosan on roots are likely to modify rhizodeposition (Pitta-Alvarez and Giulietti, 1999). Chitosan membrane depolarization may trigger an increase of reactive oxygen species (ROS) in the plant cell, generating secondary metabolites (Pandey, 2017). ROS are known to alter chemical components of the cell and lipids in particular. We have found putative FA18:4+1O and Ethyl stearate/Arachidic acid increase in exudates from roots treated with chitosan. These fatty acids could have been released from cell membranes by the oxidative stress produced by chitosan in plants (Lopez-Moya et al., 2017). These long chain fatty acids could be involved in the production of oxylipins (Noverr et al., 2003). Some oxylipins, such as oxophytodienoic acid (OPDA), are JA precursors (Dave and Graham, 2012). This could explain our finding of JA increase in root exudates from chitosan treated plants. Oxylipins other than jasmonates are probably also essential for the resistance of plants to pathogens (Blée, 2002). Putative alkaloids and CA are also increased with chitosan. These molecules have been shown to mediate stress in plant cell responses (Moharrami et al., 2017; Mallhi et al., 2019). The increase of fructose-1,6-bisphosphate in root exudates from plants treated with chitosan could reflect an extra consumption of glucose in root cells in response to chitosan induced ROS increase (Cao et al., 2020). Finally, the slight reduction of Methyladenosine in root exudates from chitosan-treated plants, could indicate that this polymer is perhaps affecting gene expression in roots (Zheng et al., 2020).

Importance of chitosan in plant hormone production and systemic acquired resistance has been widely demonstrated (Mika et al., 2010; Colman et al., 2019; Fooladi vanda et al., 2019; Iglesias et al., 2019; Ma et al., 2019). We have found that increasing chitosan leads to the accumulation of fluorescence compounds corresponding to phenolics, as well as hormones related to plant growth and defense (JA, SA, ABA, and IAA). This correlation between chitosan and phenolics has been previously studied (Pitta-Alvarez and Giulietti, 1999; Park et al., 2019; Jaisi and Panichayupakaranant, 2020; Samari et al., 2020). Chitosan enhances metabolic pathways (e.g., phenylpropanoid) involved in the biosynthesis of phenolic compounds (Asgari-Targhi et al., 2018; Fooladi vanda et al., 2019; Singh et al., 2020). A low dose of chitosan enhances plant immunity via plant hormone (JA and SA, mainly) accumulation in root tissues (Lopez-Moya et al., 2017; Iglesias et al., 2019; Singh et al., 2020). However, this effect has not yet been detected in tomato root exudates. In addition, there is evidence that chitosan could be used as a substitute for commonly used growth factors such as methyl jasmonate, auxins, or cytokinins (Cui et al., 2012; Sivanandhan et al., 2012; Ahmad et al., 2019; Acemi, 2020) due to its elicitor effects (Salachna and Zawadzińska, 2014; Malerba and Cerana, 2016). Phytomelatonin has been recently considered a plant master regulator involved in abiotic and biotic stress responses. In our work, exposure of tomato roots to increasing chitosan doses accumulates phytomelatonin and hormones in roots and root exudates in a typical biotic stress response (Arnao and Hernández-Ruiz, 2019; Moustafa-Farag et al., 2019). The decrease in levels of some plant hormones at toxic chitosan doses has also been found for phenolics (Park et al., 2019). EEM Fluorescence signatures corresponding to IAA (Li et al., 2009) and SA (Miles and Schenk, 1970) are increased by chitosan in tomato root exudates. These hormones are known to be increased by chitosan (Lopez-Moya et al., 2017; Fooladi vanda et al., 2019). SA and JA are related to plant responses to stress (War et al., 2011). Therefore, chitosan can be an elicitor of plant defenses (Benhamou, 1996) in root exudates. Our bioassays show that chitosan induces root exudates inhibitory to root pathogenic fungus FORL and root-knot nematode eggs without significantly affecting the growth and development of a biocontrol fungus (Pc). The toxic effect of chitosan-derived exudates may be related to the overproduction of SA which, in combination with chitosan induce systemic acquired resistance and reduce infection by root-knot nematodes (Vasyukova et al., 2003; Singh et al., 2020). Chitosan, moreover, by itself, is toxic to fungi such as *Fusarium* spp. (Palma-Guerrero et al., 2010; Al-Hetar et al., 2011). Other studies show that plants treated with chitosan are protected from FORL infection (Benhamou and Thériault, 1992).

Chitosan depolarizes plasma membrane of tomato root cells, causing the secretion of hormones, lipid signaling and plant defense compounds, including phenolics. This process affects cell viability and rhizodeposition, so plant age must be considered before applying chitosan, as well as the duration of applications. Further studies should test chitosan derived exudates under *in vivo* conditions to determine their efficacy to prevent the proliferation of root pathogens and subsequently reduce disease severity. Overall, our results have proven that root exudates of chitosan-treated plants are capable to reduce soil-borne pathogens growth by *in vitro*. This makes chitosan a promising agent to prevent and manage pests and diseases in a sustainable way.

## Data Availability Statement

The datasets presented in this study can be found in online repositories. The names of the repository/repositories and accession number(s) can be found below: https://datadryad.org/stash/share/iQ4Fh9XTzpF5Rbsgz9dakjCP2BfvpSigHxhhuy57GY.

## Author Contributions

LVL-L conceived the original screening and research plans and wrote the manuscript. MS-F performed the main experiments and wrote the manuscript. FCM-E analyzed the fluorescence, 1H NMR and HPLC-MS data. FL-M supervised the experiments and writing. MBA provided technical assistance to phytomelatonin detection experiments. FC-E provided technical assistance to 1H NMR experiments. MJN performed the time series analyses. BG performed electrophysiological and vital staining experiments. MS-F agrees to serve as the author responsible for contact and ensures communication. All authors contributed to the article and approved the submitted version.

## Conflict of Interest

The authors declare that the research was conducted in the absence of any commercial or financial relationships that could be construed as a potential conflict of interest.

## Footnotes

1 www.ibmcp.upv.es

2 https://hmdb.ca/

3 https://massbank.eu/MassBank/

4 http://www.bmrb.wisc.edu/metabolomics/

5 https://www.graphpad.com/

6 http://wis.kuleuven.be/stat/robust/software
