# Supplementary material for: Chitosan Induces Plant Hormones and Defenses in Tomato Root Exudates
Source: Front Plant Sci. 2020 Nov 4;11:572087. doi: 10.3389/fpls.2020.572087 (PMC7672008; doi:10.3389/fpls.2020.572087)
Supplement: Supplementary file 1 [file Data_Sheet_1.pdf]

Display Report - Selected Window Selected

Analysis Name: MSU066.D
Method: GENERA~3.M
Sample Na TPQ-1-20
Analysis Inf

Instrume LC-MSD-Trap-SL
Operator: Pablo

Print Da 03/07/2 01:05:02 019 PM
Acq. Dat 02/25/2019 05:04:43 PM

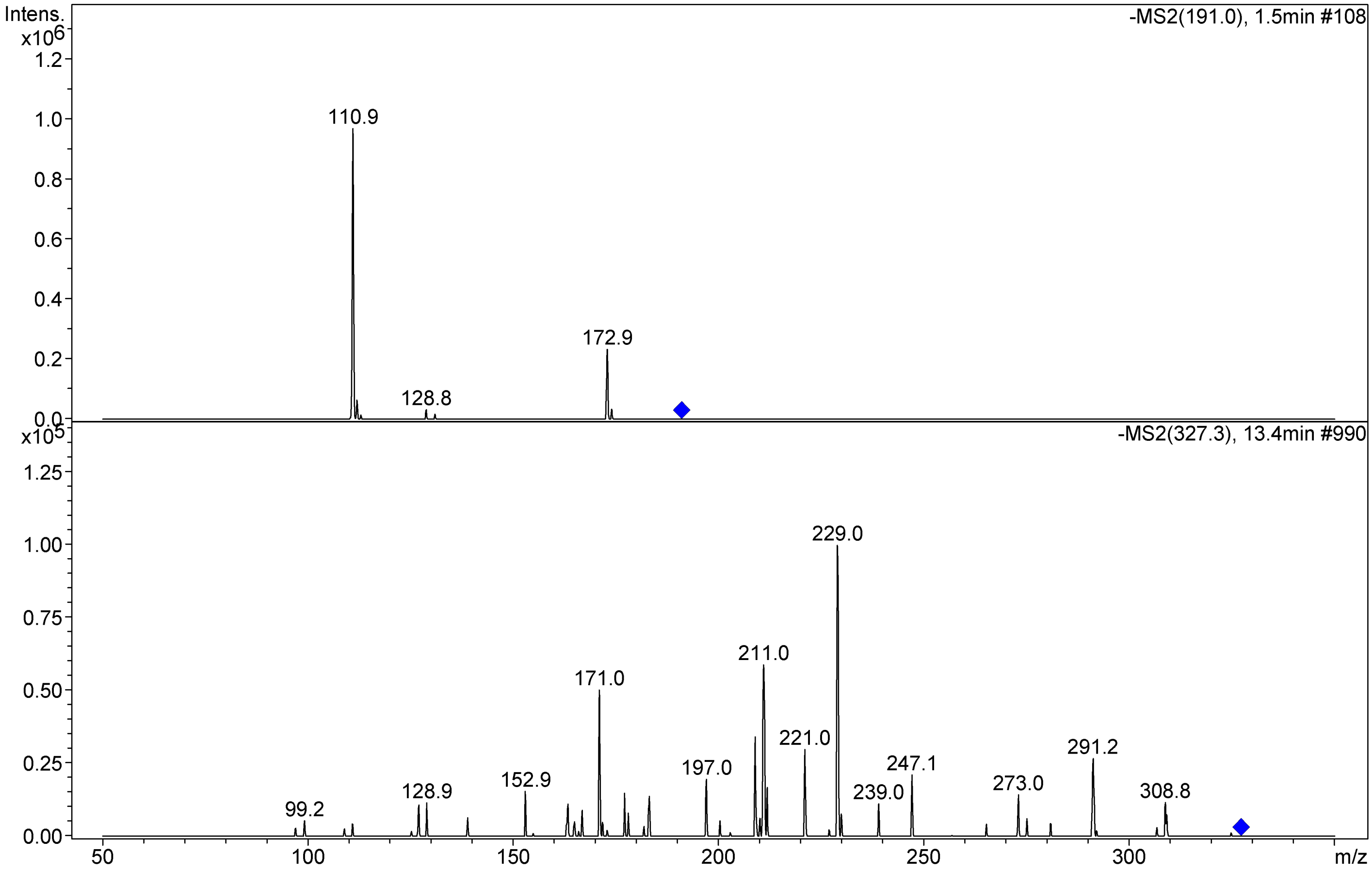

Agilent Technologies

Compound Mass Spectrum List Report - MS

Analysis Name: MSU066.D **Instrument:** LC-MSD-Trap-SL **Print Date:** 03/07/2019 01:14:29 PM
Method: GENERA~3.M **Operator:** Pablo **Acq. Date:** 02/25/2019 05:04:43 PM
Sample Name: TPQ-1-20
Analysis Info:

Acquisition Parameter:

Mass Range Mode	Std/Normal	Trap Drive	35.9	Scan Begin	50 m/z
Ion Polarity	Negative	Octopole RF Amplitude	120.0 Vpp	Scan End	350 m/z
Ion Source Type	ESI	Capillary Exit	-106.0 Volt	Averages	4 Spectra
Dry Temp (Set)	350 °C	Skimmer	-40.0 Volt	Max. Accu Time	200000 µs
Nebulizer (Set)	70.00 psi	Oct 1 DC	-12.00 Volt	ICC Target	30000
Dry Gas (Set)	12.00 l/min	Oct 2 DC	-1.70 Volt	Charge Control	on

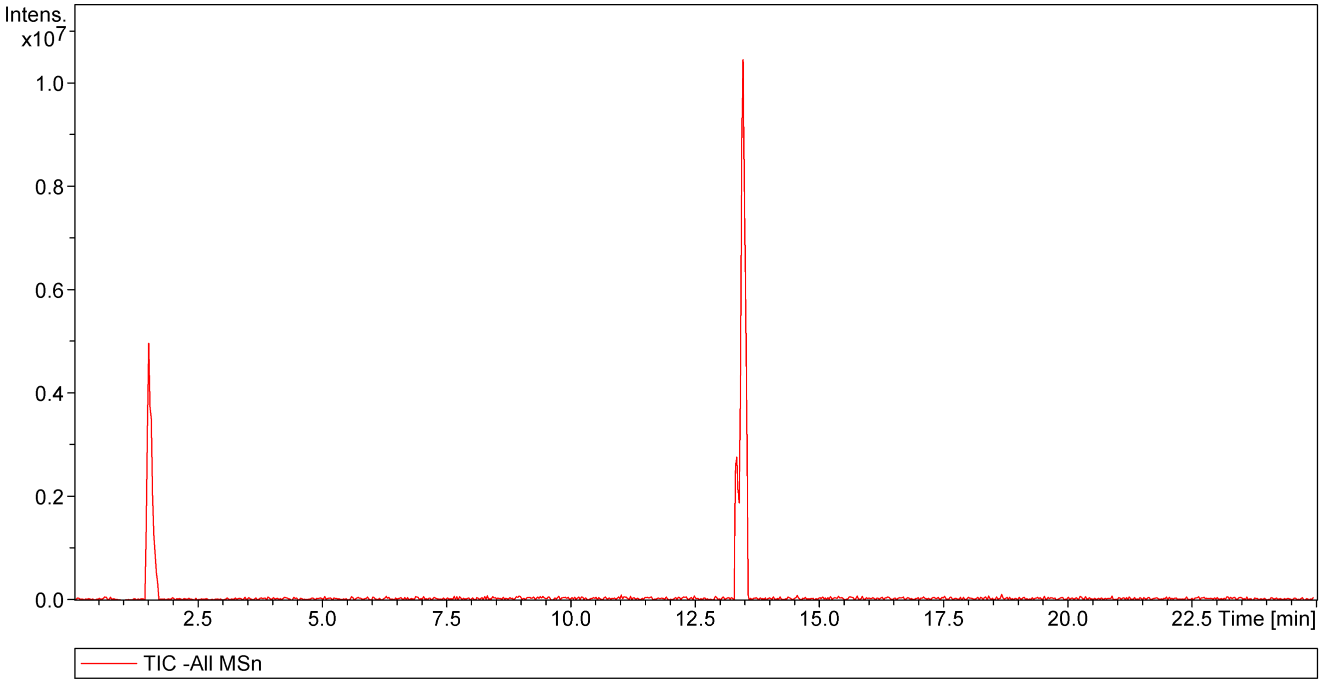

Compound List:

#	RT [min]	Range [min]	Height	Area	Area Frac %
n.a.	1.5	1.5	n.a.	n.a.	n.a.
n.a.	13.4	13.4	n.a.	n.a.	n.a.

Compound Mass Spectrum List Report - MS

-MS2(191.0), 1.5min #108

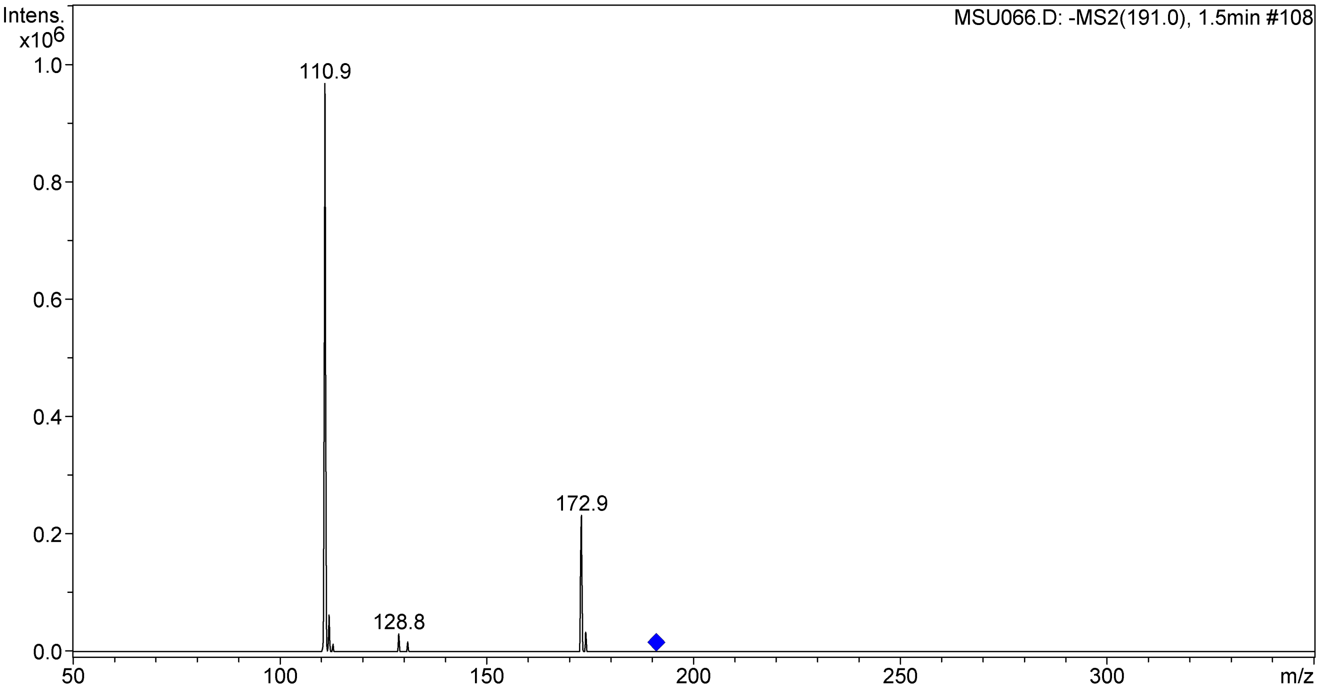

Mass List:

#	m/z	Res.	FWHM	I	I %	S/N
1	110.9	307	0.4	967296	100.0	69.1
2	111.9	409	0.3	62345	6.4	4.5
3	128.8	501	0.3	30177	3.1	2.2
4	130.9	560	0.2	16532	1.7	1.2
5	172.9	466	0.4	231759	24.0	16.6
6	173.9	681	0.3	32944	3.4	2.4

Compound Mass Spectrum List Report - MS

-MS2(327.3), 13.4min #990

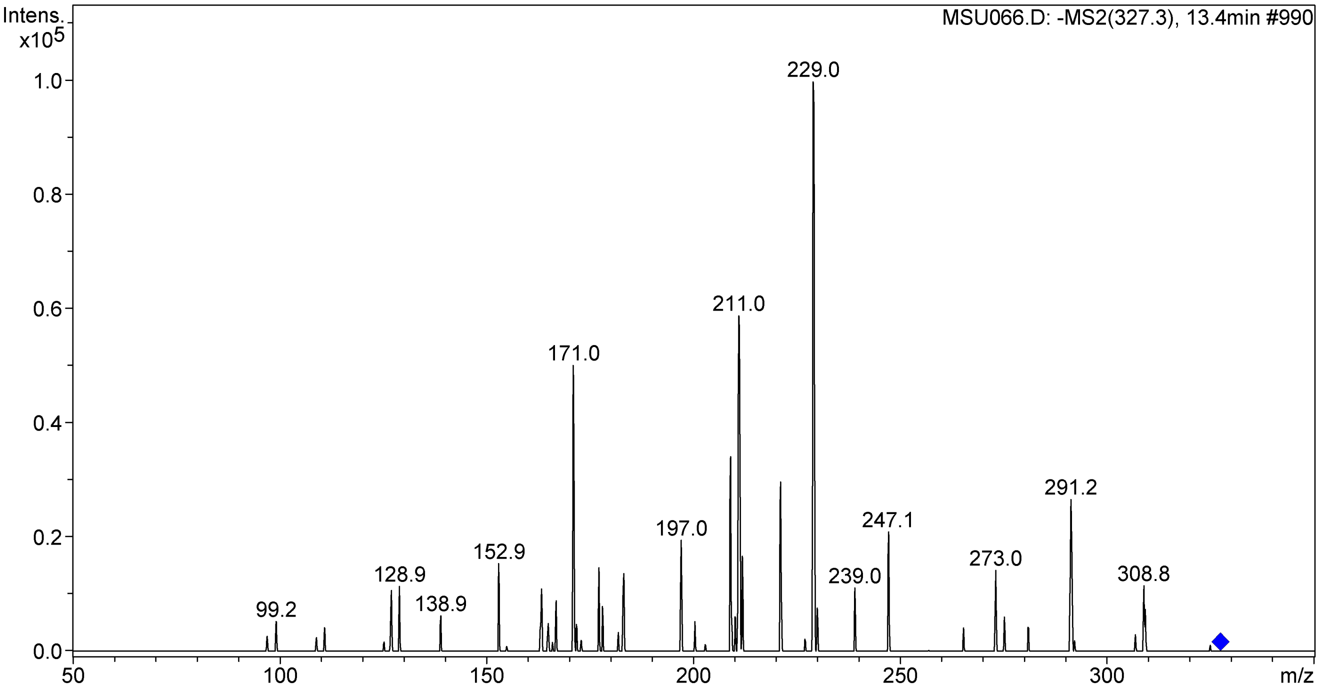

Mass List:

#	m/z	Res.	FWHM	I	I %	S/N
1	99.2	380	0.3	5182	5.2	1.1
2	127.0	451	0.3	10625	10.7	2.4
3	128.9	576	0.2	11315	11.4	2.5
4	138.9	553	0.3	6204	6.2	1.4
5	152.9	660	0.2	15305	15.4	3.4
6	163.3	529	0.3	10886	10.9	2.4
7	164.9	437	0.4	4834	4.9	1.1
8	166.8	644	0.3	8823	8.9	2.0
9	171.0	551	0.3	50005	50.2	11.1
10	171.7	707	0.2	4668	4.7	1.0
11	177.1	797	0.2	14596	14.6	3.2
12	178.0	804	0.2	7821	7.8	1.7
13	183.1	451	0.4	13568	13.6	3.0
14	197.0	622	0.3	19428	19.5	4.3
15	200.3	876	0.2	5167	5.2	1.1
16	208.9	749	0.3	34015	34.1	7.5
17	210.1	811	0.3	6004	6.0	1.3
18	211.0	416	0.5	58690	58.9	13.0
19	211.8	867	0.2	16608	16.7	3.7
20	221.0	710	0.3	29622	29.7	6.6
21	229.0	514	0.4	99643	100.0	22.1
22	229.9	821	0.3	7528	7.6	1.7
23	239.0	933	0.3	11036	11.1	2.4
24	247.1	847	0.3	20866	20.9	4.6
25	273.0	967	0.3	14152	14.2	3.1

Compound Mass Spectrum List Report - MS

#	m/z	Res.	FWHM	I	I %	S/N
26	275.1	1110	0.2	5954	6.0	1.3
27	291.2	634	0.5	26532	26.6	5.9
28	308.8	1363	0.2	11432	11.5	2.5
29	309.1	1545	0.2	7272	7.3	1.6

Display Report - Selected Window Selected

Analysis Name: MSU067.D
Method: GENERA~3.M
Sample Na TPQ-2-20
Analysis Inf

Instrume LC-MSD-Trap-SL
Operator: Pablo

Print Da 03/07/2 019 01:06:33 PM
Acq. Dat 02/25/2019 05:32:47 PM

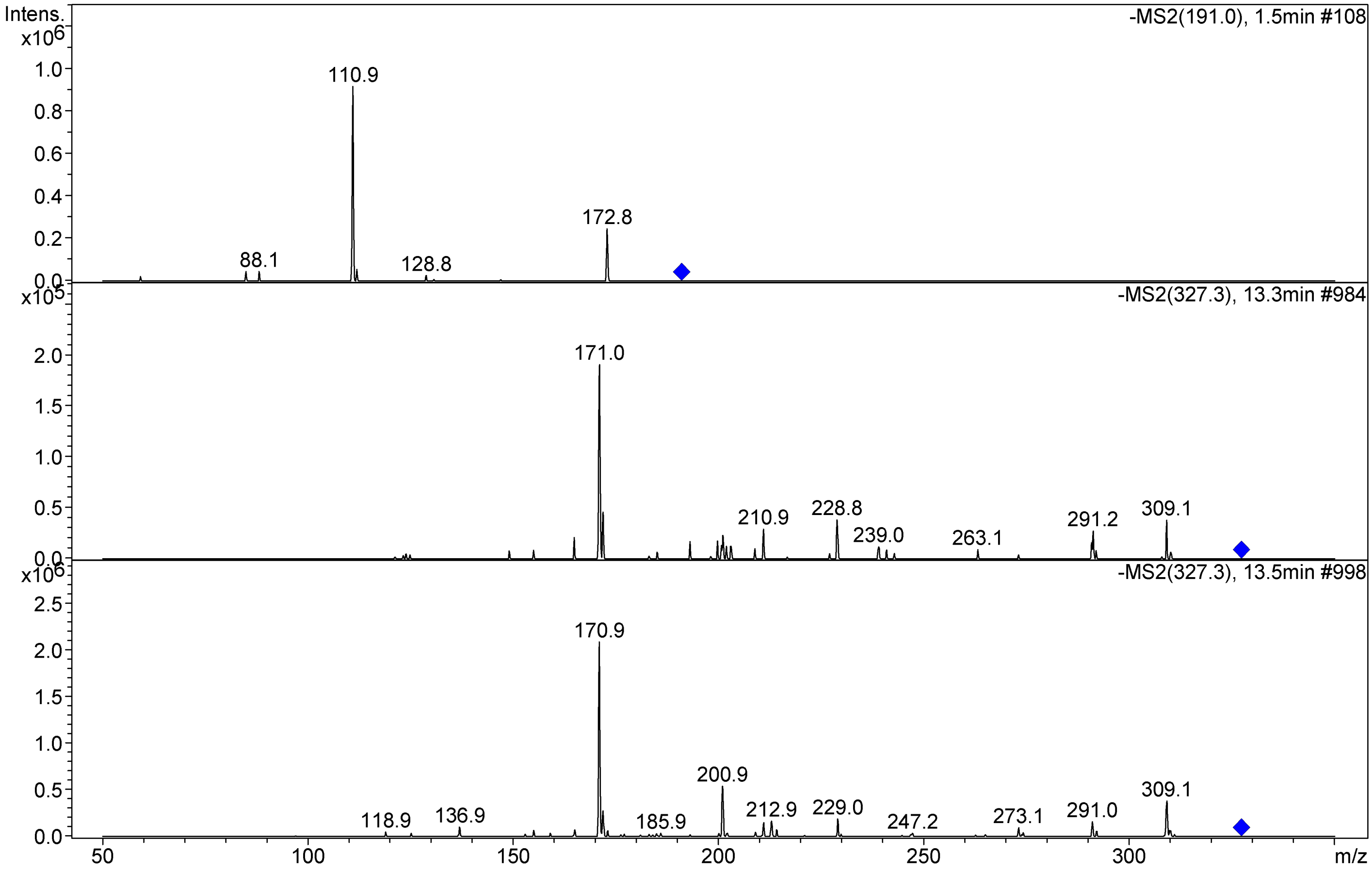

Agilent Technologies

Compound Mass Spectrum List Report - MS

Analysis Name: MSU067.D **Instrument:** LC-MSD-Trap-SL **Print Date:** 03/07/2019 03:33:38 PM
Method: GENERA~3.M **Operator:** Pablo **Acq. Date:** 02/25/2019 05:32:47 PM
Sample Name: TPQ-2-20
Analysis Info:

Acquisition Parameter:

Mass Range Mode	Std/Normal	Trap Drive	35.9	Scan Begin	50 m/z
Ion Polarity	Negative	Octopole RF Amplitude	120.0 Vpp	Scan End	350 m/z
Ion Source Type	ESI	Capillary Exit	-106.0 Volt	Averages	4 Spectra
Dry Temp (Set)	350 °C	Skimmer	-40.0 Volt	Max. Accu Time	200000 µs
Nebulizer (Set)	70.00 psi	Oct 1 DC	-12.00 Volt	ICC Target	30000
Dry Gas (Set)	12.00 l/min	Oct 2 DC	-1.70 Volt	Charge Control	on

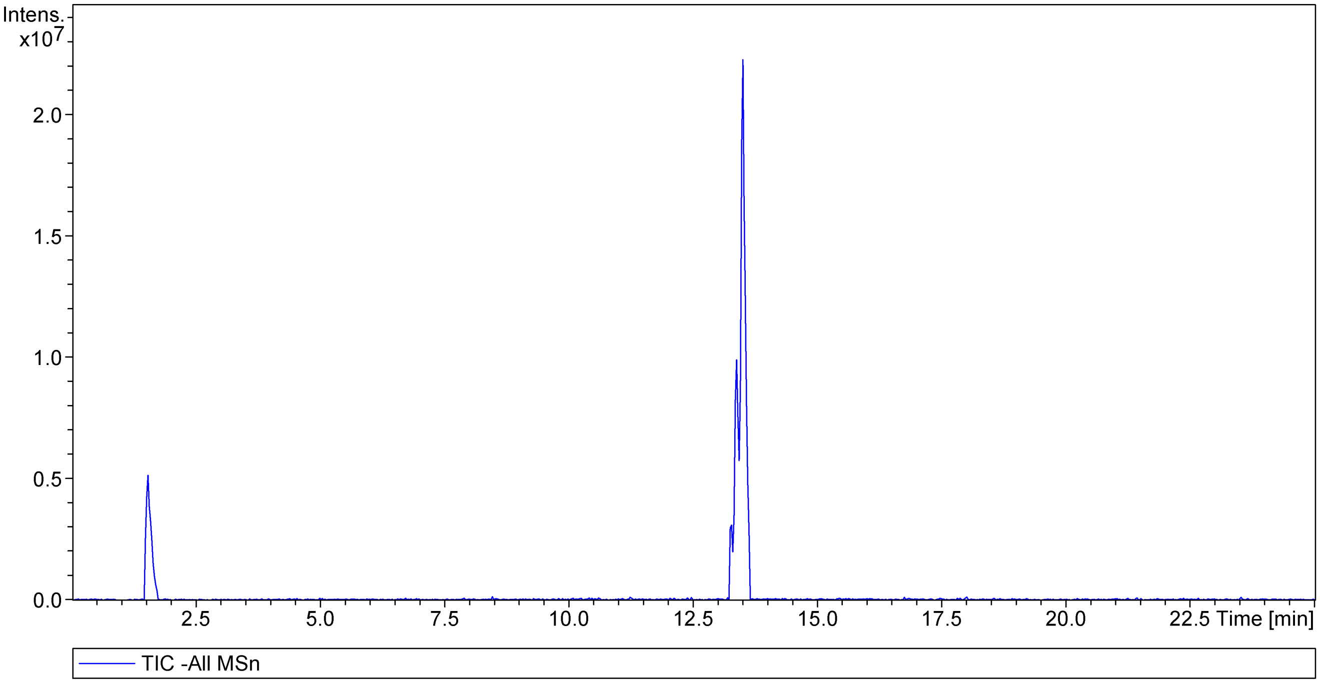

Compound List:

#	RT [min]	Range [min]	Height	Area	Area Frac %
n.a.	1.5	1.5	n.a.	n.a.	n.a.
n.a.	13.3	13.3	n.a.	n.a.	n.a.
n.a.	13.5	13.5	n.a.	n.a.	n.a.

Compound Mass Spectrum List Report - MS

-MS2(191.0), 1.5min #108

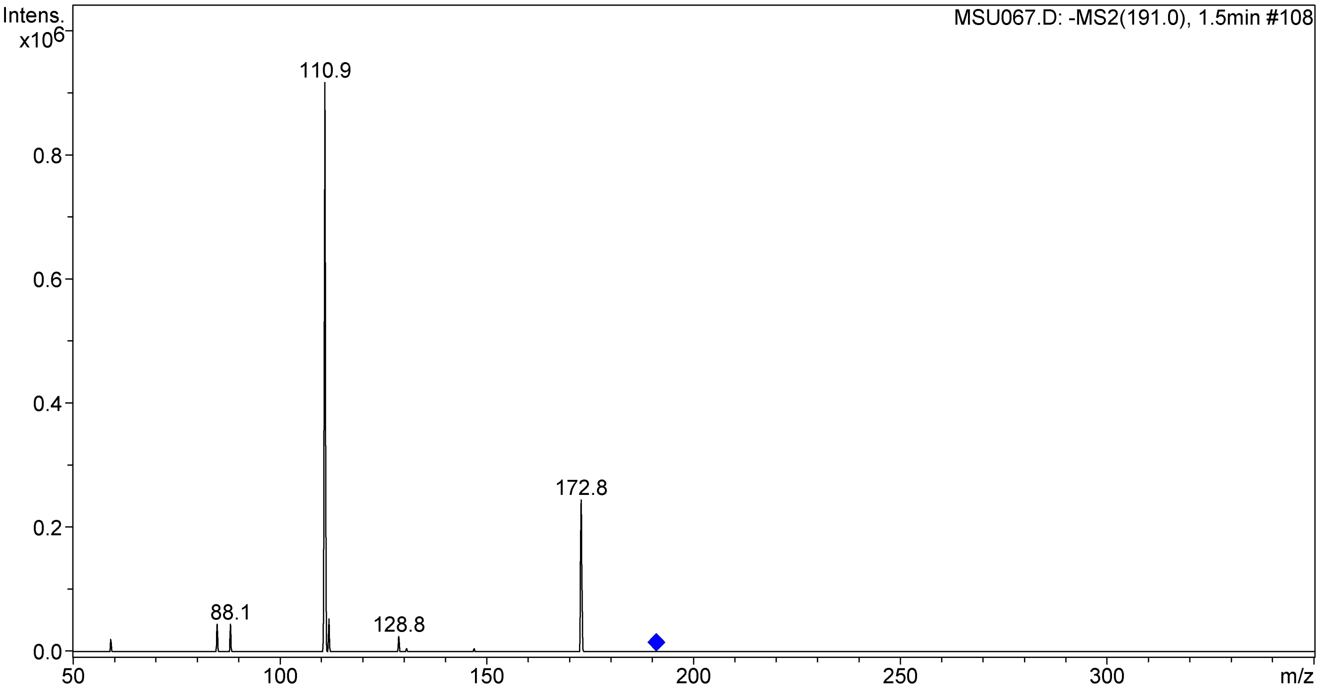

Mass List:

#	m/z	Res.	FWHM	I	I %	S/N
1	84.9	339	0.3	43780	4.8	2.2
2	88.1	391	0.2	44158	4.8	2.2
3	110.9	329	0.3	916151	100.0	45.9
4	111.9	433	0.3	52955	5.8	2.7
5	128.8	508	0.3	24288	2.7	1.2
6	172.8	499	0.3	244337	26.7	12.2

Compound Mass Spectrum List Report - MS

-MS2(327.3), 13.3min #984

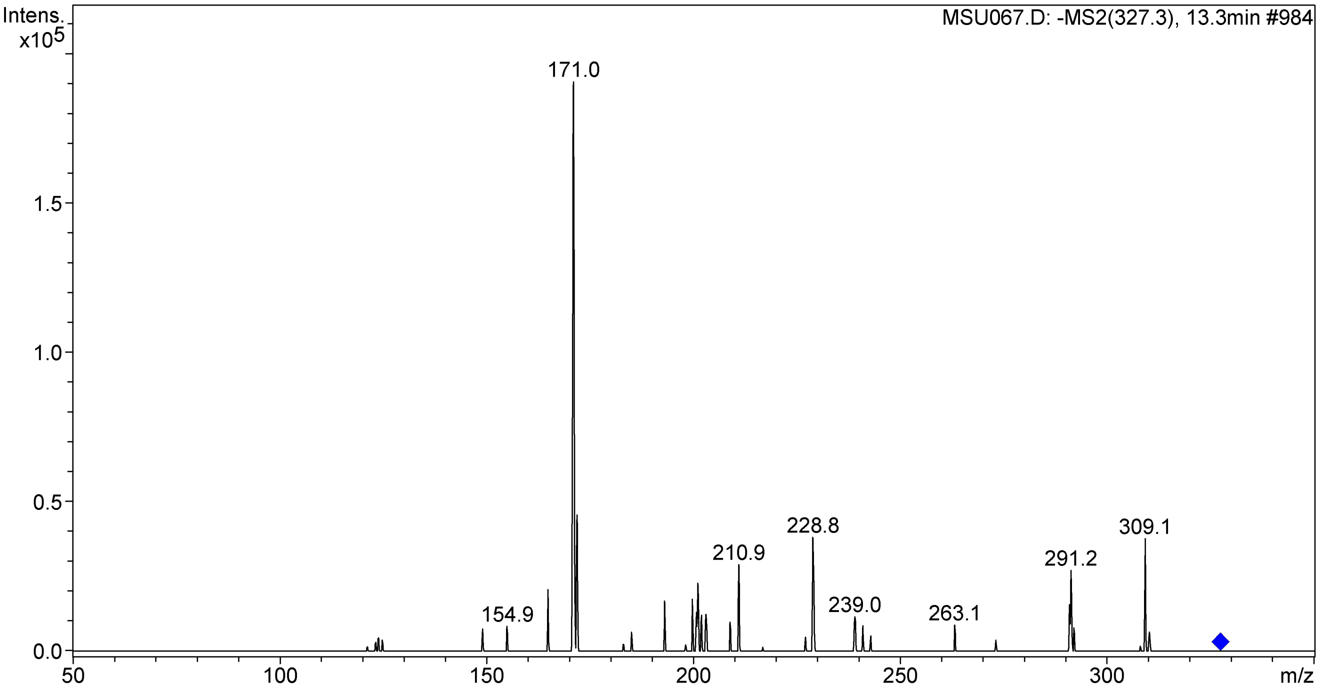

Mass List:

#	m/z	Res.	FWHM	I	I %	S/N
1	149.0	658	0.2	7381	3.9	1.5
2	154.9	671	0.2	8228	4.3	1.7
3	164.8	729	0.2	20565	10.8	4.2
4	171.0	446	0.4	190455	100.0	38.5
5	171.8	545	0.3	45514	23.9	9.2
6	185.0	772	0.2	6344	3.3	1.3
7	193.0	856	0.2	16684	8.8	3.4
8	199.7	867	0.2	17369	9.1	3.5
9	200.7	996	0.2	13024	6.8	2.6
10	201.0	755	0.3	22681	11.9	4.6
11	201.9	785	0.3	12016	6.3	2.4
12	203.0	520	0.4	12193	6.4	2.5
13	208.8	881	0.2	9636	5.1	1.9
14	210.9	743	0.3	28939	15.2	5.9
15	228.8	604	0.4	38060	20.0	7.7
16	239.0	551	0.4	11501	6.0	2.3
17	240.9	965	0.2	8487	4.5	1.7
18	242.8	974	0.2	5006	2.6	1.0
19	263.1	1163	0.2	8730	4.6	1.8
20	290.8	1498	0.2	15586	8.2	3.2
21	291.2	1157	0.3	27041	14.2	5.5
22	291.9	1263	0.2	7746	4.1	1.6
23	309.1	1240	0.2	37637	19.8	7.6
24	310.1	840	0.4	6294	3.3	1.3

Compound Mass Spectrum List Report - MS

-MS2(327.3), 13.5min #998

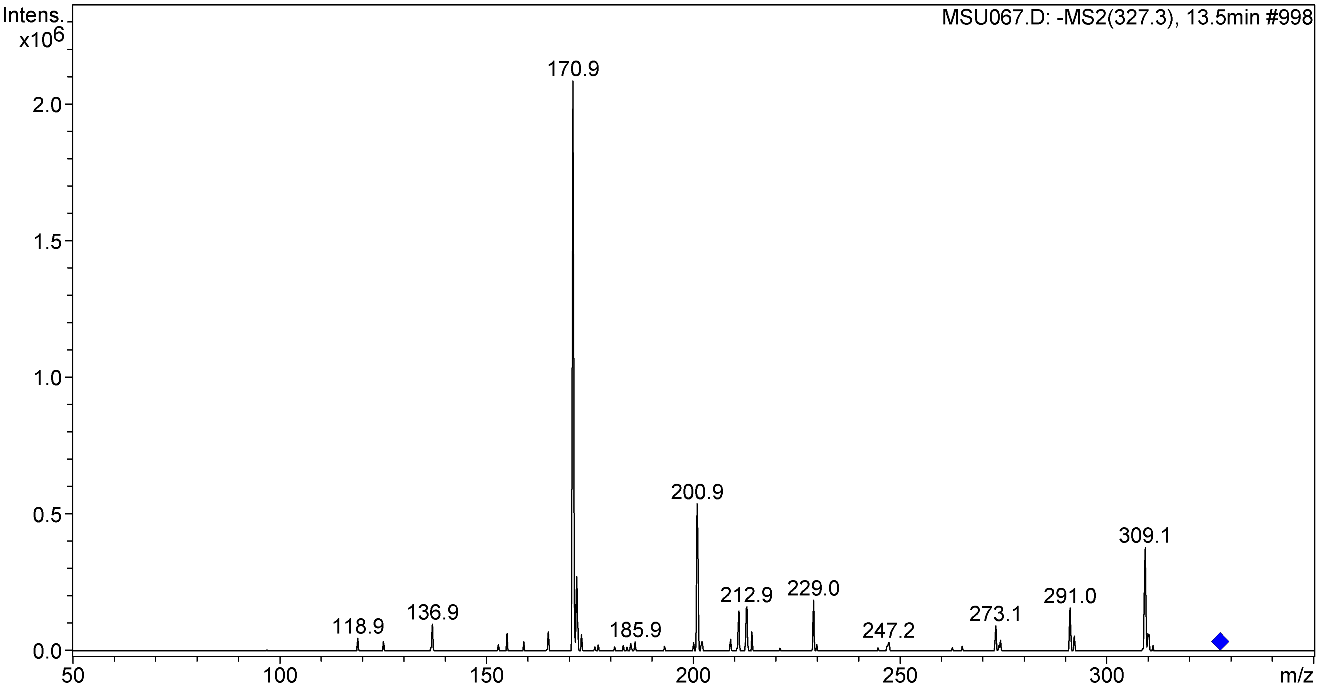

Mass List:

#	m/z	Res.	FWHM	I	I %	S/N
1	118.9	522	0.2	45961	2.2	2.7
2	125.1	543	0.2	32366	1.6	1.9
3	136.9	502	0.3	97377	4.7	5.7
4	152.9	604	0.3	22882	1.1	1.3
5	155.0	600	0.3	63424	3.0	3.7
6	159.0	714	0.2	32747	1.6	1.9
7	165.0	585	0.3	68667	3.3	4.0
8	170.9	485	0.4	2083942	100.0	122.2
9	171.8	523	0.3	270977	13.0	15.9
10	173.0	649	0.3	58447	2.8	3.4
11	177.0	780	0.2	21527	1.0	1.3
12	184.9	640	0.3	25897	1.2	1.5
13	185.9	689	0.3	33109	1.6	1.9
14	200.1	763	0.3	29491	1.4	1.7
15	200.9	498	0.4	537615	25.8	31.5
16	202.1	454	0.4	33675	1.6	2.0
17	209.0	728	0.3	42663	2.0	2.5
18	211.0	703	0.3	145798	7.0	8.6
19	212.9	556	0.4	160862	7.7	9.4
20	214.1	845	0.3	69156	3.3	4.1
21	229.0	839	0.3	185266	8.9	10.9
22	229.8	1013	0.2	23688	1.1	1.4
23	247.2	368	0.7	31149	1.5	1.8
24	273.1	915	0.3	91740	4.4	5.4
25	274.2	1135	0.2	38885	1.9	2.3

Compound Mass Spectrum List Report - MS

#	m/z	Res.	FWHM	I	I %	S/N
26	291.0	848	0.3	156807	7.5	9.2
27	292.0	953	0.3	53801	2.6	3.2
28	309.1	724	0.4	378843	18.2	22.2
29	309.9	654	0.5	62392	3.0	3.7
30	310.0	637	0.5	58764	2.8	3.4

Display Report - Selected Window Selected

Analysis Name: MSU068.D
Method: GENERA~4.M
Sample Na TPQ-1-20
Analysis Inf

Instrume LC-MSD-Trap-SL
Operator: Pablo

Print Da 03/07/2 01:13:28
Acq. Dat 02/27/2019 019 PM
12:58:07 PM

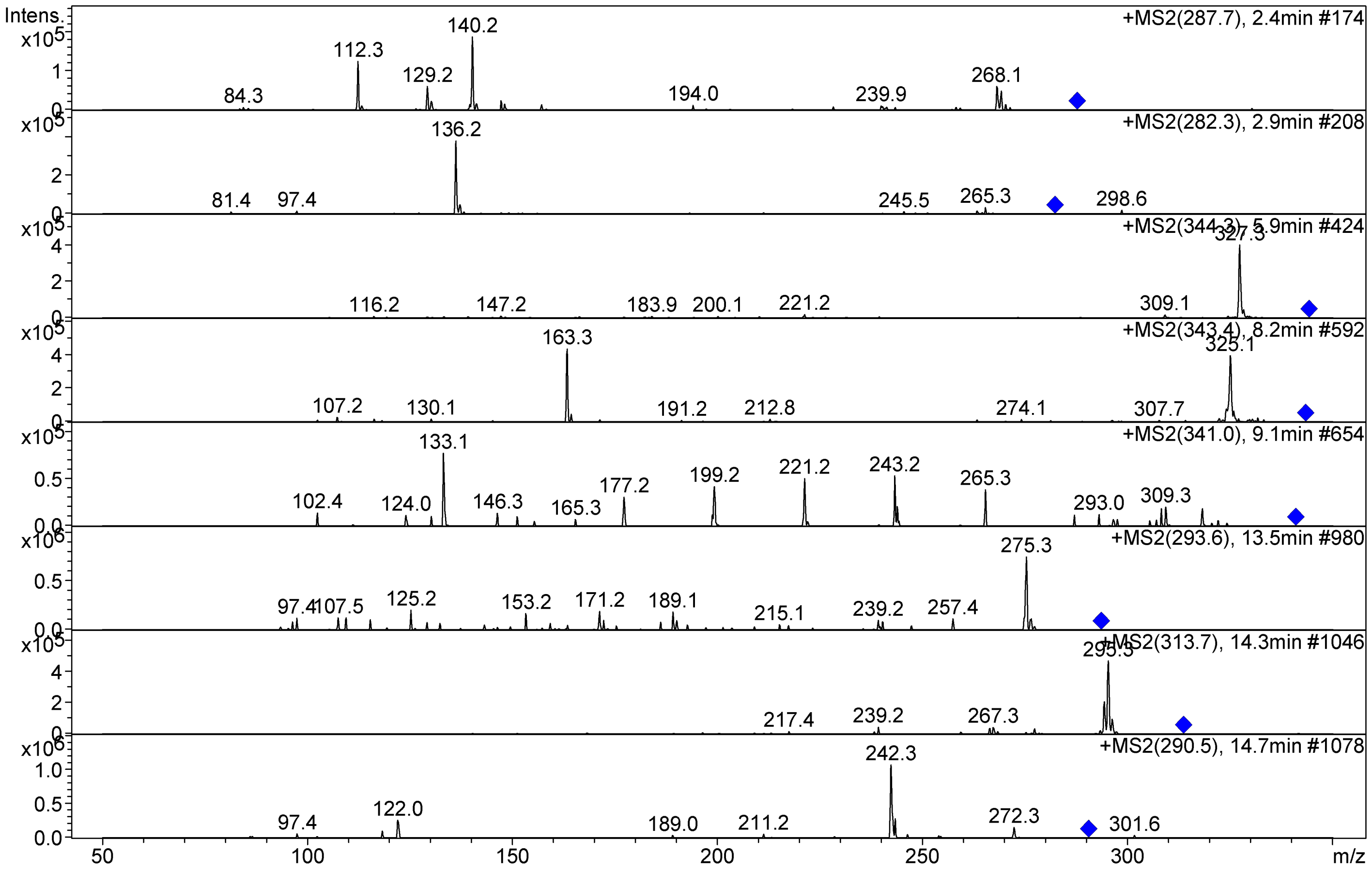

Agilent Technologies

Compound Mass Spectrum List Report - MS

**Analysis Name:**	MSU068.D	**Instrument:**	LC-MSD-Trap-SL	**Print Date:**	03/07/2019 03:33:56 PM
**Method:**	GENERA~4.M	**Operator:**	Pablo	**Acq. Date:**	02/27/2019 12:58:07 PM
**Sample Name:**	TPQ-1-20				
**Analysis Info:**					

Acquisition Parameter:

Mass Range Mode	Std/Normal	Trap Drive	49.6	Scan Begin	50 m/z
Ion Polarity	Positive	Octopole RF Amplitude	120.0 Vpp	Scan End	350 m/z
Ion Source Type	ESI	Capillary Exit	106.0 Volt	Averages	4 Spectra
Dry Temp (Set)	350 °C	Skimmer	40.0 Volt	Max. Accu Time	200000 µs
Nebulizer (Set)	70.00 psi	Oct 1 DC	12.00 Volt	ICC Target	30000
Dry Gas (Set)	12.00 l/min	Oct 2 DC	1.70 Volt	Charge Control	on

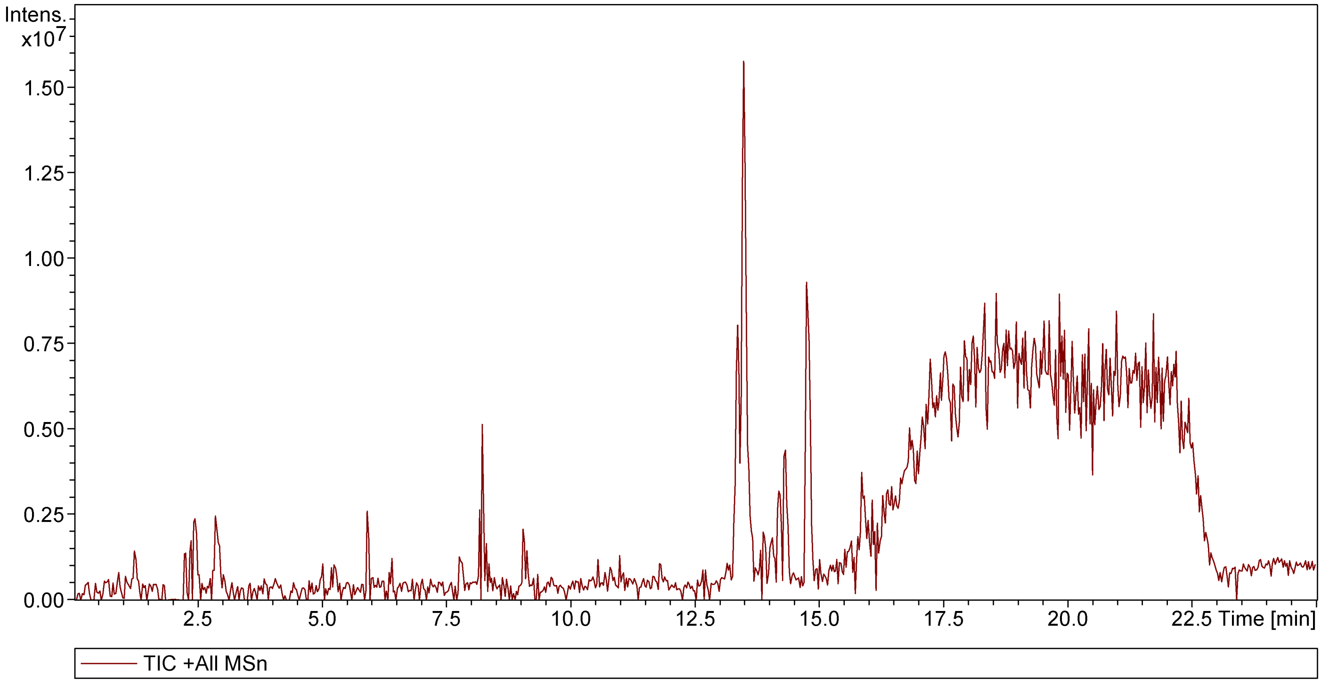

Compound List:

#	RT [min]	Range [min]	Height	Area	Area Frac %
n.a.	2.4	2.4	n.a.	n.a.	n.a.
n.a.	2.9	2.9	n.a.	n.a.	n.a.
n.a.	5.9	5.9	n.a.	n.a.	n.a.
n.a.	8.2	8.2	n.a.	n.a.	n.a.
n.a.	9.1	9.1	n.a.	n.a.	n.a.
n.a.	13.5	13.5	n.a.	n.a.	n.a.
n.a.	14.3	14.3	n.a.	n.a.	n.a.
n.a.	14.7	14.7	n.a.	n.a.	n.a.

Compound Mass Spectrum List Report - MS

+MS2(287.7), 2.4min #174

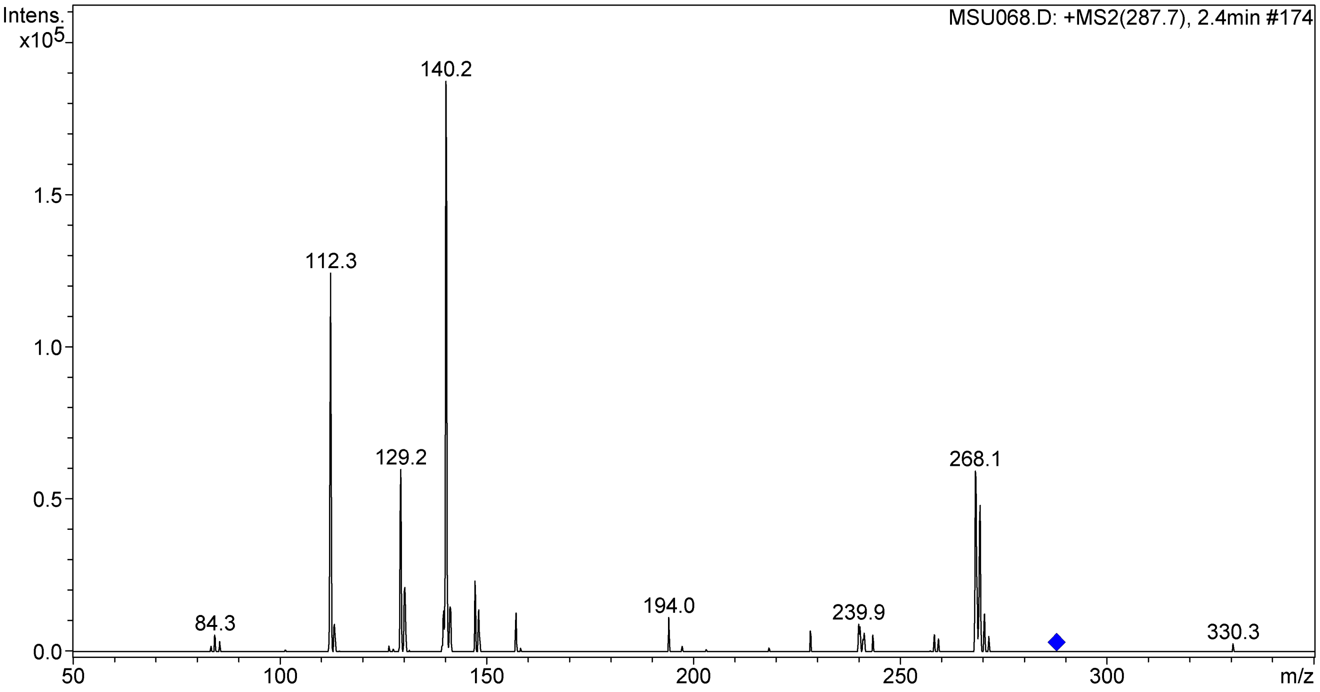

Mass List:

#	m/z	Res.	FWHM	I	I %	S/N
1	84.3	346	0.2	5373	2.9	3.0
2	85.5	387	0.2	3300	1.8	1.9
3	112.3	356	0.3	124156	66.3	69.9
4	113.2	284	0.4	8910	4.8	5.0
5	126.4	571	0.2	1782	1.0	1.0
6	129.2	381	0.3	59742	31.9	33.6
7	130.2	294	0.4	21003	11.2	11.8
8	139.6	638	0.2	13320	7.1	7.5
9	140.2	414	0.3	187189	100.0	105.4
10	141.2	325	0.4	14635	7.8	8.2
11	147.2	596	0.2	23174	12.4	13.0
12	148.1	532	0.3	13734	7.3	7.7
13	157.1	547	0.3	12631	6.7	7.1
14	194.0	869	0.2	11175	6.0	6.3
15	228.2	972	0.2	6719	3.6	3.8
16	239.9	1106	0.2	8999	4.8	5.1
17	240.2	1180	0.2	8075	4.3	4.5
18	241.2	448	0.5	6032	3.2	3.4
19	243.3	1040	0.2	5336	2.9	3.0
20	258.2	994	0.3	5432	2.9	3.1
21	259.1	1029	0.3	4095	2.2	2.3
22	268.1	694	0.4	59232	31.6	33.4
23	269.2	791	0.3	47914	25.6	27.0
24	270.3	975	0.3	12367	6.6	7.0
25	271.3	1190	0.2	4960	2.6	2.8

Compound Mass Spectrum List Report - MS

#	m/z	Res.	FWHM	I	I %	S/N
26	330.3	1407	0.2	2571	1.4	1.4

Compound Mass Spectrum List Report - MS

+MS2(282.3), 2.9min #208

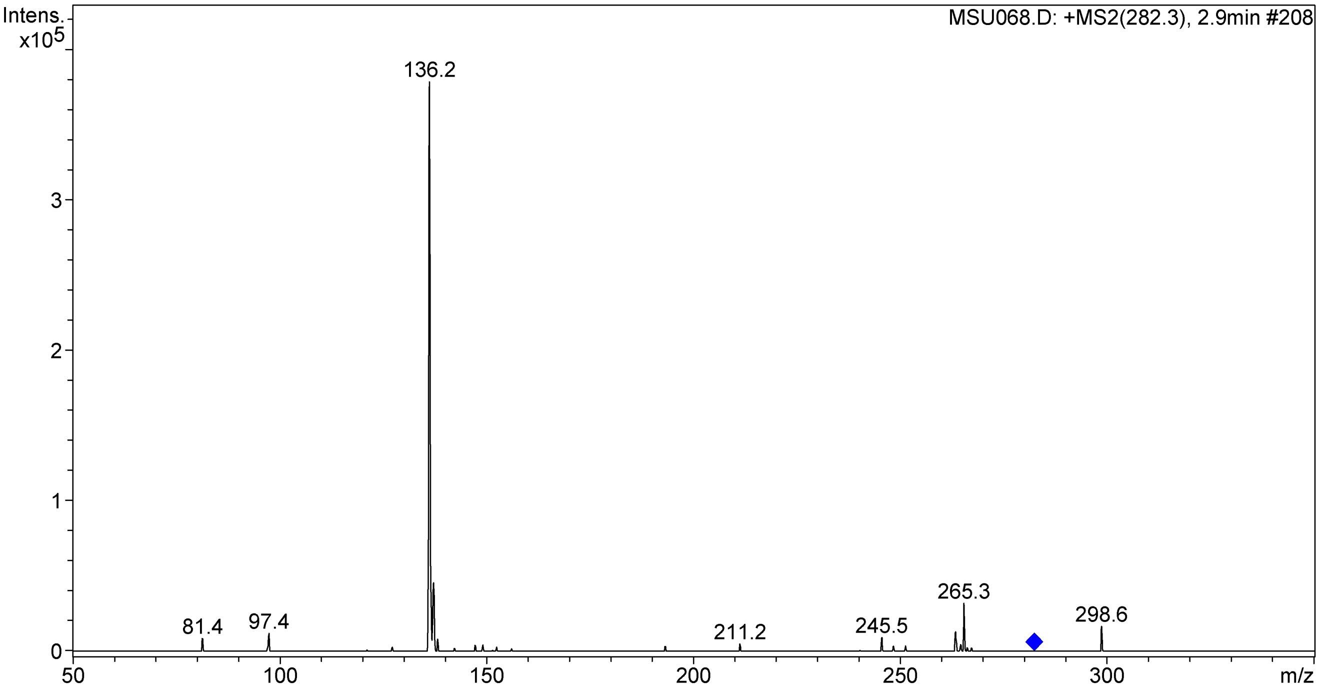

Mass List:

#	m/z	Res.	FWHM	I	I %	S/N
1	81.4	314	0.3	8442	2.2	2.3
2	97.4	365	0.3	11844	3.1	3.3
3	136.2	372	0.4	378347	100.0	104.6
4	137.2	325	0.4	45311	12.0	12.5
5	138.2	544	0.3	7863	2.1	2.2
6	147.2	598	0.2	3860	1.0	1.1
7	149.1	600	0.2	4039	1.1	1.1
8	211.2	928	0.2	4806	1.3	1.3
9	245.5	939	0.3	8904	2.4	2.5
10	263.3	775	0.3	12880	3.4	3.6
11	264.5	960	0.3	4245	1.1	1.2
12	265.3	1120	0.2	31833	8.4	8.8
13	298.6	1156	0.3	16399	4.3	4.5

+MS2(344.3), 5.9min #424

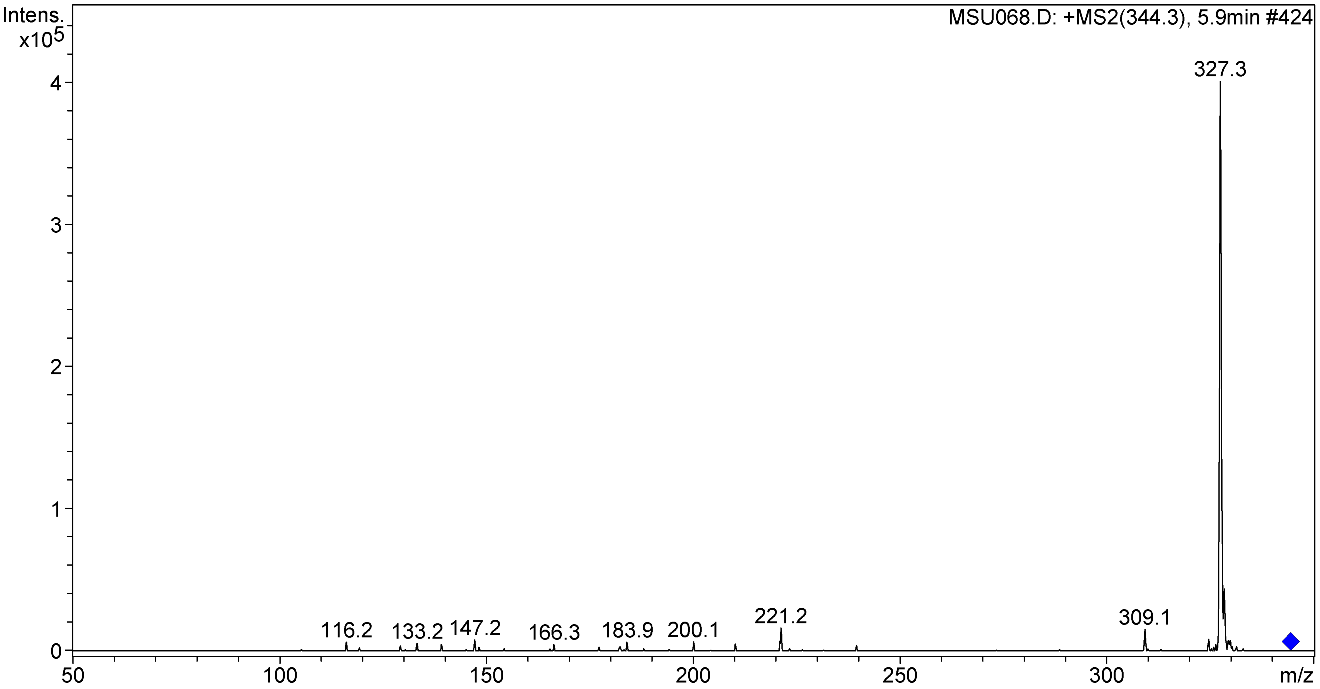

Mass List:

#	m/z	Res.	FWHM	I	I %	S/N
1	116.2	448	0.3	6026	1.5	4.4
2	119.3	512	0.2	2103	0.5	1.5
3	129.2	491	0.3	3341	0.8	2.5
4	133.2	385	0.3	5159	1.3	3.8
5	139.1	538	0.3	4570	1.1	3.4
6	147.2	525	0.3	7596	1.9	5.6
7	148.2	668	0.2	2605	0.7	1.9
8	166.3	702	0.2	4560	1.1	3.4
9	177.2	656	0.3	2439	0.6	1.8
10	182.3	411	0.4	2986	0.7	2.2
11	183.9	722	0.3	6104	1.5	4.5
12	188.0	770	0.2	1483	0.4	1.1
13	200.1	792	0.3	6309	1.6	4.6
14	210.2	805	0.3	4777	1.2	3.5
15	221.2	828	0.3	16056	4.0	11.8
16	223.2	958	0.2	1703	0.4	1.3
17	239.4	1050	0.2	3917	1.0	2.9
18	309.1	894	0.3	15026	3.8	11.1
19	309.8	1237	0.3	1477	0.4	1.1
20	324.5	1073	0.3	8053	2.0	5.9
21	325.1	1265	0.3	1492	0.4	1.1
22	325.7	1462	0.2	2604	0.6	1.9
23	326.2	1542	0.2	4648	1.2	3.4
24	327.3	733	0.4	400639	100.0	295.3
25	328.3	1041	0.3	43410	10.8	32.0

Compound Mass Spectrum List Report - MS

#	m/z	Res.	FWHM	I	I %	S/N
26	329.2	1210	0.3	7184	1.8	5.3
27	329.7	1515	0.2	7260	1.8	5.4
28	330.1	1408	0.2	2858	0.7	2.1
29	331.2	1140	0.3	2948	0.7	2.2

Compound Mass Spectrum List Report - MS

+MS2(343.4), 8.2min #592

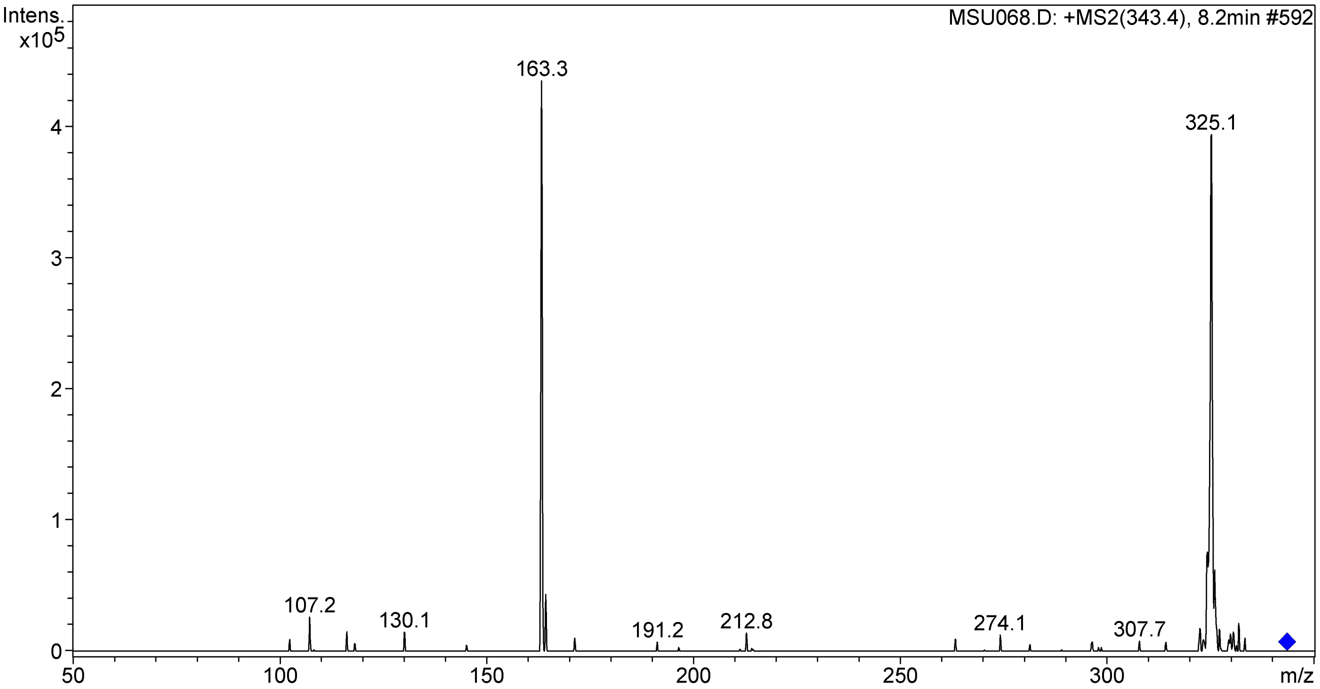

Mass List:

#	m/z	Res.	FWHM	I	I %	S/N
1	102.4	424	0.2	8797	2.0	2.5
2	107.2	417	0.3	25804	5.9	7.4
3	116.2	520	0.2	14737	3.4	4.2
4	118.1	463	0.3	5707	1.3	1.6
5	130.1	505	0.3	14497	3.3	4.2
6	145.1	569	0.3	4363	1.0	1.3
7	163.3	432	0.4	434601	100.0	125.0
8	164.3	628	0.3	43310	10.0	12.5
9	171.3	674	0.3	9896	2.3	2.8
10	191.2	862	0.2	6773	1.6	1.9
11	212.8	825	0.3	13817	3.2	4.0
12	263.3	1011	0.3	9038	2.1	2.6
13	274.1	1200	0.2	12166	2.8	3.5
14	281.2	1135	0.2	4944	1.1	1.4
15	296.2	731	0.4	6994	1.6	2.0
16	307.7	1252	0.2	7489	1.7	2.2
17	314.1	1121	0.3	6520	1.5	1.9
18	322.3	817	0.4	17168	4.0	4.9
19	323.2	679	0.5	8620	2.0	2.5
20	324.1	943	0.3	75469	17.4	21.7
21	325.1	681	0.5	393544	90.6	113.2
22	325.9	949	0.3	61747	14.2	17.8
23	327.0	1283	0.3	16724	3.8	4.8
24	329.3	1123	0.3	8479	2.0	2.4
25	329.7	1291	0.3	12896	3.0	3.7

Compound Mass Spectrum List Report - MS

#	m/z	Res.	FWHM	I	I %	S/N
26	330.4	878	0.4	14443	3.3	4.2
27	331.1	1403	0.2	4258	1.0	1.2
28	331.7	1400	0.2	21148	4.9	6.1
29	333.2	1320	0.3	9785	2.3	2.8

Compound Mass Spectrum List Report - MS

+MS2(341.0), 9.1min #654

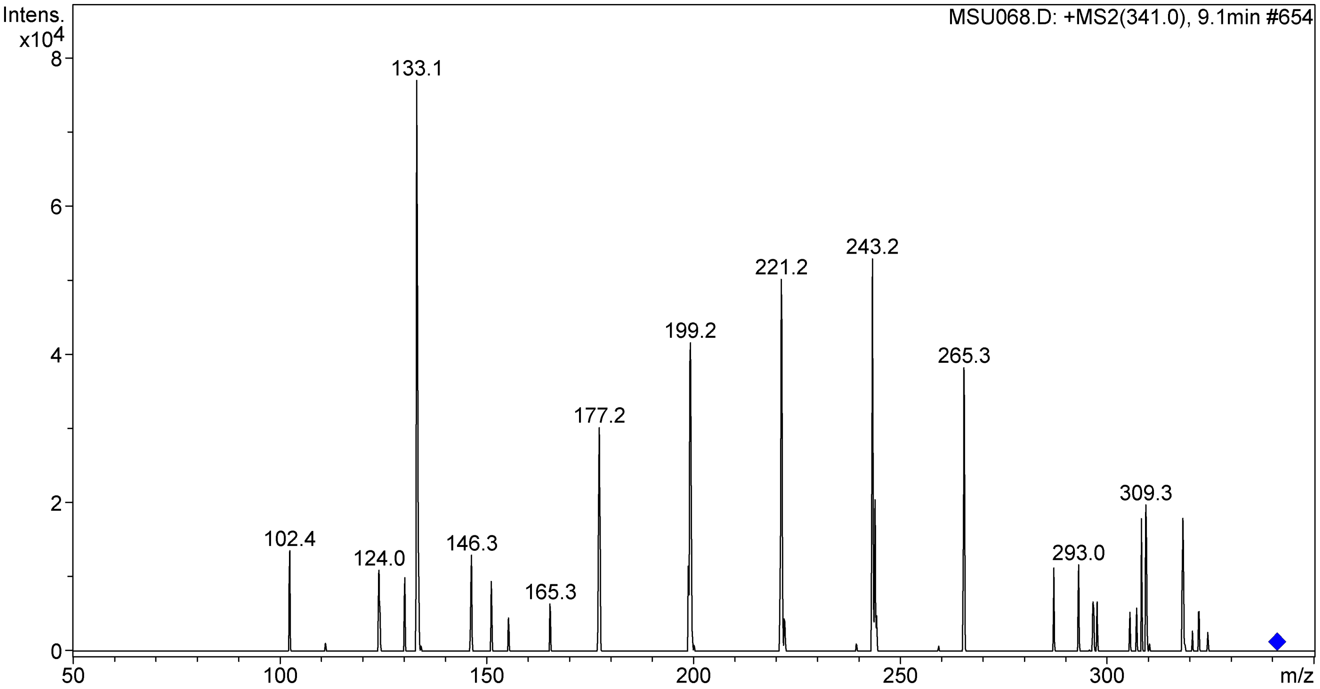

Mass List:

#	m/z	Res.	FWHM	I	I %	S/N
1	102.4	413	0.2	13535	17.6	4.0
2	124.0	330	0.4	10906	14.2	3.2
3	130.2	532	0.2	9892	12.9	2.9
4	133.1	368	0.4	76935	100.0	22.6
5	146.3	510	0.3	12934	16.8	3.8
6	151.1	618	0.2	9376	12.2	2.8
7	155.3	608	0.3	4447	5.8	1.3
8	165.3	695	0.2	6368	8.3	1.9
9	177.2	449	0.4	30110	39.1	8.8
10	198.7	1311	0.2	11447	14.9	3.4
11	199.2	482	0.4	41555	54.0	12.2
12	221.2	621	0.4	50107	65.1	14.7
13	221.9	636	0.3	4308	5.6	1.3
14	243.2	903	0.3	52873	68.7	15.5
15	243.8	981	0.2	20402	26.5	6.0
16	265.3	962	0.3	38191	49.6	11.2
17	287.0	1282	0.2	11208	14.6	3.3
18	293.0	1253	0.2	11612	15.1	3.4
19	296.5	717	0.4	6596	8.6	1.9
20	297.5	1195	0.2	6606	8.6	1.9
21	305.4	1365	0.2	5228	6.8	1.5
22	307.0	1353	0.2	5791	7.5	1.7
23	308.2	1386	0.2	17861	23.2	5.2
24	309.3	912	0.3	19710	25.6	5.8
25	318.2	885	0.4	17888	23.3	5.3

Compound Mass Spectrum List Report - MS

#	m/z	Res.	FWHM	I	I %	S/N
26	322.1	1238	0.3	5323	6.9	1.6

Compound Mass Spectrum List Report - MS

+MS2(293.6), 13.5min #980

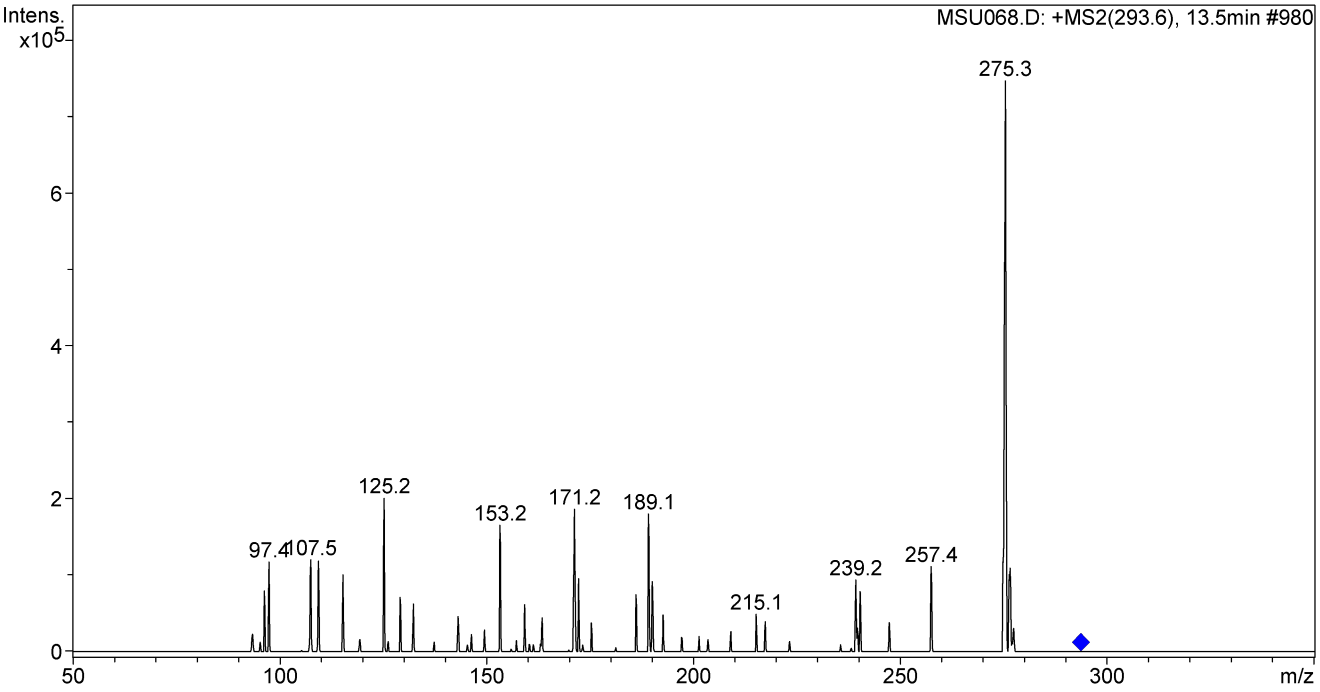

Mass List:

#	m/z	Res.	FWHM	I	I %	S/N
1	96.3	424	0.2	79437	10.6	4.4
2	97.4	408	0.2	117022	15.7	6.5
3	107.5	390	0.3	120144	16.1	6.7
4	109.4	404	0.3	118392	15.9	6.6
5	115.3	460	0.3	100443	13.5	5.6
6	125.2	493	0.3	200409	26.9	11.1
7	129.1	533	0.2	70956	9.5	3.9
8	132.3	514	0.3	62030	8.3	3.4
9	143.1	470	0.3	45586	6.1	2.5
10	153.2	608	0.3	165170	22.1	9.2
11	159.2	627	0.3	61216	8.2	3.4
12	163.4	720	0.2	44128	5.9	2.4
13	171.2	520	0.3	185970	24.9	10.3
14	172.2	775	0.2	95163	12.7	5.3
15	175.3	783	0.2	37250	5.0	2.1
16	186.1	782	0.2	74420	10.0	4.1
17	189.1	705	0.3	179683	24.1	10.0
18	190.0	602	0.3	91311	12.2	5.1
19	192.6	783	0.2	47961	6.4	2.7
20	215.1	903	0.2	48820	6.5	2.7
21	217.3	888	0.2	39069	5.2	2.2
22	239.2	942	0.3	93508	12.5	5.2
23	239.6	1349	0.2	30718	4.1	1.7
24	240.3	919	0.3	78569	10.5	4.4
25	247.3	923	0.3	37783	5.1	2.1

Compound Mass Spectrum List Report - MS

#	m/z	Res.	FWHM	I	I %	S/N
26	257.4	831	0.3	111114	14.9	6.2
27	275.3	571	0.5	746385	100.0	41.4
28	276.3	504	0.5	100495	13.5	5.6
29	276.4	520	0.5	109061	14.6	6.1
30	277.3	813	0.3	30378	4.1	1.7

Compound Mass Spectrum List Report - MS

+MS2(313.7), 14.3min #1046

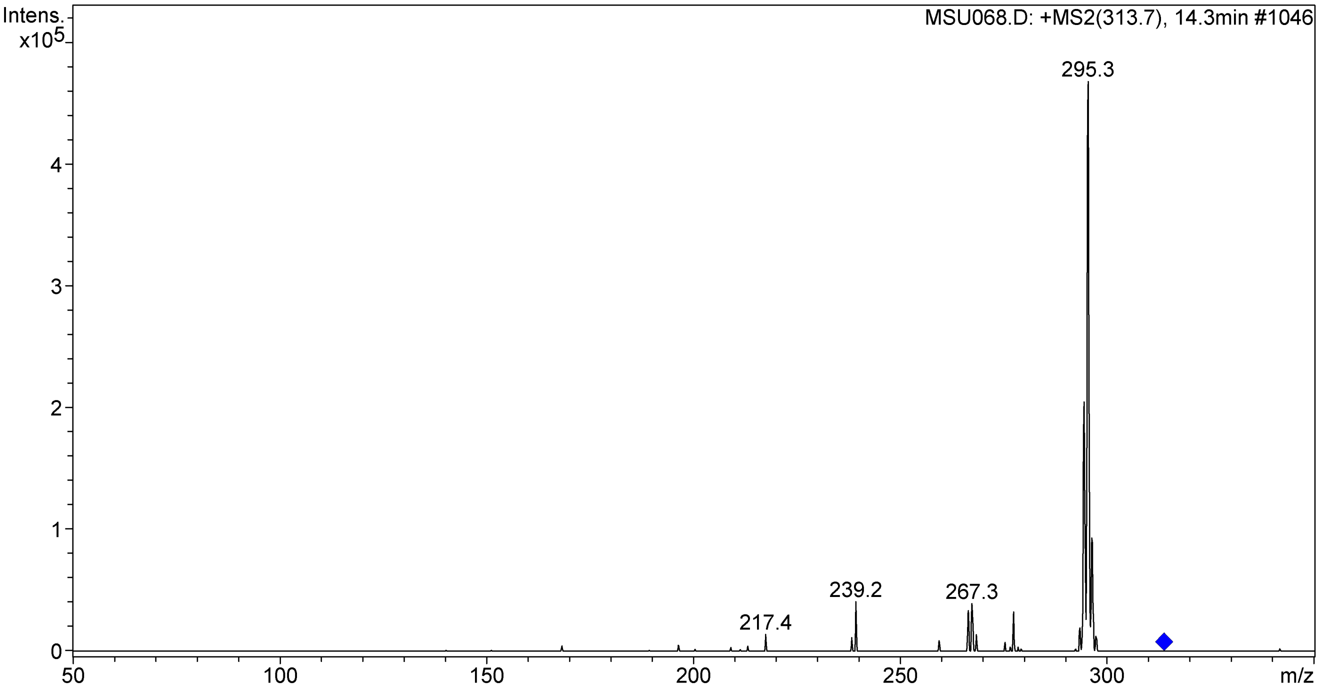

Mass List:

#	m/z	Res.	FWHM	I	I %	S/N
1	168.2	687	0.2	4247	0.9	1.1
2	196.3	772	0.3	4885	1.0	1.2
3	213.1	866	0.2	4118	0.9	1.0
4	217.4	981	0.2	13773	2.9	3.4
5	238.2	1059	0.2	11092	2.4	2.8
6	239.2	1047	0.2	40422	8.6	10.0
7	259.3	841	0.3	8386	1.8	2.1
8	266.4	832	0.3	33125	7.1	8.2
9	267.3	643	0.4	38923	8.3	9.7
10	268.3	1087	0.2	13362	2.9	3.3
11	275.2	1189	0.2	7103	1.5	1.8
12	277.3	988	0.3	32031	6.8	8.0
13	293.3	878	0.3	19028	4.1	4.7
14	294.3	734	0.4	204529	43.7	50.8
15	295.3	641	0.5	467778	100.0	116.3
16	296.2	772	0.4	92880	19.9	23.1
17	297.2	686	0.4	12108	2.6	3.0

Compound Mass Spectrum List Report - MS

+MS2(290.5), 14.7min #1078

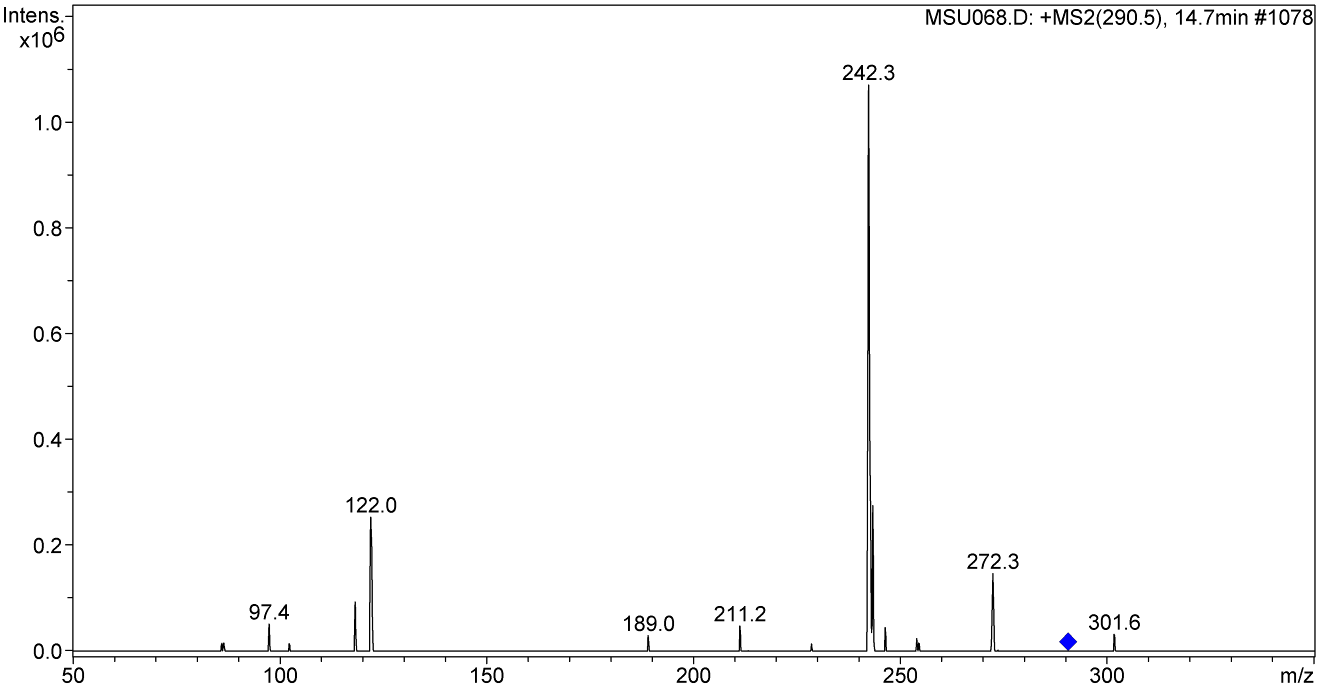

Mass List:

#	m/z	Res.	FWHM	I	I %	S/N
1	97.4	377	0.3	50818	4.7	1.9
2	118.2	464	0.3	92802	8.7	3.5
3	122.0	248	0.5	253340	23.7	9.6
4	189.0	766	0.2	29059	2.7	1.1
5	211.2	935	0.2	47544	4.4	1.8
6	242.3	602	0.4	1069904	100.0	40.4
7	243.3	1008	0.2	275159	25.7	10.4
8	246.3	1081	0.2	44240	4.1	1.7
9	272.3	733	0.4	146886	13.7	5.5
10	301.6	1185	0.3	31549	2.9	1.2

Display Report - Selected Window Selected

Analysis Name: MSU069.D
Method: GENERA~4.M
Sample Na TPQ-2-20
Analysis Inf

Instrume LC-MSD-Trap-SL
Operator: Pablo

Print Da 03/07/2 03:37:27 019 PM
Acq. Dat 02/27/2019 01:26:16 PM

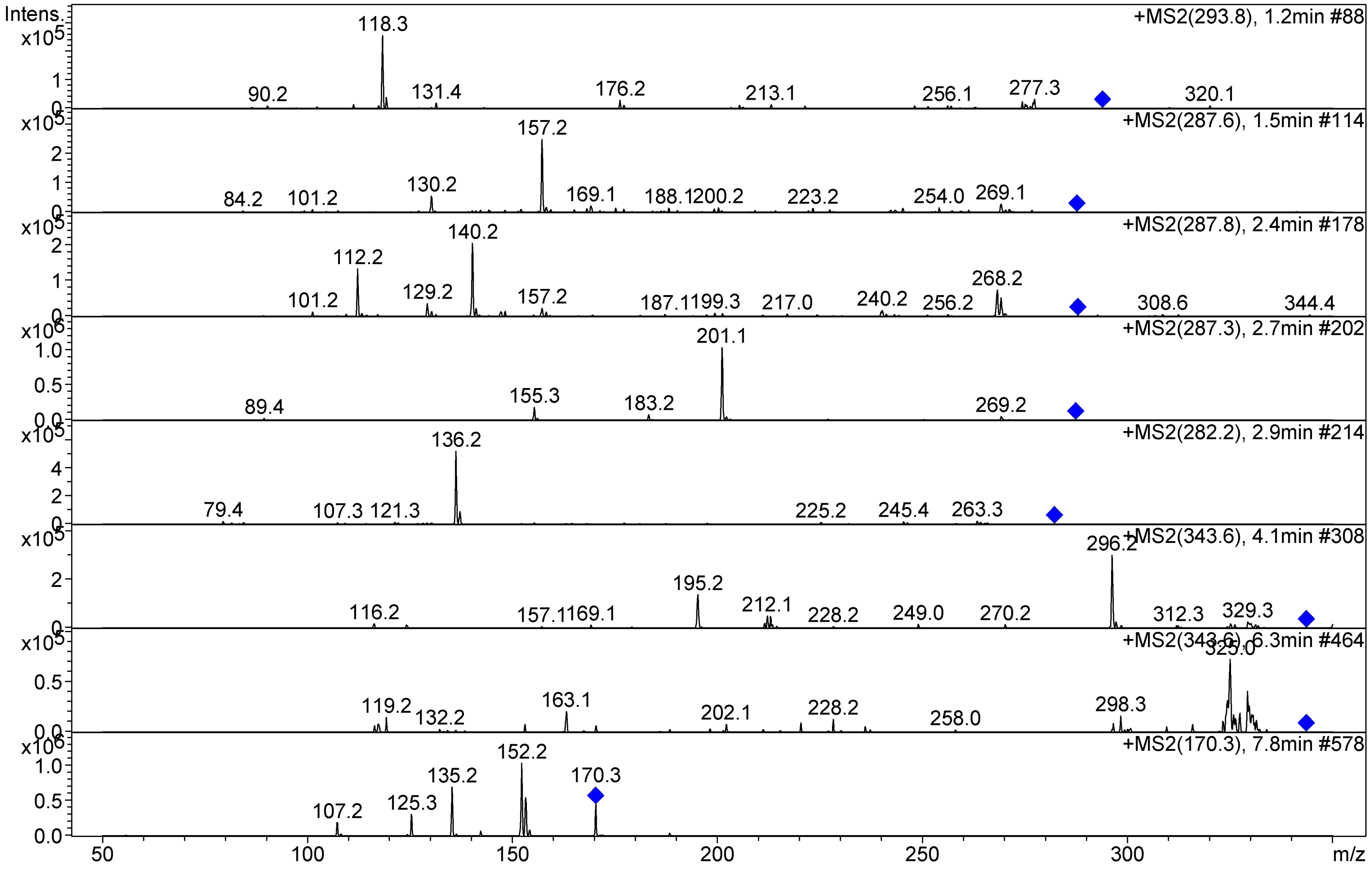

Agilent Technologies

Display Report - Selected Window Selected

Analysis Name: MSU069.D
Method: GENERA~4.M
Sample Na TPQ-2-20
Analysis Inf

Instrume LC-MSD-Trap-SL
Operator: Pablo

Print Da 03/07/2 03:37:50
Acq. Dat 02/27/2019 019 PM
01:26:16 PM

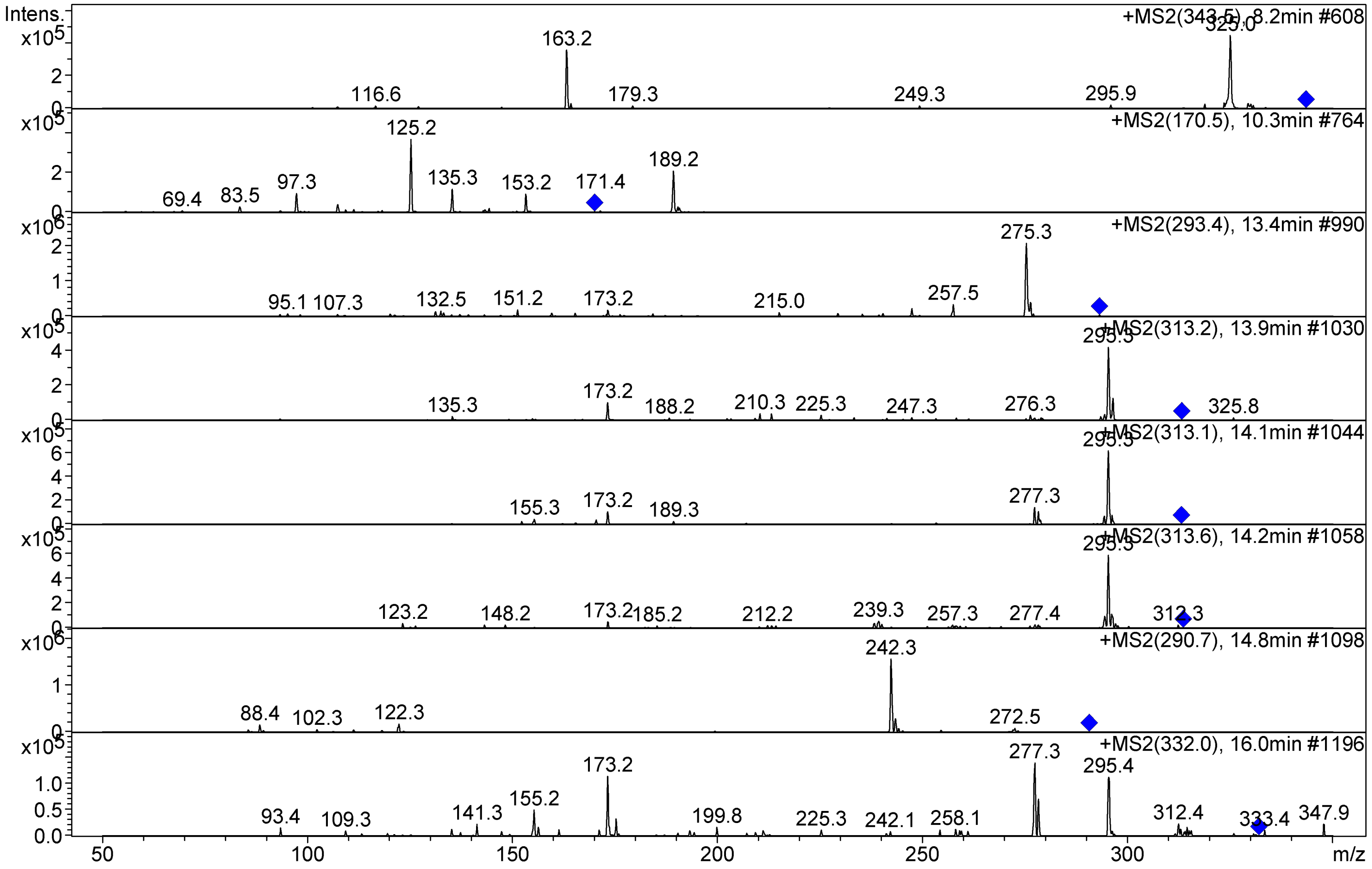

Compound Mass Spectrum List Report - MS

Analysis Name: MSU069.D **Instrument:** LC-MSD-Trap-SL **Print Date:** 03/07/2019 03:36:14 PM
Method: GENERA~4.M **Operator:** Pablo **Acq. Date:** 02/27/2019 01:26:16 PM
Sample Name: TPQ-2-20
Analysis Info:

Acquisition Parameter:

Mass Range Mode	Std/Normal	Trap Drive	49.6	Scan Begin	50 m/z
Ion Polarity	Positive	Octopole RF Amplitude	120.0 Vpp	Scan End	350 m/z
Ion Source Type	ESI	Capillary Exit	106.0 Volt	Averages	4 Spectra
Dry Temp (Set)	350 °C	Skimmer	40.0 Volt	Max. Accu Time	200000 µs
Nebulizer (Set)	70.00 psi	Oct 1 DC	12.00 Volt	ICC Target	30000
Dry Gas (Set)	12.00 l/min	Oct 2 DC	1.70 Volt	Charge Control	on

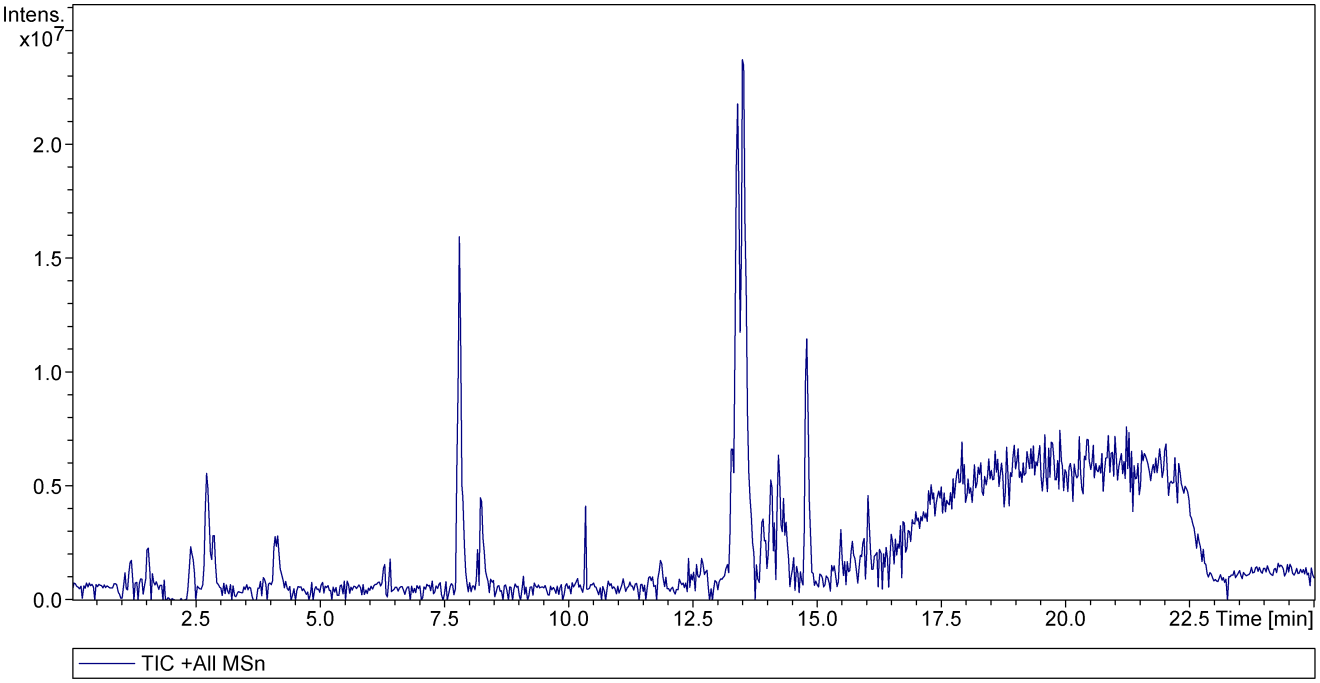

Compound List:

#	RT [min]	Range [min]	Height	Area	Area Frac %
n.a.	1.2	1.2	n.a.	n.a.	n.a.
n.a.	1.5	1.5	n.a.	n.a.	n.a.
n.a.	2.4	2.4	n.a.	n.a.	n.a.
n.a.	2.7	2.7	n.a.	n.a.	n.a.
n.a.	2.9	2.9	n.a.	n.a.	n.a.
n.a.	4.1	4.1	n.a.	n.a.	n.a.
n.a.	6.3	6.3	n.a.	n.a.	n.a.
n.a.	7.8	7.8	n.a.	n.a.	n.a.
n.a.	8.2	8.2	n.a.	n.a.	n.a.
n.a.	10.3	10.3	n.a.	n.a.	n.a.

Compound Mass Spectrum List Report - MS

#	RT [min]	Range [min]	Height	Area	Area Frac %
n.a.	13.4	13.4	n.a.	n.a.	n.a.
n.a.	13.9	13.9	n.a.	n.a.	n.a.
n.a.	14.1	14.1	n.a.	n.a.	n.a.
n.a.	14.2	14.2	n.a.	n.a.	n.a.
n.a.	14.8	14.8	n.a.	n.a.	n.a.
n.a.	16.0	16.0	n.a.	n.a.	n.a.

+MS2(293.8), 1.2min #88

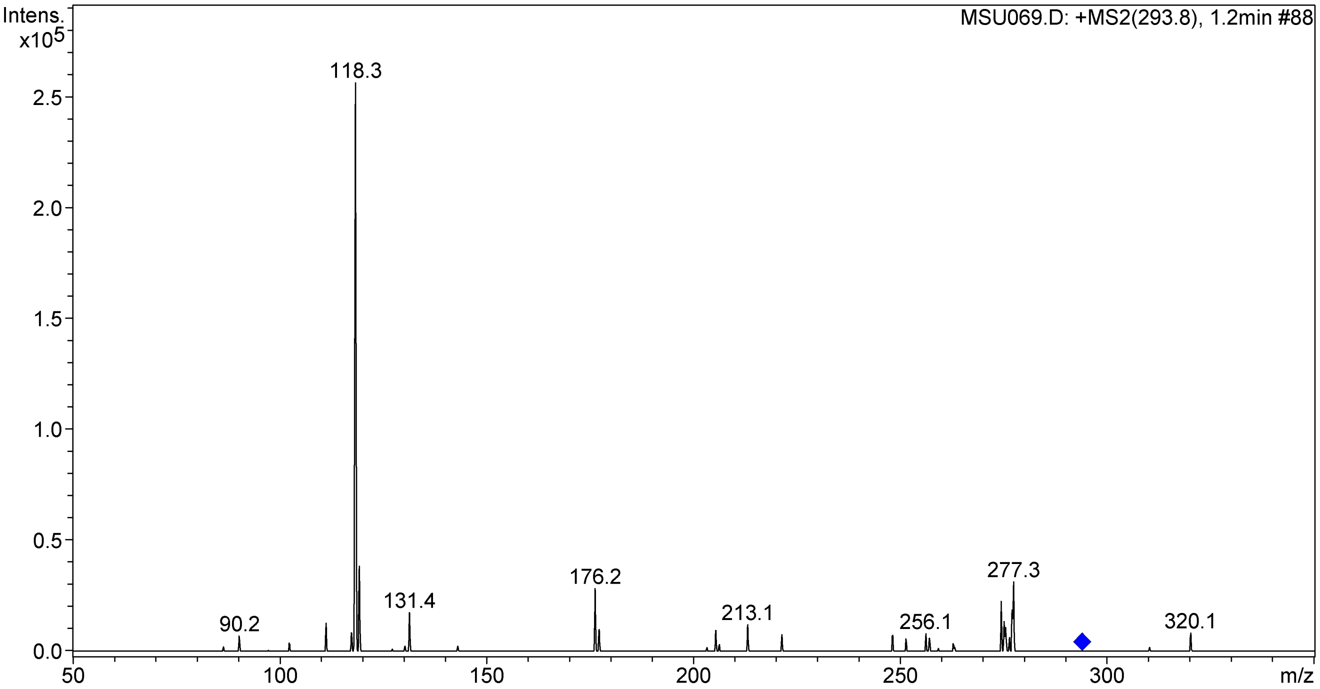

Mass List:

#	m/z	Res.	FWHM	I	I %	S/N
1	90.2	371	0.2	6768	2.6	1.8
2	111.2	501	0.2	12514	4.9	3.2
3	117.4	454	0.3	8267	3.2	2.1
4	118.3	368	0.3	256175	100.0	66.5
5	119.2	462	0.3	38213	14.9	9.9
6	131.4	503	0.3	17338	6.8	4.5
7	176.2	726	0.2	28127	11.0	7.3
8	177.2	677	0.3	9629	3.8	2.5
9	205.4	784	0.3	9289	3.6	2.4
10	213.1	826	0.3	11904	4.6	3.1
11	221.3	963	0.2	7423	2.9	1.9
12	248.1	956	0.3	7170	2.8	1.9
13	251.3	1106	0.2	5581	2.2	1.4
14	256.1	1151	0.2	7831	3.1	2.0
15	257.0	914	0.3	5811	2.3	1.5
16	274.3	1238	0.2	22402	8.7	5.8
17	275.0	1270	0.2	13311	5.2	3.5
18	275.4	1051	0.3	10632	4.2	2.8
19	276.3	1136	0.2	6048	2.4	1.6
20	277.0	1388	0.2	18526	7.2	4.8
21	277.3	1158	0.2	31160	12.2	8.1
22	320.1	1285	0.2	8042	3.1	2.1

Compound Mass Spectrum List Report - MS

+MS2(287.6), 1.5min #114

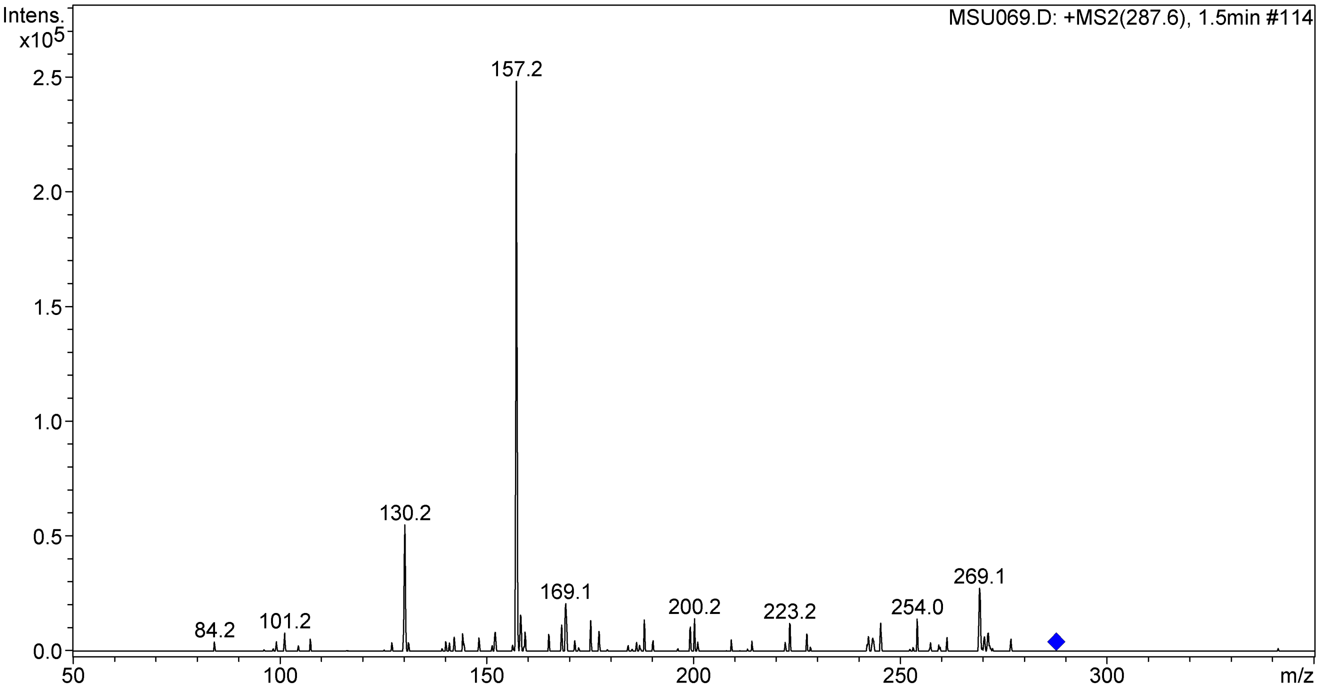

Mass List:

#	m/z	Res.	FWHM	I	I %	S/N
1	101.2	407	0.2	7783	3.1	3.5
2	107.4	486	0.2	5179	2.1	2.3
3	130.2	371	0.4	54962	22.2	24.4
4	142.2	553	0.3	6004	2.4	2.7
5	144.2	588	0.2	7482	3.0	3.3
6	148.1	578	0.3	5728	2.3	2.5
7	152.1	406	0.4	8131	3.3	3.6
8	157.2	456	0.3	248062	100.0	110.2
9	158.2	408	0.4	15576	6.3	6.9
10	159.3	633	0.3	8247	3.3	3.7
11	165.0	678	0.2	7335	3.0	3.3
12	168.1	646	0.3	11350	4.6	5.0
13	169.1	400	0.4	20662	8.3	9.2
14	175.1	726	0.2	13233	5.3	5.9
15	177.1	697	0.3	8589	3.5	3.8
16	188.1	776	0.2	13574	5.5	6.0
17	199.2	779	0.3	10464	4.2	4.7
18	200.2	876	0.2	14042	5.7	6.2
19	209.1	937	0.2	4906	2.0	2.2
20	223.2	864	0.3	11989	4.8	5.3
21	227.3	881	0.3	7420	3.0	3.3
22	242.2	804	0.3	6301	2.5	2.8
23	243.3	451	0.5	5655	2.3	2.5
24	245.2	788	0.3	12094	4.9	5.4
25	254.0	1115	0.2	13984	5.6	6.2

Compound Mass Spectrum List Report - MS

#	m/z	Res.	FWHM	I	I %	S/N
26	261.2	1162	0.2	5853	2.4	2.6
27	269.1	612	0.4	27328	11.0	12.1
28	270.2	768	0.4	6206	2.5	2.8
29	271.1	668	0.4	7811	3.1	3.5
30	276.7	1077	0.3	5168	2.1	2.3

Compound Mass Spectrum List Report - MS

+MS2(287.8), 2.4min #178

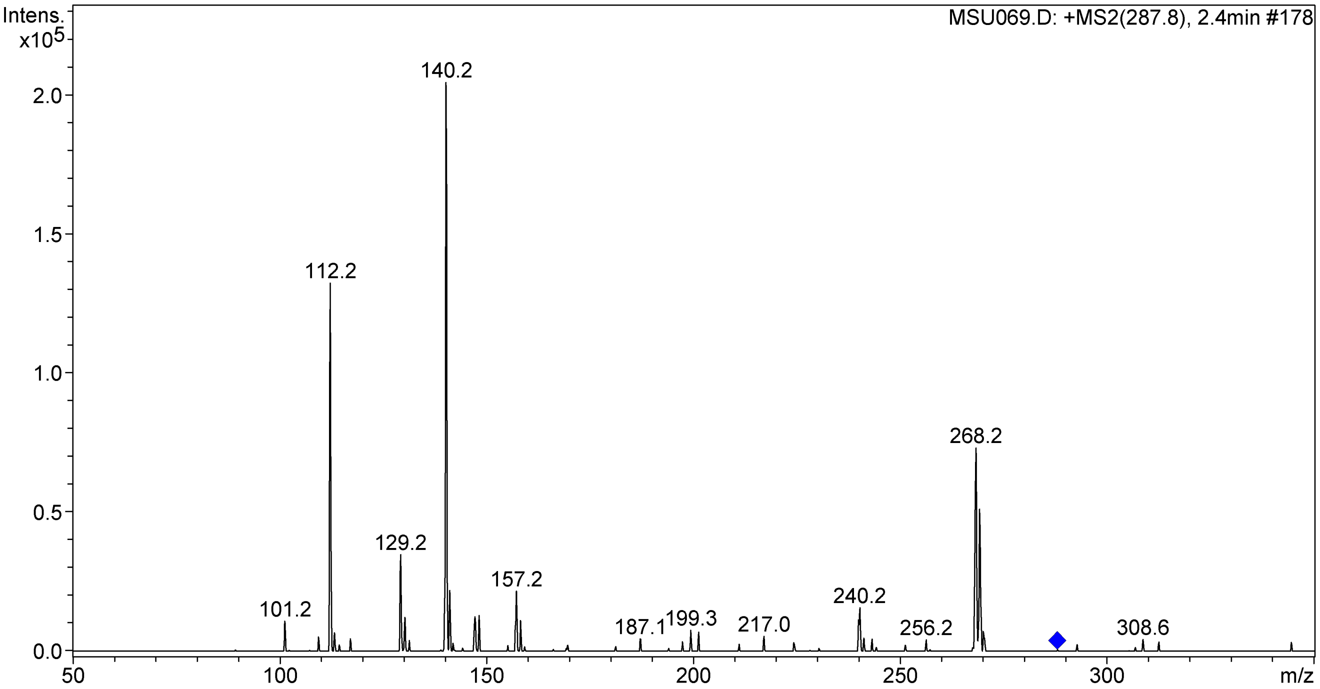

Mass List:

#	m/z	Res.	FWHM	I	I %	S/N
1	101.2	390	0.3	10727	5.2	7.6
2	109.4	483	0.2	5053	2.5	3.6
3	112.2	371	0.3	132262	64.7	93.1
4	113.2	419	0.3	6585	3.2	4.6
5	117.1	526	0.2	4408	2.2	3.1
6	129.2	401	0.3	34643	17.0	24.4
7	130.3	452	0.3	12067	5.9	8.5
8	131.3	581	0.2	3813	1.9	2.7
9	140.2	426	0.3	204357	100.0	143.9
10	141.1	480	0.3	21721	10.6	15.3
11	147.2	347	0.4	12283	6.0	8.6
12	148.2	588	0.3	12850	6.3	9.0
13	157.2	438	0.4	21592	10.6	15.2
14	158.2	605	0.3	10952	5.4	7.7
15	187.1	728	0.3	4424	2.2	3.1
16	197.3	894	0.2	3349	1.6	2.4
17	199.3	823	0.2	7449	3.6	5.2
18	201.2	868	0.2	6862	3.4	4.8
19	217.0	874	0.2	5268	2.6	3.7
20	239.9	1248	0.2	11137	5.4	7.8
21	240.2	912	0.3	15585	7.6	11.0
22	241.1	947	0.3	4677	2.3	3.3
23	243.1	1044	0.2	4277	2.1	3.0
24	256.2	1020	0.3	4013	2.0	2.8
25	268.2	597	0.4	72957	35.7	51.4

Compound Mass Spectrum List Report - MS

#	m/z	Res.	FWHM	I	I %	S/N
26	269.1	821	0.3	51017	25.0	35.9
27	270.1	609	0.4	7035	3.4	5.0
28	308.6	1193	0.3	4159	2.0	2.9
29	312.4	1276	0.2	3218	1.6	2.3
30	344.4	1522	0.2	3077	1.5	2.2

+MS2(287.3), 2.7min #202

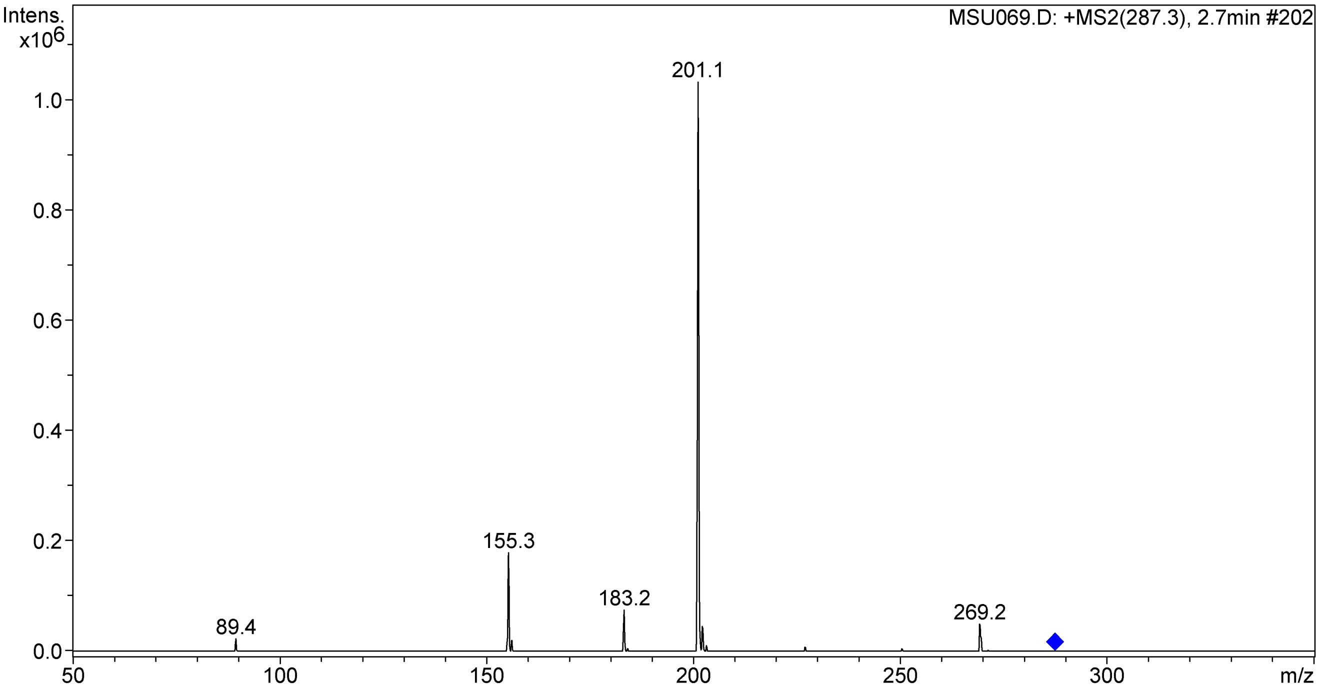

Mass List:

#	m/z	Res.	FWHM	I	I %	S/N
1	89.4	386	0.2	21922	2.1	2.3
2	155.3	518	0.3	178260	17.3	18.7
3	156.1	574	0.3	19264	1.9	2.0
4	183.2	587	0.3	74271	7.2	7.8
5	201.1	577	0.3	1031404	100.0	108.4
6	202.1	610	0.3	44477	4.3	4.7
7	203.1	917	0.2	10100	1.0	1.1
8	269.2	631	0.4	48960	4.7	5.1

Compound Mass Spectrum List Report - MS

+MS2(282.2), 2.9min #214

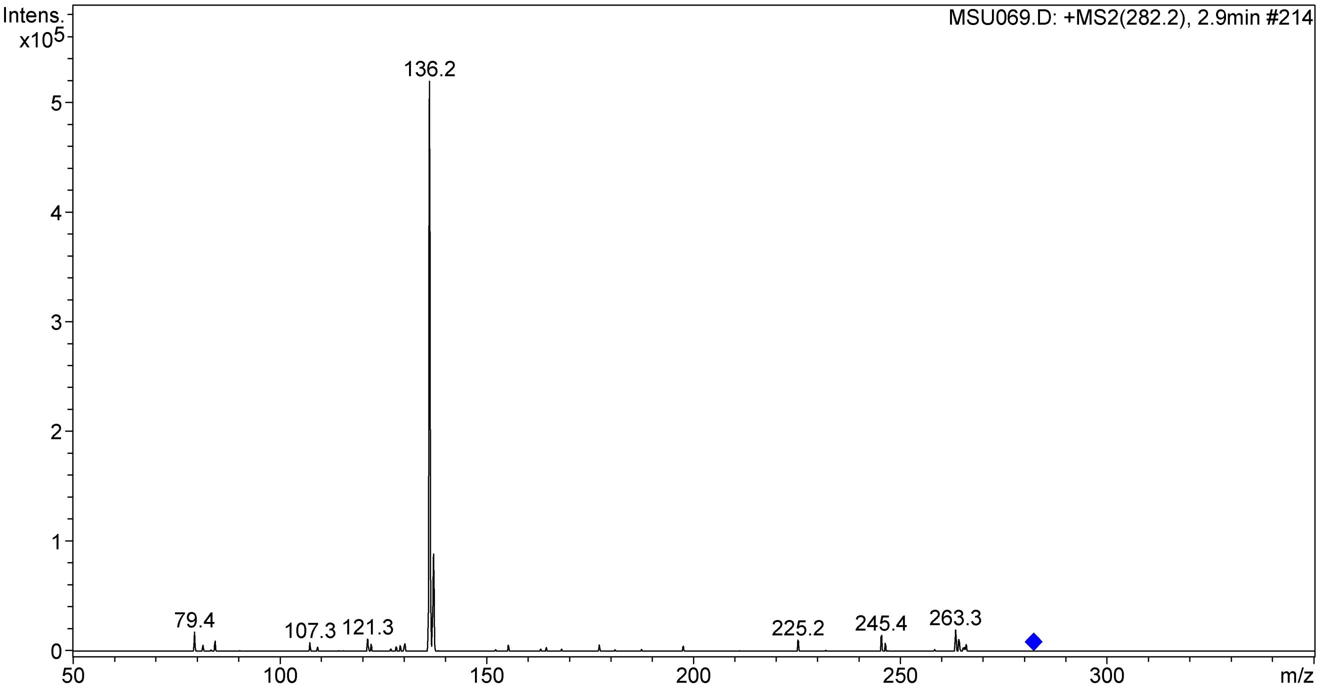

Mass List:

#	m/z	Res.	FWHM	I	I %	S/N
1	79.4	322	0.2	17204	3.3	5.6
2	81.5	323	0.3	5203	1.0	1.7
3	84.4	342	0.2	9022	1.7	3.0
4	107.3	451	0.2	7732	1.5	2.5
5	109.1	423	0.3	3573	0.7	1.2
6	121.3	455	0.3	10888	2.1	3.6
7	122.1	521	0.2	6214	1.2	2.0
8	128.1	504	0.3	3913	0.8	1.3
9	129.1	538	0.2	4901	0.9	1.6
10	130.2	380	0.3	6885	1.3	2.3
11	136.2	422	0.3	519055	100.0	169.8
12	137.2	438	0.3	88523	17.1	29.0
13	155.3	598	0.3	5166	1.0	1.7
14	164.4	724	0.2	3326	0.6	1.1
15	177.2	767	0.2	5547	1.1	1.8
16	197.5	782	0.3	4428	0.9	1.4
17	225.2	889	0.3	10001	1.9	3.3
18	245.4	922	0.3	14363	2.8	4.7
19	246.3	1113	0.2	7022	1.4	2.3
20	263.3	891	0.3	19194	3.7	6.3
21	264.1	765	0.3	10540	2.0	3.4
22	265.4	1410	0.2	3360	0.6	1.1
23	265.8	1110	0.2	5947	1.1	1.9

Compound Mass Spectrum List Report - MS

+MS2(343.6), 4.1min #308

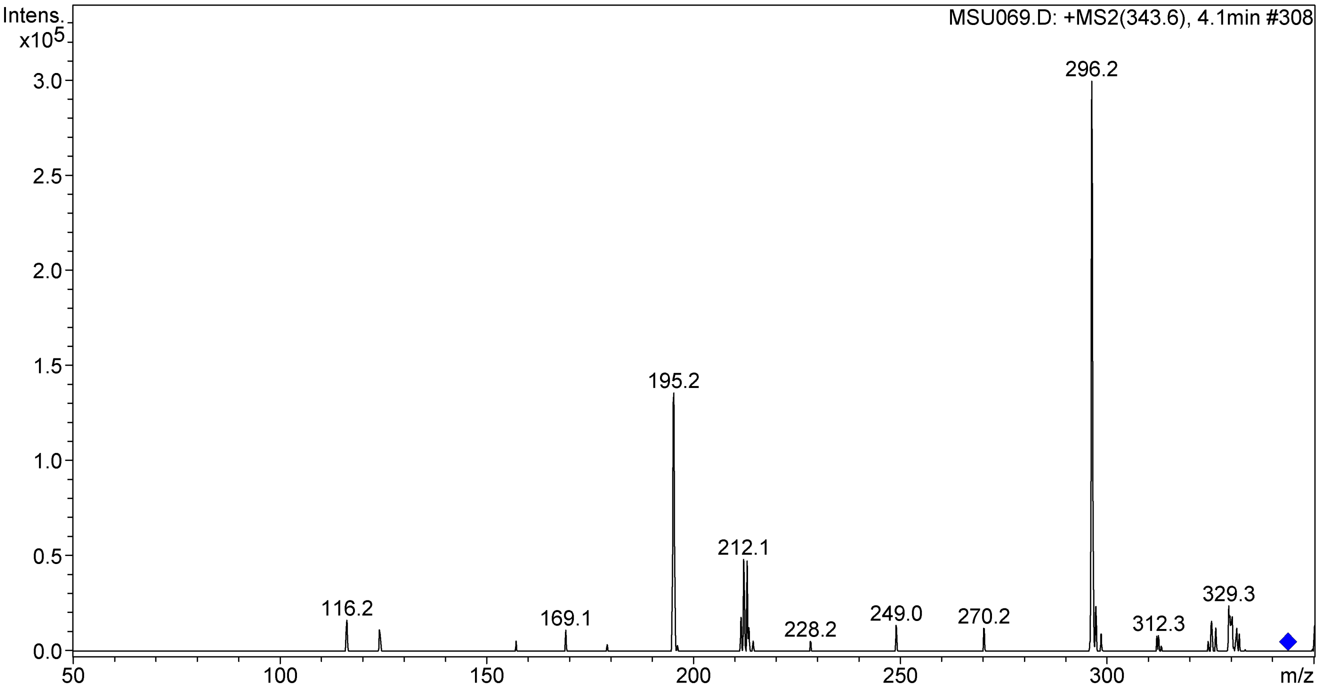

Mass List:

#	m/z	Res.	FWHM	I	I %	S/N
1	116.2	328	0.4	16207	5.4	3.6
2	124.1	298	0.4	11065	3.7	2.4
3	157.1	723	0.2	5216	1.7	1.1
4	169.1	760	0.2	10922	3.6	2.4
5	195.2	455	0.4	135514	45.3	29.7
6	211.5	786	0.3	17616	5.9	3.9
7	212.1	695	0.3	48199	16.1	10.6
8	212.9	970	0.2	47205	15.8	10.3
9	213.3	909	0.2	12271	4.1	2.7
10	214.4	856	0.3	5313	1.8	1.2
11	228.2	912	0.3	5147	1.7	1.1
12	249.0	957	0.3	13467	4.5	3.0
13	270.2	1032	0.3	11965	4.0	2.6
14	296.2	858	0.3	299393	100.0	65.6
15	297.2	1047	0.3	23476	7.8	5.1
16	298.5	1149	0.3	8918	3.0	2.0
17	311.9	1486	0.2	7833	2.6	1.7
18	312.3	1414	0.2	8064	2.7	1.8
19	324.3	1454	0.2	5020	1.7	1.1
20	325.1	842	0.4	15632	5.2	3.4
21	326.1	1268	0.3	12122	4.0	2.7
22	329.3	1684	0.2	23686	7.9	5.2
23	329.5	1866	0.2	18314	6.1	4.0
24	330.0	1284	0.3	17960	6.0	3.9
25	331.2	937	0.4	12042	4.0	2.6

Compound Mass Spectrum List Report - MS

#	m/z	Res.	FWHM	I	I %	S/N
26	331.8	1475	0.2	8870	3.0	1.9

Compound Mass Spectrum List Report - MS

+MS2(343.6), 6.3min #464

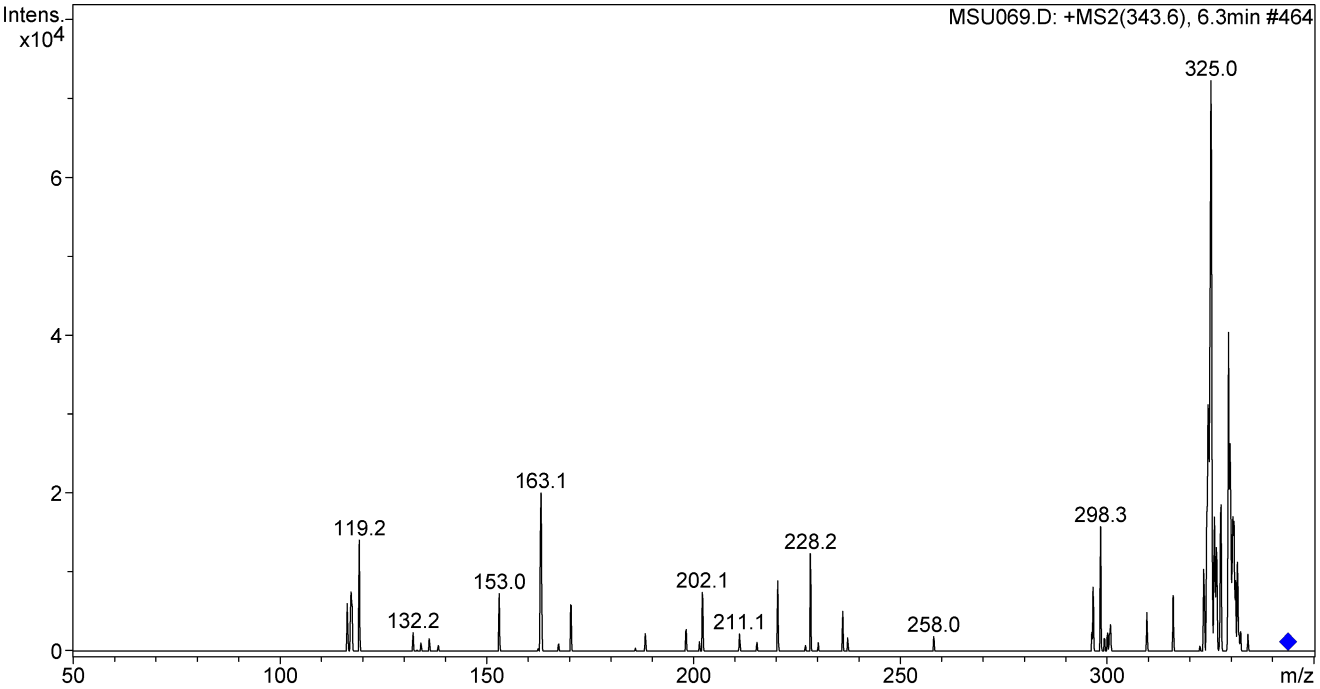

Mass List:

#	m/z	Res.	FWHM	I	I %	S/N
1	116.3	476	0.2	6013	8.3	3.5
2	117.3	237	0.5	7489	10.4	4.4
3	119.2	532	0.2	14050	19.5	8.2
4	132.2	585	0.2	2326	3.2	1.4
5	153.0	679	0.2	7274	10.1	4.2
6	163.1	429	0.4	20008	27.7	11.6
7	170.4	653	0.3	5854	8.1	3.4
8	198.2	774	0.3	2721	3.8	1.6
9	202.1	786	0.3	7452	10.3	4.3
10	220.3	990	0.2	8904	12.3	5.2
11	228.2	1017	0.2	12358	17.1	7.2
12	236.0	1054	0.2	5037	7.0	2.9
13	296.5	1296	0.2	8099	11.2	4.7
14	298.3	1224	0.2	15743	21.8	9.2
15	300.7	647	0.5	3318	4.6	1.9
16	309.5	1369	0.2	4875	6.7	2.8
17	315.9	1218	0.3	7038	9.7	4.1
18	323.3	1007	0.3	10351	14.3	6.0
19	324.3	656	0.5	31171	43.2	18.1
20	325.0	803	0.4	72233	100.0	42.0
21	325.8	1211	0.3	16976	23.5	9.9
22	326.3	1212	0.3	13095	18.1	7.6
23	327.4	949	0.3	18526	25.6	10.8
24	329.2	1350	0.2	40378	55.9	23.5
25	329.6	1418	0.2	26245	36.3	15.3

Compound Mass Spectrum List Report - MS

#	m/z	Res.	FWHM	I	I %	S/N
26	330.3	1512	0.2	17027	23.6	9.9
27	330.5	1569	0.2	16438	22.8	9.6
28	330.8	1978	0.2	8945	12.4	5.2
29	331.4	1097	0.3	11260	15.6	6.5
30	332.1	1484	0.2	2445	3.4	1.4

Compound Mass Spectrum List Report - MS

+MS2(170.3), 7.8min #578

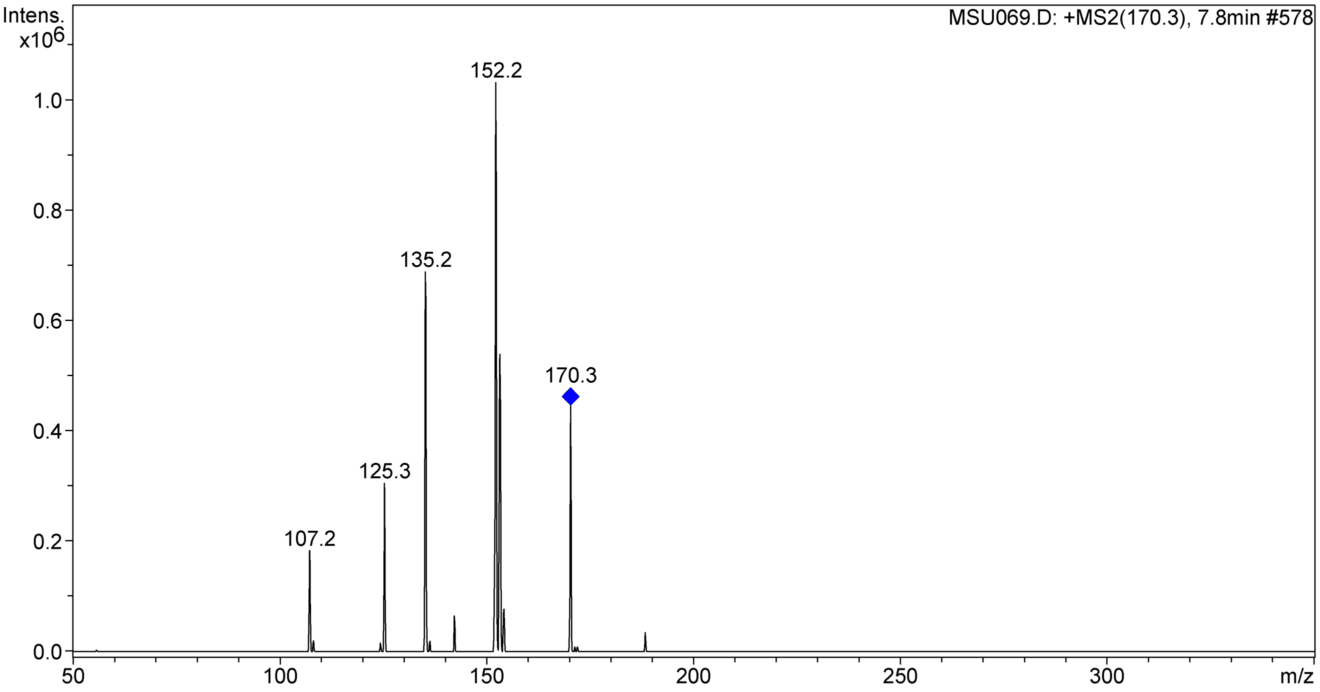

Mass List:

#	m/z	Res.	FWHM	I	I %	S/N
1	107.2	345	0.3	182914	17.7	3.3
2	125.3	463	0.3	304529	29.5	5.5
3	135.2	431	0.3	687989	66.7	12.4
4	142.2	604	0.2	64713	6.3	1.2
5	152.2	499	0.3	1030992	100.0	18.6
6	153.2	379	0.4	538583	52.2	9.7
7	154.1	447	0.3	76834	7.5	1.4
8	170.3	657	0.3	445135	43.2	8.0

Compound Mass Spectrum List Report - MS

+MS2(343.5), 8.2min #608

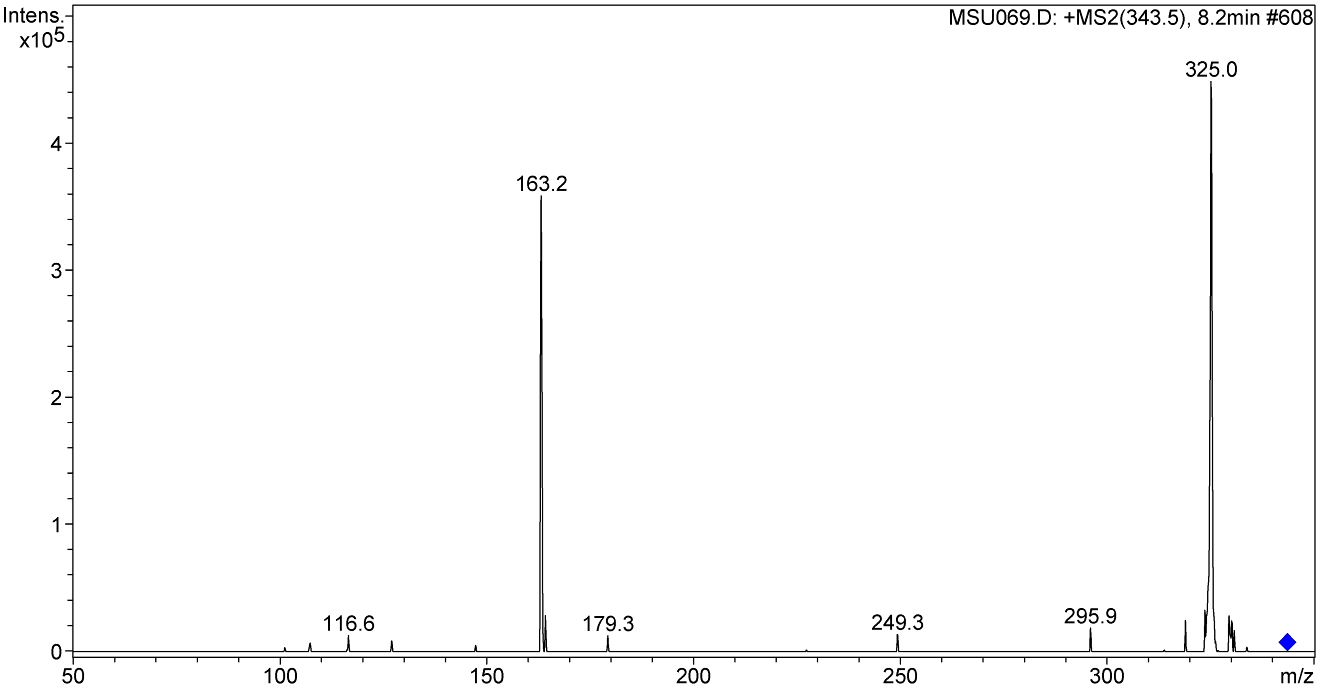

Mass List:

#	m/z	Res.	FWHM	I	I %	S/N
1	107.3	277	0.4	6616	1.5	1.2
2	116.6	515	0.2	12706	2.8	2.3
3	127.1	491	0.3	8408	1.9	1.5
4	163.2	398	0.4	358318	79.9	65.2
5	164.2	701	0.2	28157	6.3	5.1
6	179.3	712	0.3	12496	2.8	2.3
7	249.3	949	0.3	13418	3.0	2.4
8	295.9	1211	0.2	18546	4.1	3.4
9	318.8	1420	0.2	24552	5.5	4.5
10	323.5	1495	0.2	32470	7.2	5.9
11	325.0	659	0.5	448229	100.0	81.6
12	329.4	1271	0.3	28136	6.3	5.1
13	330.0	1432	0.2	23908	5.3	4.4
14	330.6	1471	0.2	16465	3.7	3.0

Compound Mass Spectrum List Report - MS

+MS2(170.5), 10.3min #764

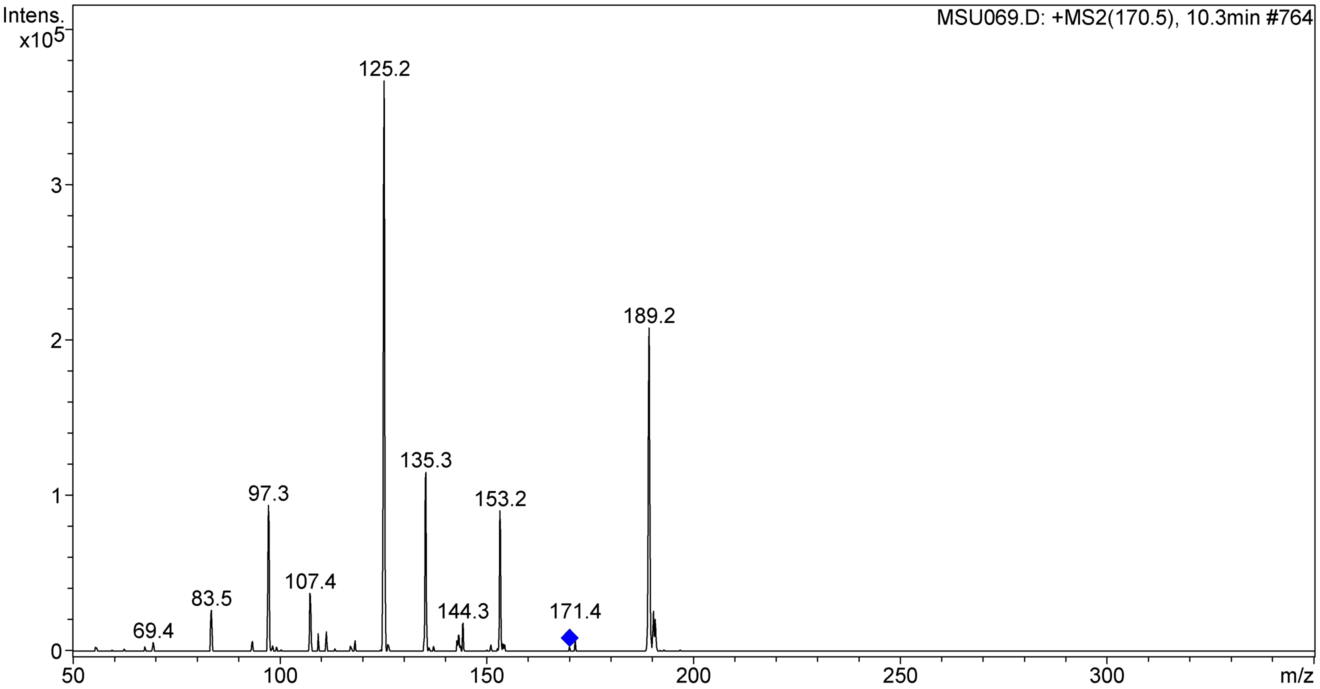

Mass List:

#	m/z	Res.	FWHM	I	I %	S/N
1	55.5	116	0.5	2550	0.7	1.4
2	67.4	281	0.2	2680	0.7	1.4
3	69.4	217	0.3	5421	1.5	2.9
4	83.5	215	0.4	26157	7.1	14.0
5	93.4	287	0.3	6148	1.7	3.3
6	97.3	254	0.4	93612	25.5	50.1
7	98.3	369	0.3	3343	0.9	1.8
8	99.2	398	0.2	2461	0.7	1.3
9	107.4	271	0.4	37176	10.1	19.9
10	109.3	487	0.2	11086	3.0	5.9
11	111.3	420	0.3	12321	3.4	6.6
12	117.1	285	0.4	3124	0.9	1.7
13	118.2	500	0.2	6700	1.8	3.6
14	125.2	334	0.4	366580	100.0	196.2
15	126.2	287	0.4	4111	1.1	2.2
16	135.3	421	0.3	114894	31.3	61.5
17	137.2	541	0.3	3000	0.8	1.6
18	142.9	636	0.2	6545	1.8	3.5
19	143.2	578	0.2	10175	2.8	5.4
20	143.6	605	0.2	3246	0.9	1.7
21	144.3	553	0.3	18044	4.9	9.7
22	151.0	611	0.2	3897	1.1	2.1
23	153.2	485	0.3	90275	24.6	48.3
24	153.9	734	0.2	4617	1.3	2.5
25	154.3	713	0.2	3991	1.1	2.1

Compound Mass Spectrum List Report - MS

#	m/z	Res.	FWHM	I	I %	S/N
26	170.0	763	0.2	2560	0.7	1.4
27	171.4	701	0.2	6213	1.7	3.3
28	189.2	481	0.4	207694	56.7	111.2
29	190.3	729	0.3	25560	7.0	13.7
30	190.7	728	0.3	20316	5.5	10.9

+MS2(293.4), 13.4min #990

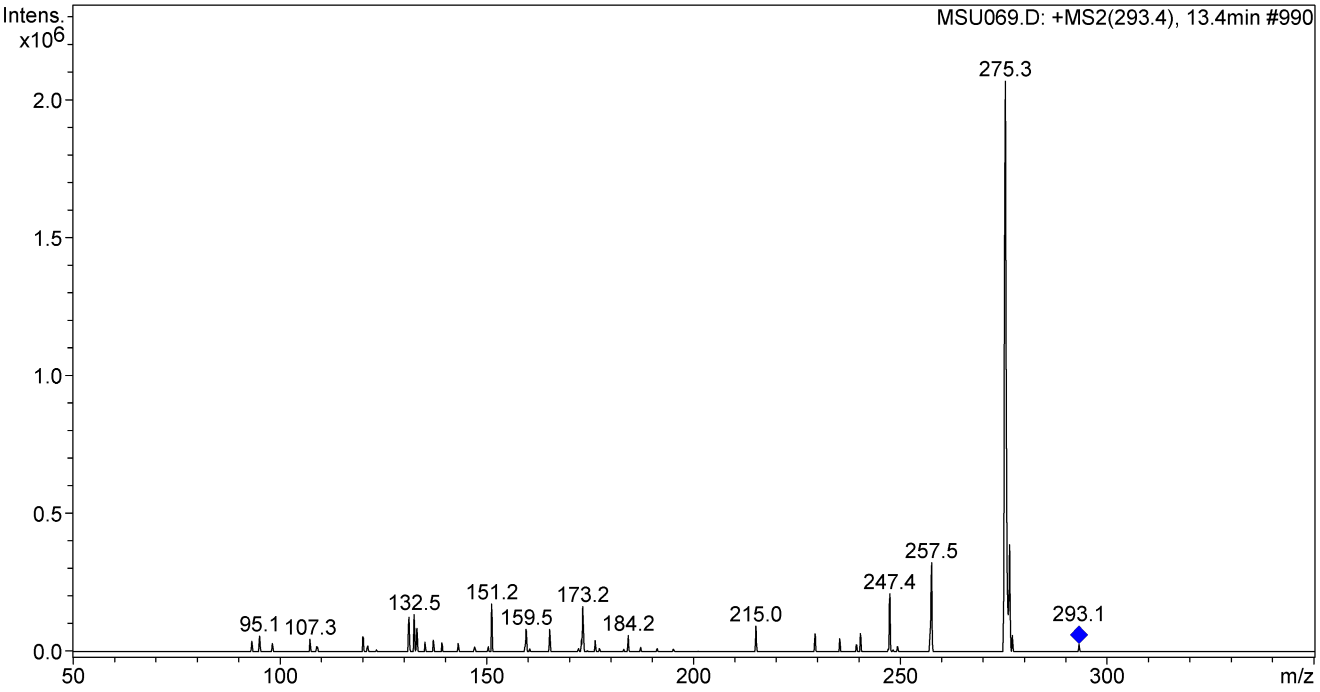

Mass List:

#	m/z	Res.	FWHM	I	I %	S/N
1	93.3	376	0.2	37000	1.8	1.6
2	95.1	369	0.3	56143	2.7	2.5
3	98.2	401	0.2	29258	1.4	1.3
4	107.3	454	0.2	44181	2.1	2.0
5	120.1	472	0.3	53521	2.6	2.4
6	131.2	431	0.3	123843	6.0	5.5
7	132.5	545	0.2	133683	6.5	5.9
8	133.2	483	0.3	83683	4.0	3.7
9	135.1	584	0.2	33781	1.6	1.5
10	137.1	537	0.3	40866	2.0	1.8
11	139.2	627	0.2	30973	1.5	1.4
12	143.1	583	0.2	29613	1.4	1.3
13	151.2	678	0.2	172481	8.3	7.7
14	159.5	582	0.3	80035	3.9	3.6
15	165.2	662	0.2	80009	3.9	3.6
16	173.2	645	0.3	162368	7.9	7.2
17	176.2	785	0.2	40210	1.9	1.8
18	184.2	773	0.2	58333	2.8	2.6
19	215.0	833	0.3	92266	4.5	4.1
20	229.3	854	0.3	63496	3.1	2.8
21	235.3	1063	0.2	46974	2.3	2.1
22	239.3	933	0.3	24603	1.2	1.1
23	240.3	1021	0.2	65013	3.1	2.9
24	247.4	931	0.3	208725	10.1	9.3
25	257.5	920	0.3	321467	15.6	14.3

Compound Mass Spectrum List Report - MS

#	m/z	Res.	FWHM	I	I %	S/N
26	275.3	594	0.5	2066559	100.0	91.7
27	276.3	1038	0.3	386608	18.7	17.2
28	277.0	1261	0.2	58130	2.8	2.6
29	293.1	1289	0.2	27157	1.3	1.2

Compound Mass Spectrum List Report - MS

+MS2(313.2), 13.9min #1030

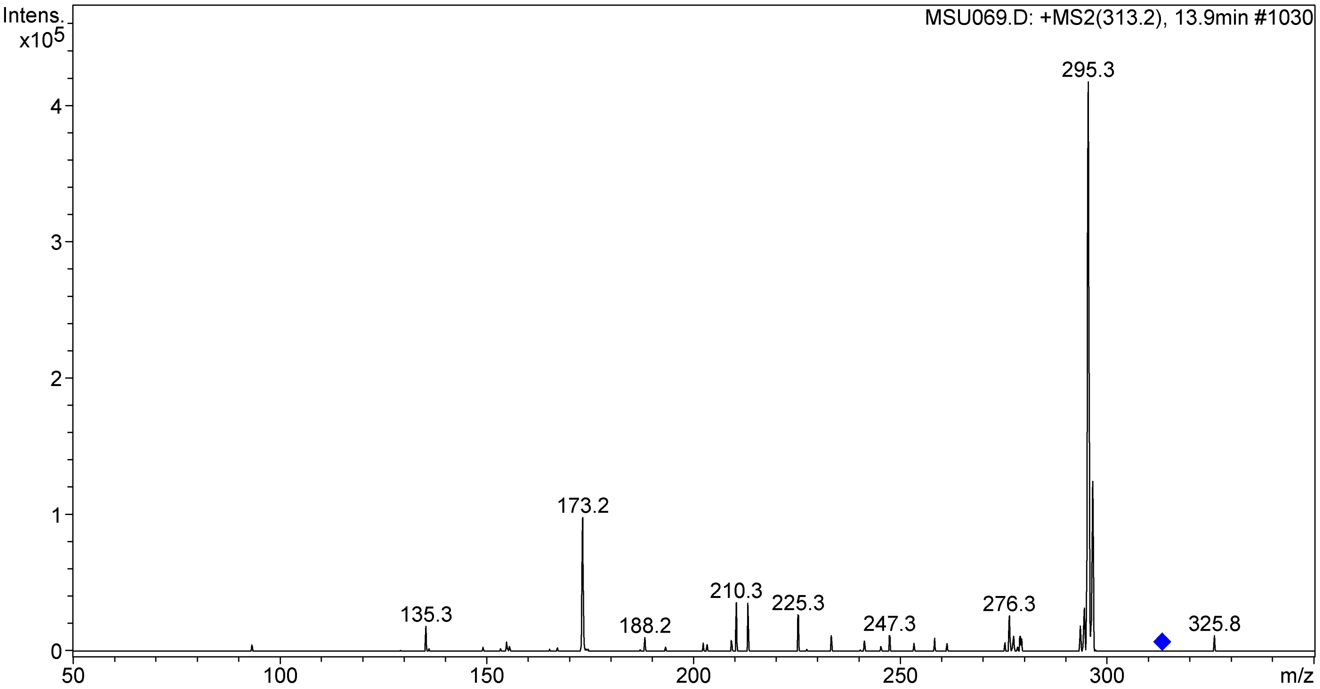

Mass List:

#	m/z	Res.	FWHM	I	I %	S/N
1	135.3	608	0.2	18038	4.3	2.7
2	173.2	534	0.3	97897	23.5	14.7
3	188.2	817	0.2	9870	2.4	1.5
4	209.1	787	0.3	7844	1.9	1.2
5	210.3	932	0.2	35446	8.5	5.3
6	213.1	896	0.2	34916	8.4	5.3
7	225.3	868	0.3	26650	6.4	4.0
8	233.3	915	0.3	11412	2.7	1.7
9	241.3	929	0.3	7356	1.8	1.1
10	247.3	953	0.3	11389	2.7	1.7
11	258.2	1169	0.2	9497	2.3	1.4
12	276.3	894	0.3	25754	6.2	3.9
13	277.3	806	0.3	10727	2.6	1.6
14	278.9	1255	0.2	10921	2.6	1.6
15	279.2	1400	0.2	9005	2.2	1.4
16	293.4	1038	0.3	18389	4.4	2.8
17	294.4	921	0.3	31553	7.6	4.7
18	295.3	716	0.4	417211	100.0	62.8
19	296.4	897	0.3	124299	29.8	18.7
20	325.8	1464	0.2	11287	2.7	1.7

Compound Mass Spectrum List Report - MS

+MS2(313.1), 14.1min #1044

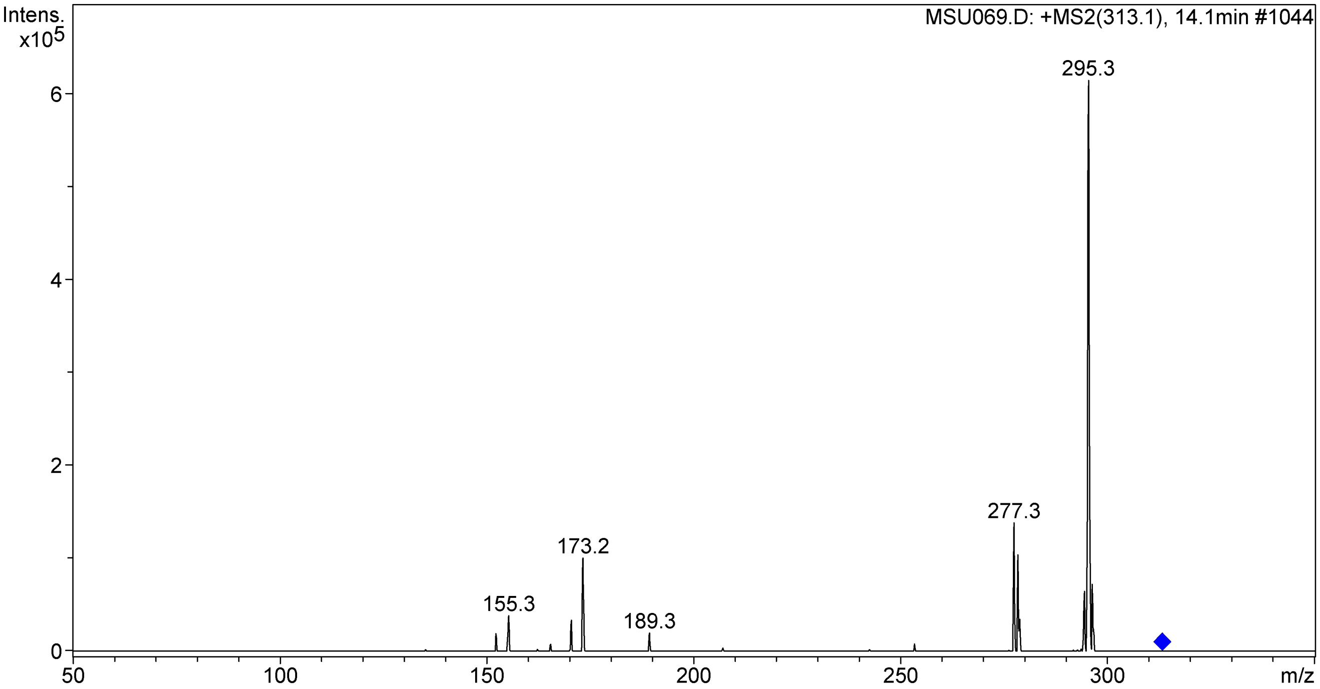

Mass List:

#	m/z	Res.	FWHM	I	I %	S/N
1	152.2	661	0.2	18777	3.1	1.0
2	155.3	433	0.4	38082	6.2	2.0
3	170.4	659	0.3	33185	5.4	1.8
4	173.2	505	0.3	100108	16.3	5.3
5	189.3	729	0.3	19227	3.1	1.0
6	277.3	901	0.3	137914	22.5	7.3
7	278.2	1187	0.2	103324	16.8	5.5
8	278.7	1517	0.2	34283	5.6	1.8
9	294.3	957	0.3	64145	10.4	3.4
10	295.3	708	0.4	613896	100.0	32.7
11	296.2	1235	0.2	71928	11.7	3.8

Compound Mass Spectrum List Report - MS

+MS2(313.6), 14.2min #1058

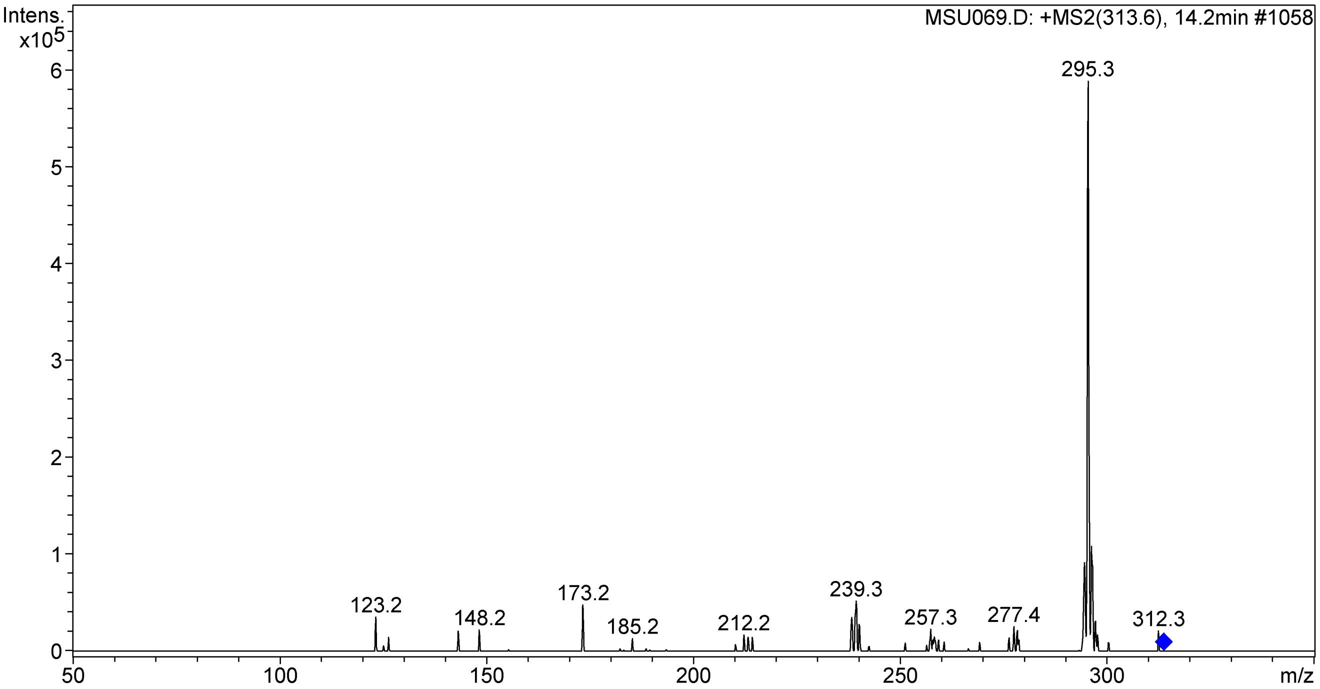

Mass List:

#	m/z	Res.	FWHM	I	I %	S/N
1	123.2	517	0.2	35236	6.0	8.1
2	126.3	560	0.2	14321	2.4	3.3
3	143.1	560	0.3	20632	3.5	4.7
4	148.2	661	0.2	21705	3.7	5.0
5	173.2	624	0.3	47614	8.1	10.9
6	185.2	836	0.2	12888	2.2	3.0
7	212.2	843	0.3	16435	2.8	3.8
8	213.2	720	0.3	14130	2.4	3.2
9	214.2	915	0.2	14298	2.4	3.3
10	238.2	578	0.4	34614	5.9	7.9
11	239.3	475	0.5	51689	8.8	11.9
12	240.0	870	0.3	27623	4.7	6.3
13	251.1	1120	0.2	8281	1.4	1.9
14	257.3	726	0.4	22380	3.8	5.1
15	257.8	1560	0.2	11475	2.0	2.6
16	258.2	576	0.4	14618	2.5	3.4
17	259.2	994	0.3	11250	1.9	2.6
18	260.5	1170	0.2	9225	1.6	2.1
19	269.1	1166	0.2	8958	1.5	2.1
20	276.2	1048	0.3	13575	2.3	3.1
21	277.4	914	0.3	25276	4.3	5.8
22	278.2	1153	0.2	21349	3.6	4.9
23	278.5	1171	0.2	11269	1.9	2.6
24	294.4	708	0.4	91225	15.5	20.9
25	295.3	785	0.4	588158	100.0	134.9

Compound Mass Spectrum List Report - MS

#	m/z	Res.	FWHM	I	I %	S/N
26	296.2	610	0.5	108128	18.4	24.8
27	297.1	1051	0.3	30829	5.2	7.1
28	297.6	1241	0.2	16445	2.8	3.8
29	300.2	1160	0.3	8572	1.5	2.0
30	312.3	1404	0.2	21017	3.6	4.8

Compound Mass Spectrum List Report - MS

+MS2(290.7), 14.8min #1098

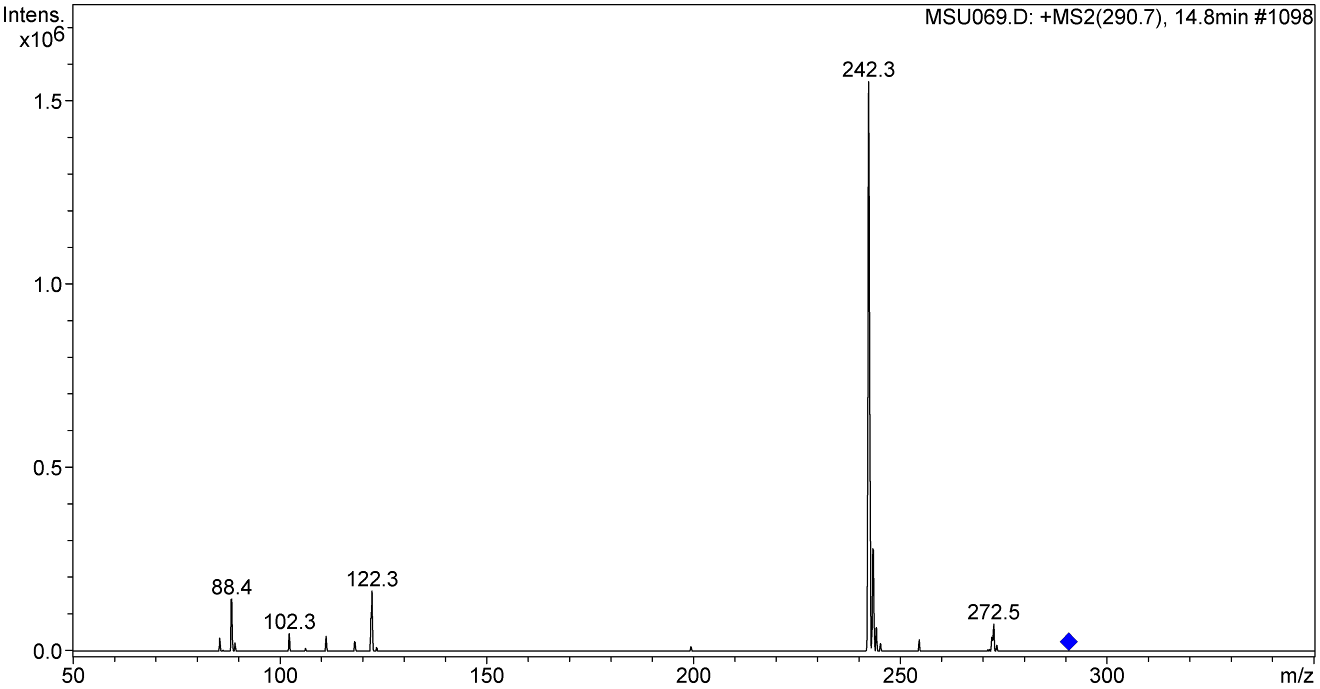

Mass List:

#	m/z	Res.	FWHM	I	I %	S/N
1	85.5	360	0.2	34765	2.2	1.7
2	88.4	298	0.3	141784	9.1	7.0
3	89.2	394	0.2	21958	1.4	1.1
4	102.3	419	0.2	47292	3.0	2.3
5	111.2	461	0.2	40081	2.6	2.0
6	118.2	417	0.3	25321	1.6	1.3
7	122.3	281	0.4	163639	10.5	8.1
8	242.3	571	0.4	1551631	100.0	76.8
9	243.4	663	0.4	278598	18.0	13.8
10	244.1	954	0.3	64024	4.1	3.2
11	245.1	963	0.3	20487	1.3	1.0
12	254.5	1106	0.2	30325	2.0	1.5
13	272.1	1284	0.2	37484	2.4	1.9
14	272.5	1120	0.2	73675	4.7	3.6

Compound Mass Spectrum List Report - MS

+MS2(332.0), 16.0min #1196

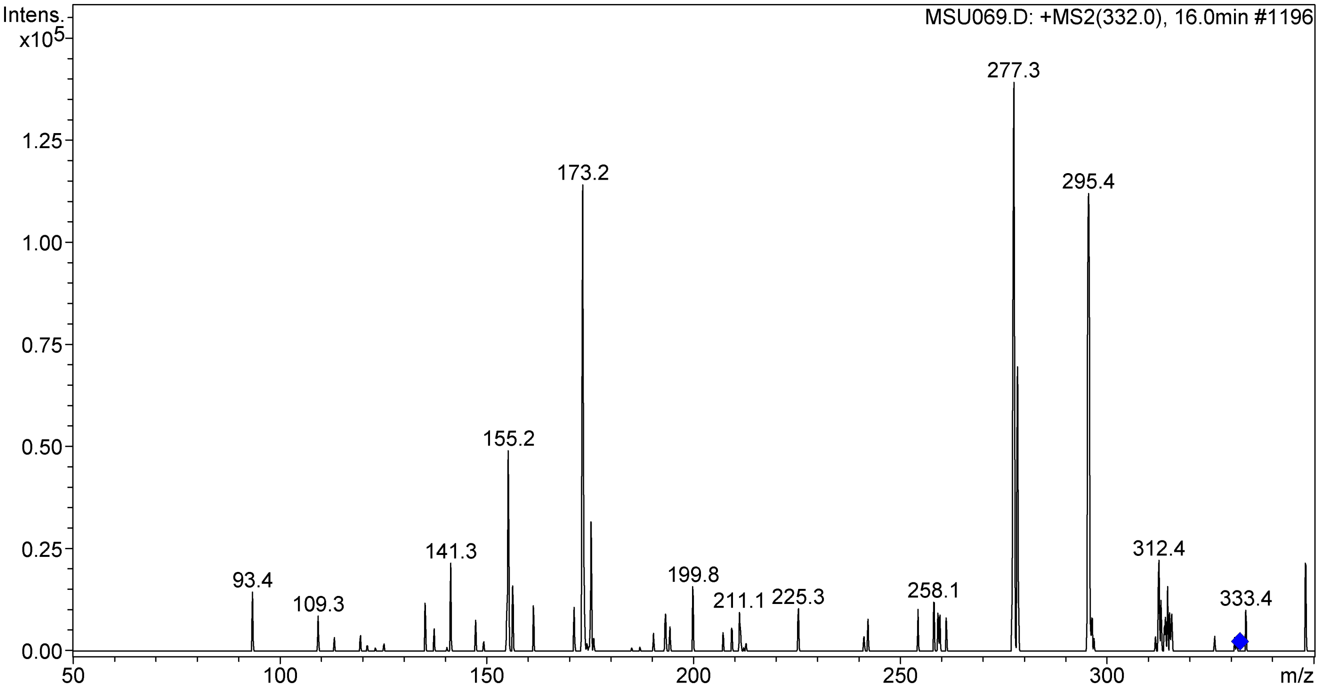

Mass List:

#	m/z	Res.	FWHM	I	I %	S/N
1	93.4	402	0.2	14404	10.4	4.0
2	109.3	424	0.3	8542	6.1	2.4
3	135.1	523	0.3	11703	8.4	3.3
4	141.3	610	0.2	21463	15.4	6.0
5	155.2	484	0.3	49028	35.2	13.7
6	156.3	526	0.3	15903	11.4	4.4
7	161.3	721	0.2	11043	7.9	3.1
8	171.1	727	0.2	10683	7.7	3.0
9	173.2	522	0.3	114004	81.9	31.8
10	175.2	664	0.3	31559	22.7	8.8
11	193.2	594	0.3	9053	6.5	2.5
12	199.8	832	0.2	15783	11.3	4.4
13	211.1	569	0.4	9411	6.8	2.6
14	225.3	767	0.3	10389	7.5	2.9
15	254.2	1140	0.2	10208	7.3	2.8
16	258.1	986	0.3	11949	8.6	3.3
17	259.0	1099	0.2	9225	6.6	2.6
18	259.5	1127	0.2	8822	6.3	2.5
19	277.3	613	0.5	139123	100.0	38.8
20	278.2	827	0.3	69524	50.0	19.4
21	295.4	577	0.5	111898	80.4	31.2
22	296.2	1218	0.2	8103	5.8	2.3
23	312.4	975	0.3	22199	16.0	6.2
24	312.9	1265	0.2	12361	8.9	3.4
25	314.0	1708	0.2	8240	5.9	2.3

Compound Mass Spectrum List Report - MS

#	m/z	Res.	FWHM	I	I %	S/N
26	314.5	1436	0.2	15753	11.3	4.4
27	315.0	1559	0.2	9328	6.7	2.6
28	315.5	1199	0.3	9056	6.5	2.5
29	333.4	1458	0.2	9940	7.1	2.8
30	347.9	1339	0.3	21454	15.4	6.0
