# Supplementary material for: Chitosan Induces Plant Hormones and Defenses in Tomato Root Exudates
Source: Front Plant Sci. 2020 Nov 4;11:572087. doi: 10.3389/fpls.2020.572087 (PMC7672008; doi:10.3389/fpls.2020.572087)
Supplement: Supplementary file 2 [file Data_Sheet_2.docx]

Supplementary Material

**1 Supplementary Tables**

**Supplementary Table 1.** CORCONDIA values of 2, 3 or 4 Components of EEM fluorescence using PARAFAC in MATLAB. Values include all treatmets together per time (2C = two Components, 3C = three Components, 4C = four Components, RE = root exudates, dap = days after planting). CORCONDIA values accepted must be higher than 85.

|  | **Corcondia value** | | |
| --- | --- | --- | --- |
|  | **2 Components** | **3 Components** | **4 Components** |
| RE 3 dap | 99.99 | 85.94 | 45.59 |
| RE 10 dap | 100 | 90 | 73.30 |
| RE 20 dap | 100 | 98.69 | 76.22 |
| RE 30 dap | 100 | -0.63 | -3.89 |

**Supplementary Table 2.** Peak assignments for ^1^H NMR spectra of tomato root exudates. Abbreviations: dap = days after planting, ^1^H mult. = ^1^H multiplicity, d = doublet, s = singlet, t = triplet, dt = double of triplets, m = multiplet. All peaks are present in both treatments, but not at all times.

| **Peak number** | **Compound** | **Shift (ppm)** | **^1^H mult.** | **10 dap** | **20 dap** | **30 dap** |
| --- | --- | --- | --- | --- | --- | --- |
| 1 | Unknown | 0.083 | s | X |  | X |
| 2 | Unknown | 0.104 | s | X |  |  |
| 3 | Unknown | 0.17 | s | X | X | X |
| 4 | Unknown | 0.83 | s |  | X |  |
| 5 | Unknown | 0.84 | s |  | X |  |
| 6 | Unknown | 0.85 | s |  | X |  |
| 7 | Unknown | 0.86 | s |  | X |  |
| 8 | Unknown | 0.87 | s |  | X |  |
| 9 | Unknown | 0.88 | s |  | X |  |
| 10 | Unknown | 0.89 | s |  | X |  |
| 11 | Unknown | 0.91 | d | X | X |  |
| 12 | Unknown | 0.93 |  |  | X |  |
| 13 | Leucine/Isoleucine | 0.94 |  |  | X |  |
| 14 | Unknown | 0.95 | s |  | X |  |
| 15 | Unknown | 0.96 | d |  | X |  |
| 16 | Unknown | 0.98 |  |  | X |  |
| 17 | Unknown | 0.99 | s |  | X |  |
| 18 | Unknown | 1 | d |  | X |  |
| 19 | Unknown | 1.03 | s |  | X |  |
| 20 | Unknown | 1.04 | s |  | X |  |
| 21 | Unknown | 1.05 |  |  | X |  |
| 22 | Unknown | 1.06 | d | X | X |  |
| 23 | Unknown | 1.07 | s |  | X |  |
| 24 | Unknown | 1.08 | s |  | X |  |
| 25 | Unknown | 1.1 | s | X | X | X |
| 26 | Unknown | 1.15 | d | X | X | X |
| 27 | Unknown | 1.18 | s | X | X | X |
| 28 | Unknown | 1.19 | d | X | X | X |
| 29 | Unknown | 1.206 | s | X |  |  |
| 30 | Unknown | 1.213 | s | X |  |  |
| 31 | Unknown | 1.23 |  | X | X |  |
| 32 | Unknown | 1.24 |  | X | X |  |
| 33 | Unknown | 1.251 |  | X | X |  |
| 34 | Unknown | 1,255 |  | X | X | X |
| 35 | Unknown | 1.27 |  |  | X |  |
| 36 | Lactate | 1.33 | s | X | X | X |
| 37 | Unknown | 1.34 | s | X | X |  |
| 38 | Unknown | 1.38 | d |  | X | X |
| 39 | Unknown | 1.39 |  |  | X |  |
| 40 | Unknown | 1.43 | d |  | X | X |
| 41 | Unknown | 1.44 |  |  | X |  |
| 42 | Unknown | 1.46 |  |  | X |  |
| 43 | Unknown | 1.47 |  |  | X |  |
| 44 | Unknown | 1.495 | d |  | X |  |
| 45 | Unknown | 1.626 | d |  | X |  |
| 46 | Unknown | 1.78 | d |  | X |  |
| 47 | Unknown | 1.83 |  |  | X |  |
| 48 | Unknown | 1.88 | s |  | X |  |
| 49 | Unknown | 1.89 | s |  | X |  |
| 50 | Unknown | 1.9 | s |  | X |  |
| 51 | Unknown | 1.91 | s | X | X |  |
| 52 | Unknown | 1.92 | s |  | X | X |
| 53 | Acetate | 1.948 |  |  | X |  |
| 54 | Unknown | 2 |  |  | X |  |
| 55 | Unknown | 2.05 | d |  | X |  |
| 56 | Unknown | 2.07 | s | X | X | X |
| 57 | Unknown | 2.14 | s | X | X |  |
| 58 | Unknown | 2.17 |  |  | X |  |
| 59 | Unknown | 2.18 |  |  | X | X |
| 60 | Unknown | 2.19 |  |  | X |  |
| 61 | Unknown | 2.239 |  |  |  | X |
| 62 | Malate | 2.3 | t | X | X | X |
| 63 | Unknown | 2.34 |  |  | X |  |
| 64 | Malate | 2.41 |  | X | X | X |
| 65 | Unknown | 2.6 |  | X | X |  |
| 66 | Unknown | 2.74 |  |  | X |  |
| 67 | Unknown | 2.92 | d |  | X |  |
| 68 | Unknown | 2.94 | s | X | X |  |
| 69 | Unknown | 2.96 | d |  | X |  |
| 70 | Unknown | 3 | t |  | X |  |
| 71 | Unknown | 3.04 | s |  | X |  |
| 72 | Unknown | 3.13 | s | X | X |  |
| 73 | Unknown | 3.21 | s | X | X |  |
| 74 | Unknown | 3.23 |  | X |  |  |
| 75 | Unknown | 3.24 | d | X |  |  |
| 76 | Unknown | 3.25 | d | X |  |  |
| 77 | Unknown | 3.27 | s | X | X |  |
| 78 | Unknown | 3.28 |  | X | X |  |
| 79 | Unknown | 3.3 | s |  | X |  |
| 80 | Metanol | 3.36 | s |  | X | X |
| 81 | Sugar Moyeti | 3.45-4.35 |  |  | X |  |
| 82 | Unknown | 4.376 |  |  | X |  |
| 83 | Unknown | 4.39 |  |  | X |  |
| 84 | Unknown | 4.4 |  |  | X |  |
| 85 | Unknown | 4.42 | d |  | X |  |
| 86 | Unknown | 4.436 |  |  | X |  |
| 87 | Unknown | 4.445 | s |  | X |  |
| 88 | Unknown | 4.47 |  |  | X |  |
| 89 | Unknown | 4.485 |  |  | X |  |
| 90 | Unknown | 4.52 | t |  | X |  |
| 91 | Unknown | 4.59 | d |  | X |  |
| 92 | Unknown | 4.61 |  |  | X |  |
| 93 | Unknown | 4.63 |  |  | X |  |
| 94 | Unknown | 4.65 | d |  | X |  |
| 95 | Unknown | 4.78 |  |  | X |  |
| 96 | Unknown | 4.906 | d |  | X |  |
| 97 | Unknown | 4.93 | d |  | X |  |
| 98 | Unknown | 4.95 | d |  | X |  |
| 99 | Rafinosa | 4.99 | d |  | X |  |
| 100 | Glucosa | 5.237 | d |  | X |  |
| 101 | Unknown | 5.252 | s |  | X |  |
| 102 | Unknown | 5.268 | d |  | X |  |
| 103 | Rafinosa | 5.42 | d |  | X |  |
| 104 | Unknown | 5.498 | d |  | X |  |
| 105 | Uracil | 5.79 | d |  | X |  |
| 106 | Unknown | 5.88 | d |  | X |  |
| 107 | Unknown | 5.98 | d |  | X |  |
| 108 | Unknown | 6.157 | m |  | X |  |
| 109 | Cinnamic Acid / Fumaric Acid | 6.52 / 6.51 | d / s |  | X |  |
| 110 | Unknown | 6.589 |  |  | X |  |
| 111 | Unknown | 6.634 | s |  | X |  |
| 112 | p-Aminobenzoic Acid | 6.82 | d |  | X |  |
| 113 | Unknown | 6.85 | m |  | X |  |
| 114 | Unknown | 7.09 | s |  | X |  |
| 115 | Unknown | 7.16 |  |  | X |  |
| 116 | Unknown | 7.31 |  |  | X |  |
| 117 | Unknown | 7.45 |  |  | X |  |
| 118 | Unknown | 7.4 |  |  | X |  |
| 119 | Cinnamic Acid | 7.43 | m |  | X |  |
| 120 | Uracil | 7.52 | d |  | X |  |
| 121 | Cinnamic Acid | 7.62 | dt |  | X |  |
| 122 | p-Aminobenzoic Acid | 7.73 | d |  | X |  |
| 123 | Unknown | 7.81 |  |  | X |  |
| 124 | Unknown | 7.87 |  |  | X |  |
| 125 | Trigonelline | 8.07 | m |  | X |  |
| 126 | Unknown | 8.08 | t |  | X |  |
| 127 | Unknown | 8.11 | s |  | X |  |
| 128 | Unknown | 8.15 |  |  | X |  |
| 129 | Formic Acid | 8.44 | m |  | X | X |
| 130 | Trigonelline | 8.82 | m |  | X |  |
| 131 | Trigonelline | 9.13 | s |  | X |  |

**Supplementary Table 3.** HPLC-ESI-MS significant data (m/z signals) of tomato root exudates respect to control. Positive and negative modes. Multifactorial analysis 2way ANOVA and Fisher’s LSD test were used to compare treatments (p-values 0.033 (*), 0.002 (**), 0.0002(***) and <0.0001(****)). Numbers in treatments correspond to the mean of maximum heights of the peaks (arbitrary units).

|  |  | **[Chitosan] (mg·mL^-1^)** | |
| --- | --- | --- | --- |
| **Compound** | **m/z (+)** | **0** | **0.1** |
| Undetermined | 85.7 | 0.444 ± 0.119 | 0.634 ± 0.154 |
| Undetermined | 170.6 | 0.373 ± 0.028 | 0.222 ± 0.066 |
| Undetermined | 274.6 | 0.184 ± 0.042 | 0.156 ± 0.011 |
| Undetermined | 275.7 | 0.110 ± 0.010 | 0.216 ± 0.038 |
| 3'-O-Methyladenosine | 282.3 | 0.303 ± 0.138 | 0.198 ± 0.012 |
| Atropine / Hyoscyamine | 290.5 | 0.835 ± 0.133 | 1.009 ± 0.409 |
| FA18:4+1O | 293.5 | 0.221 ± 0.017 | 0.395 ± 0.056 |
| Undetermined | 297.7 | 0.390 ± 0.073 | 0.265 ± 0.043 |
| Ethyl stearate / Arachidic acid | 313.7 | 0.676 ± 0.111 | 0.911 ± 0.012 |
| Undetermined | 331.8 | 0.731 ± 0.084 | 1.048 ± 0.042** |
| Fructose 1,6-bisphosphate | 341.0 | 0.111 ± 0.109 | 0.249 ± 0.041 |
| Undetermined | 342.7 | 0.317 ± 0.433 | 0.881 ± 0.043**** |
| N-Fructosyl tyrosine | 344.3 | 0.219 ± 0.044 | 0.118 ± 0.004 |
|  |  |  |  |
|  |  | **[Chitosan] (mg·mL^-1^)** | |
| **Compound** | **m/z (-)** | **0** | **0.1** |
| Undetermined | 133.5 | 0.060 ± 0.008 | 0.102 ± 0.066 |
| Undetermined | 165.5 | 0.012 ± 0.006 | 0.060 ± 0.023 |
| Undetermined | 187.6 | 0.053 ± 0.002 | 0.038 ± 0.005 |
| Citric Acid | 191.0 | 0.347 ± 0.081 | 0.393 ± 0.088 |
| Undetermined | 309.7 | 0.007 ± 0.005 | 0.004 ± 0.005 |
| Undetermined | 311.7 | 0.030 ± 0.013 | 0.031 ± 0.009 |
| FA 18:2+3O | 327.3 | 0.496 ± 0.163 | 0.246 ± 0.140* |
| Undetermined | 329.7 | 0.264 ± 0.145 | 0.161 ± 0.021 |

**Supplementary Table 4.** HPLC-ESI-MS/MS m/z characteristic ions and precursor mass of putative identified compounds in tomato root exudates. Positive and negative modes. Databases used to compare spectra were HMDB and MassBank.

| **Putative Compound** | **Mode** | **Precursor Mass** | **Characteristic Ions** | **Annotation Score (%)** | **Database Reference** |
| --- | --- | --- | --- | --- | --- |
| Citric Acid | - | 191.0 | **110.9**  111.9  128.8  130.9  172.9  173.9 | **100**  6.4  3.1  1.7  24.0  3.4 | HMDB:  HMDB0000094 |
| 3'-O-Methyladenosine  2'-O-Methyladenosine | + | 282.3 | 81.4  97.4  **136.2**  137.2  138.2  147.2  149.1  211.2  245.5  263.3  264.5  265.3  298.6 | 2.2  3.1  **100**  12  2.1  1  1.1  1.3  2.4  3.4  1.1  8.4  4.3 | HMDB: HMDB0006023  HMDB0004326  MassBank:  RP031201 |
| Atropine / Hyoscyamine | + | 290.5 | 97.4  118.2  122.0  189.0  211.2  **242.3**  243.3  246.3  272.3  301.6 | 4.7  8.7  23.7  2.7  4.4  **100**  25.7  4.1  13.7  2.9 | HMDB: HMDB0014712  HMDB0014568 |
| FA 18:4+1O | + | 293.4 | 93.3  95.1  98.2  107.3  120.1  131.2  132.5  133.2  135.1  137.1  139.2  143.1  151.2  159.5  165.2  173.2  176.2  184.2  215.0  229.3  235.3  239.3  240.3  247.4  257.5  **275.3**  276.3  277.0  293.1 | 1.8  2.7  1.4  2.1  2.6  6  6.5  4  1.6  2  1.5  1.4  8.3  3.9  3.9  7.9  1.9  2.8  4.5  3.1  2.3  1.2  3.1  10.1  15.6  **100**  18.7  2.8  1.3 | MassBank:  PR310790 |
| Ethyl stearate / Arachidic acid | + | 313.7 | 168.2  196.3  213.1  217.4  238.2  239.2  259.3  266.4  267.3  268.3  275.2  277.3  293.3  294.3  **295.3**  296.2  297.2 | 0.9  1  0.9  2.9  2.4  8.6  1.8  7.1  8.3  2.9  1.5  6.8  4.1  43.7  **100**  19.9  2.6 | HMDB:  HMDB0034156  HMDB0002212 |
| FA 18:2+3O | - | 327.3 (13.3 min) | 149.0  154.9  164.8  **171.0**  171.8  185.0  193.0  199.7  200.7  201.0  201.9  203.0  208.8  210.9  228.8  239.0  240.9  242.8  263.1  290.8  291.2  291.9  309.1  310.1 | 3.9  4.3  10.8  **100**  23.9  3.3  8.8  9.1  6.8  11.9  6.3  6.4  5.1  15.2  20  6  4.5  2.6  4.6  8.2  14.2  4.1  19.8  3.3 | MassBank:  PR309098 |
| Fructose 1,6-bisphosphate | + | 341.0 | 102.4  124.0  130.2  **133.1**  146.3  151.1  155.3  165.3  177.2  198.7  199.2  221.2  221.9  243.2  243.8  265.3  287.0  293.0  296.5  297.5  305.4  307.0  308.2  309.3  318.2  322.1 | 17.6  14.2  12.9  **100**  16.8  12.2  5.8  8.3  39.1  14.9  54  65.1  5.6  68.7  26.5  49.6  14.6  15.1  8.6  8.6  6.8  7.5  23.2  25.6  23.3  6.9 | HMDB:  HMDB0001058 |
| N-Fructosyl tyrosine | + | 344.3 | 116.2  119.3  129.2  133.2  139.1  147.2  148.2  166.3  177.2  182.3  183.9  188.0  200.1  210.2  221.2  223.2  239.4  309.1  309.8  324.5  325.1  325.7  326.2  **327.3**  328.3  329.2  329.7  330.1  331.2 | 1.5  0.5  0.8  1.3  1.1  1.9  0.7  1.1  0.6  0.7  1.5  0.4  1.6  1.2  4  0.4  1  3.8  0.4  2  0.4  0.6  1.2  **100**  10.8  1.8  1.8  0.7  0.7 | MassBank:  PR310827 |

**2 Supplementary Figures**

**
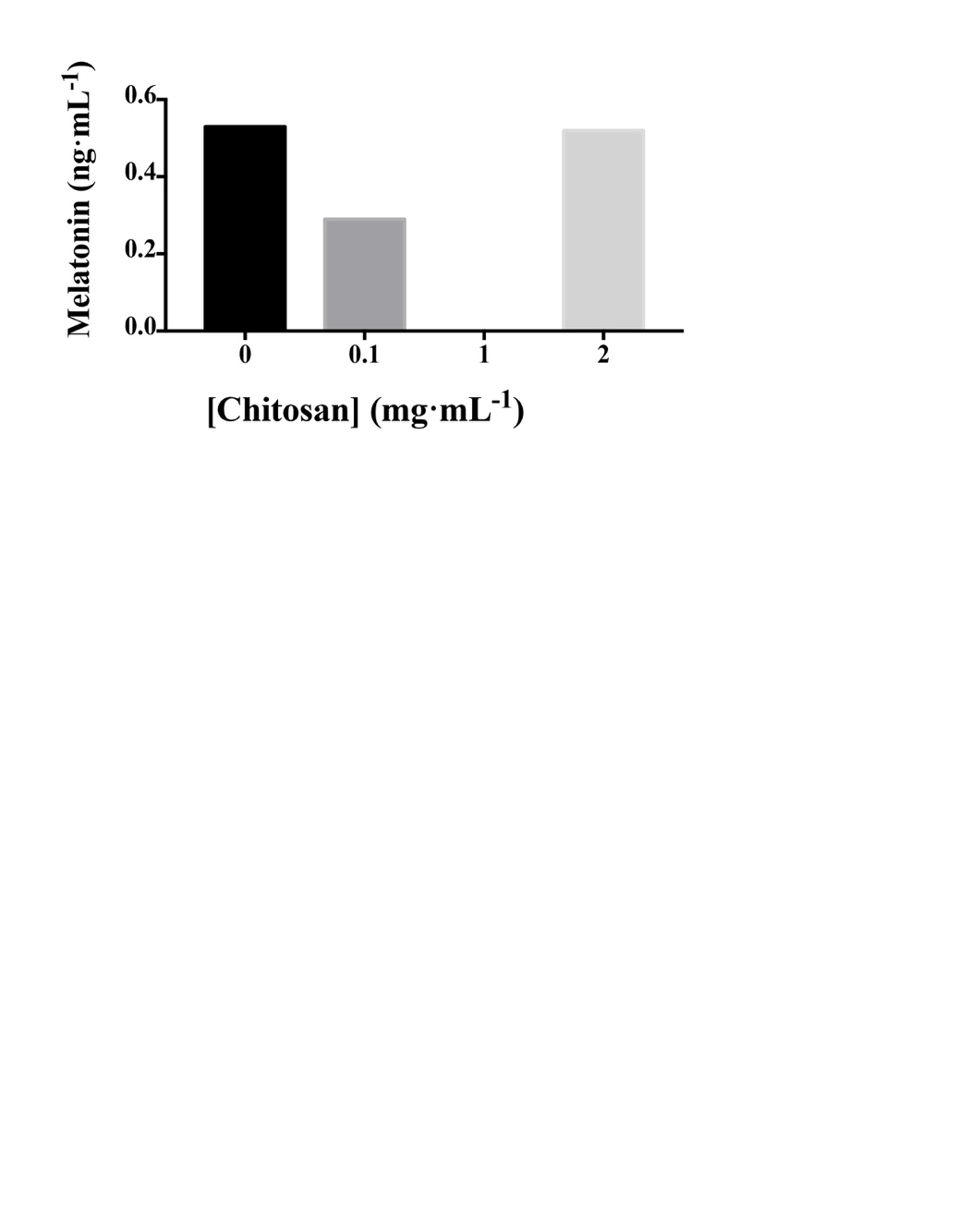
**

**Supplementary Figure 1.** Phytomelatonin quantification by HPLC in tomato root exudates of 20-day old plants exposed 3 days to chitosan. N=3.

**
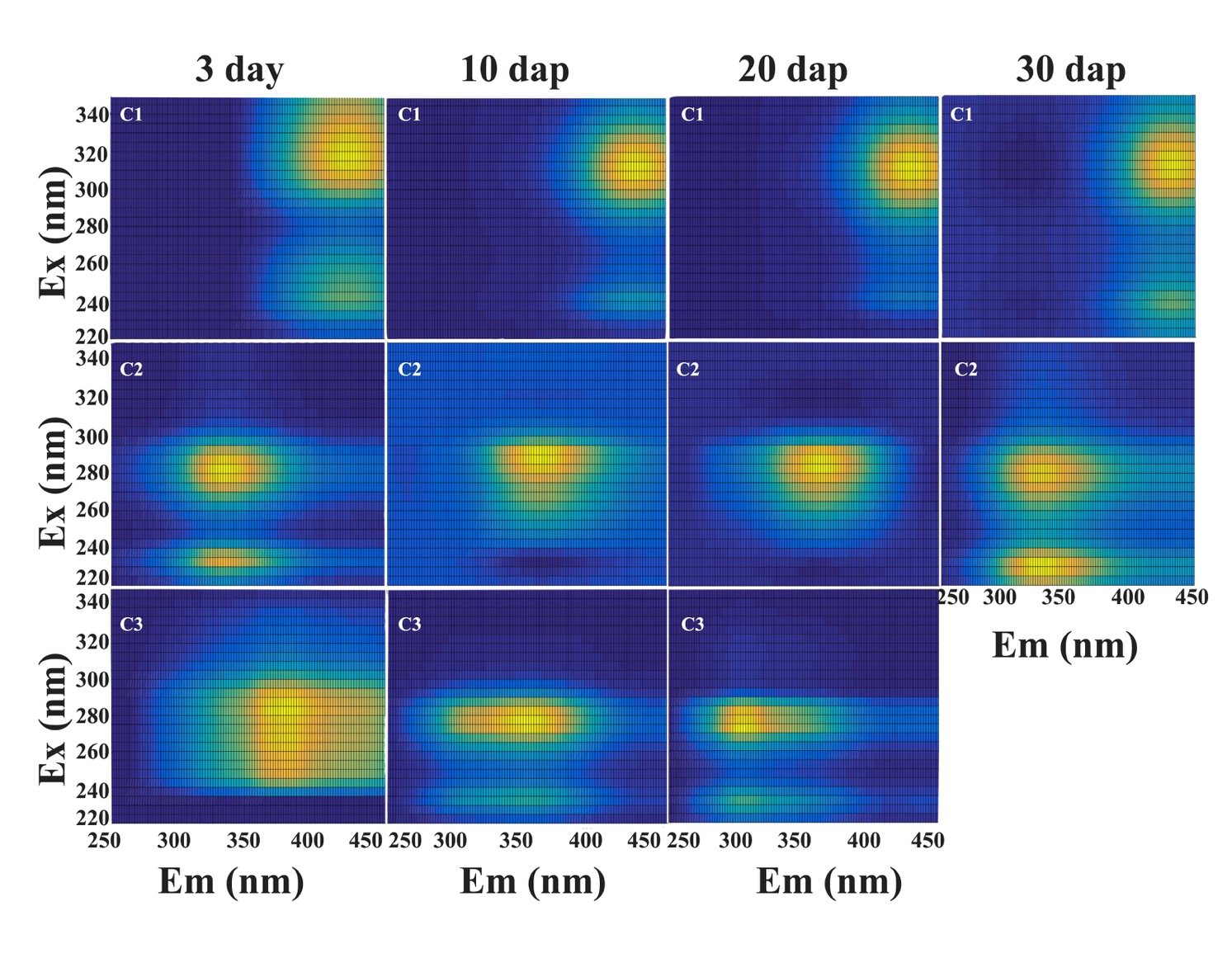
**

**Supplementary Figure 2.** Time course of EEM Fluorescence maps of tomato root exudates. No distinctions are made for treatments. For Ex/Em component coordinates see Table 1. Abbreviations: EEM = Emission Excitation Matrix, dap = days after planting, C1 = Component 1, C2 = Component 2, C3 = Component 3.

**
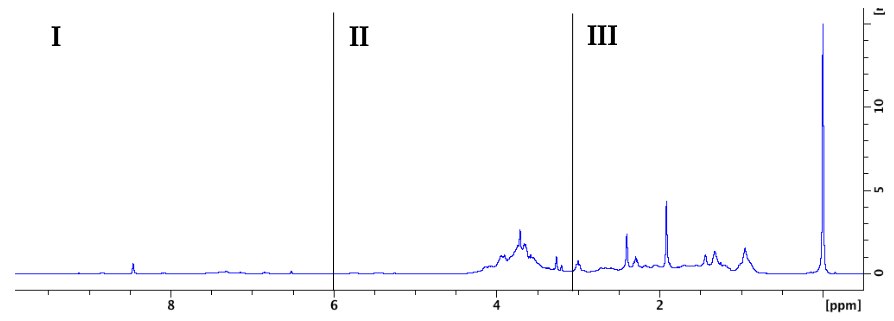
**

**Supplementary Figure 3.** ^1^H NMR spectrum of tomato root exudates. Spectrum is divided in three regions: organic-acid and amino acid region (I), sugar/polyalcohol region (II) and phenolic/aromatic region (III).

**3 Large scale datasets of HPLC-ESI-MS were deposited in Dryad database and are accessible following this link:**

https://datadryad.org/stash/share/iQ4Fh9XTzpF5Rbsgz9dakjCP2BfvpSigHx-hhuy57GY

Suarez-Fernandez, Marta et al. (2020), HPLC-ESI-MS Root Exudates Chitosan, Dryad, Dataset, <https://doi.org/10.5061/dryad.ghx3ffbkb>
